# New Inhibitors of Bcr-Abl Based on 2,6,9-Trisubstituted Purine Scaffold Elicit Cytotoxicity in Chronic Myeloid Leukemia-Derived Cell Lines Sensitive and Resistant to TKIs

**DOI:** 10.3390/pharmaceutics16050649

**Published:** 2024-05-11

**Authors:** Thalia Delgado, Denisa Veselá, Hana Dostálová, Vladimír Kryštof, Veronika Vojáčková, Radek Jorda, Alejandro Castro, Jeanluc Bertrand, Gildardo Rivera, Mario Faúndez, Miroslav Strnad, Christian Espinosa-Bustos, Cristian O. Salas

**Affiliations:** 1 Departamento de Química Orgánica, Facultad de Química y de Farmacia, Pontificia Universidad Católica de Chile, Santiago de Chile 702843, Chile; tdelgado@uc.cl (T.D.); jgbertrand@uc.cl (J.B.); 2 Department of Experimental Biology, Palacký University Olomouc, Šlechtitelů 27, 783 71 Olomouc, Czech Republic; denisa.vesela@upol.cz (D.V.); hana.dostalova@upol.cz (H.D.); veronika.vojackova@upol.cz (V.V.); radek.jorda@upol.cz (R.J.); 3 Institute of Molecular and Translational Medicine, Faculty of Medicine and Dentistry, Palacký University Olomouc, Hněvotínská 5, 779 00 Olomouc, Czech Republic; 4 Laboratorio de Bioproductos Farmacéuticos y Cosméticos, Centro de Excelencia en Medicina Traslacional, Facultad de Medicina, Universidad de La Frontera, Av. Francisco Salazar 01145, Temuco 4780000, Chile; alejandro.castro.a@ufrontera.cl; 5 Laboratorio de Biotecnología Farmacéutica, Centro de Biotecnología Genómica, Instituto Politécnico Nacional, Boulevard del Maestro s/n, Reynosa 88710, Mexico; giriveras@ipn.mx; 6 Departamento de Farmacia, Facultad de Química y de Farmacia, Pontificia Universidad Católica de Chile, Santiago de Chile 702843, Chile; mfaundeza@uc.cl (M.F.); ccespino@uc.cl (C.E.-B.); 7 Laboratory of Growth Regulators, Institute of Experimental Botany of the Czech Academy of Sciences & Palacký University, Šlechtitelů 27, 783 71 Olomouc, Czech Republic; miroslav.strnad@upol.cz

**Keywords:** chronic myeloid leukemia, TKI-resistant cells, Bcr-Abl inhibitors, purine derivatives, in silico studies

## Abstract

Bcr-Abl is an oncoprotein with aberrant tyrosine kinase activity involved in the progression of chronic myeloid leukemia (CML) and has been targeted by inhibitors such as imatinib and nilotinib. However, despite their efficacy in the treatment of CML, a mechanism of resistance to these drugs associated with mutations in the kinase region has emerged. Therefore, in this work, we report the synthesis of 14 new 2,6,9-trisubstituted purines designed from our previous Bcr-Abl inhibitors. Here, we highlight **11b**, which showed higher potency against Bcr-Abl (IC_50_ = 0.015 μM) than imatinib and nilotinib and exerted the most potent antiproliferative properties on three CML cells harboring the Bcr-Abl rearrangement (GI_50_ = 0.7–1.3 μM). In addition, these purines were able to inhibit the growth of KCL22 cell lines expressing Bcr-Abl^T315I^, Bcr-Abl^E255K^, and Bcr-Abl^Y253H^ point mutants in micromolar concentrations. Imatinib and nilotinib were ineffective in inhibiting the growth of KCL22 cells expressing Bcr-Abl^T315I^ (GI_50_ > 20 μM) compared to **11b**–**f** (GI_50_ = 6.4–11.5 μM). Molecular docking studies explained the structure–activity relationship of these purines in Bcr-Abl^WT^ and Bcr-Abl^T315I^. Finally, cell cycle cytometry assays and immunodetection showed that **11b** arrested the cells in G1 phase, and that **11b** downregulated the protein levels downstream of Bcr-Abl in these cells.

## 1. Introduction

Leukemia, a hematological malignancy, involves the uncontrolled proliferation of abnormal white blood cells. This neoplastic condition results from genetic mutations disrupting hematopoiesis and poses a significant healthcare challenge [1]. Among these mutations, the *BCR:ABL* fusion gene stands out, a product of the combination between the Abelson (*ABL*) tyrosine kinase gene on chromosome 9 and the break-point cluster (*BCR*) gene on chromosome 22 [2]. This genetic abnormality is a hallmark molecular feature of chronic myeloid leukemia (CML) and a significant contributing factor to other leukemia subtypes, such as B-cell precursor-positive acute lymphoblastic leukemia (ALL), promoting the pathogenesis of these diseases. The oncoprotein Bcr-Abl, a mutated tyrosine kinase (TK), contributes to the progression and persistence of leukemia by interacting with numerous downstream signaling pathways. These interactions lead to modifications in cellular adhesion, stimulation of mitogenic signaling, and suppression of apoptosis, ultimately resulting in the malignant transformation of hematopoietic stem cells [2,3,4].

**Figure 1:**
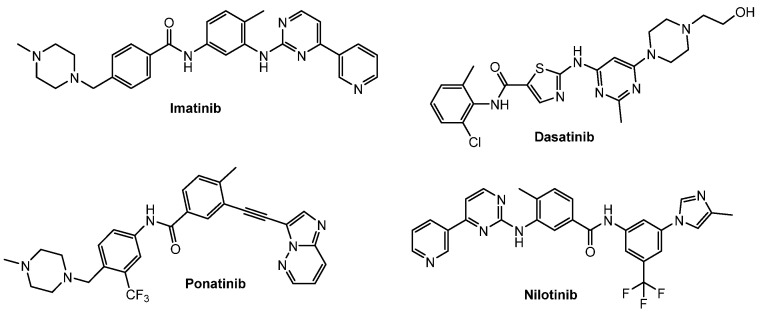
Chemical structures of Bcr-Abl inhibitors approved by the FDA for TKI-based therapy.

To overcome this challenge, tyrosine kinase inhibitors (TKIs) represent the gold standard for the treatment of leukemia. The development of a small molecule with the ability to block Bcr-Abl activity dramatically impacts the disease’s management. The TKI impairs the interaction of the oncoprotein with ATP, thereby blocking cell signals and, consequently, reducing cell proliferation and inducing cell death [5]. Imatinib was the first TKI approved by the FDA that had efficacy in treating patients with the wild-type (WT) *BCR:ABL* gene (Figure 1). However, due to persistent resistance to this treatment, the need arose to develop second-generation TKIs, such as dasatinib and nilotinib, as well as third-generation TKIs like ponatinib (Figure 1) [2,5,6].

Despite the successes achieved by the existing TKI-based therapy so far, resistance remains an obstacle. A considerable number of patients treated with imatinib (20–30%) and <10% of patients treated with second-generation TKIs show intrinsic or acquired resistance to treatment during the disease. The basic mechanisms of resistance can be categorized into two types: Bcr-Abl-dependent and Bcr-Abl-independent mechanisms. The latter consist mainly of increased drug efflux/decreased uptake and activation of alternative onco-pathways. Bcr-Abl-dependent mechanisms primarily result from the acquisition of point mutations in the *BCR:ABL* gene, although other rarer mechanisms occur, such as *BCR:ABL* gene amplification and hyperexpression, or mutations in other cancer-related genes [5,7]. *BCR:ABL* gene mutations affect the binding of TKIs to different segments of the tertiary structure, such as the phosphate-binding loop (P-loop), the ATP-binding cleft, or the activation loop (A-loop). The most prevalent mutation is T315I, which prevents the correct binding of the TKI to the protein and impairs the activity of imatinib and most second-generation TKIs. Although more than 90 different mutations have been described, the negative impact on the clinical outcome of P-loop mutations, including G250E, Y253H, and E255K/V, has been widely demonstrated [3,8].

**Figure 2:**
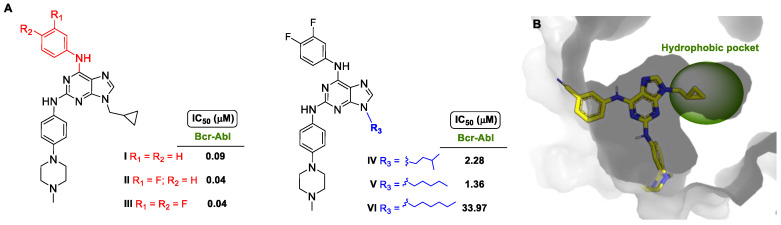
Purine derivatives developed previously by our group as Bcr-Abl inhibitors: (**A**) Chemical structures and their IC_50_ values. (**B**) Predicted binding mode of purines substituted with the cyclopropylmethyl group at *N*-9 to Bcr-Abl [13].

Although the significant advances achieved with drugs such as dasatinib, imatinib, and nilotinib, resistance to these agents remains a clinical challenge. For this reason, Bcr-Abl remains a highly attractive target for the development of potent and selective inhibitors that will represent an important new class of therapeutic agents for the treatment of leukemia [5]. Several new therapies for leukemia are currently in development, and some are under preclinical investigation, most of which are ATP-competitive Bcr-Abl TKIs overriding the T315I mutation, but there are very few reports evaluating the same molecule on different mutations [9,10]. Since the most potent inhibitors of Bcr-Abl are those that bind directly to the ATP binding site, the purine core was chosen as the basis for designing the compounds in this study. This heterocycle has been used as a privileged scaffold in the development of new Bcr-Abl inhibitors. Azam and collaborators performed different substitutions of the 3-hydroxyphenylethyl group at *N*-9 and discovered that the compound AP23846 can bind within the active site of both Bcr-Abl^WT^ and Bcr-Abl^T315I^ [11]. In this context, in 2020, our working group identified promising 2,6,9-trisubstituted purine derivatives **I**–**III** (Figure 2A), which showed enhanced inhibition of Bcr-Abl with an IC_50_ of 0.040–0.090 μM in an Abl kinase inhibition assay [12]. In addition, compound **III** was more selective to Bcr-Abl than other tyrosine kinases (BTK) and a serine/threonine kinase (CDK-2). In addition, **III** exhibited low micromolar cytotoxicity on several leukemia cancer cell lines and decreased the phosphorylation of downstream proteins in the signaling pathways of Bcr-Abl [12]. Later, in 2022, our group demonstrated that the optimal substituent at *N*-9 is the cyclopropylmethyl group of **I**–**III**, as evidenced in the high IC_50_ values for compounds **IV**–**VI** bearing longer hydrophobic substitutions (Figure 2A) and confirmed by molecular docking, which is attributed to the size of the hydrophobic pocket in Bcr-Abl (Figure 2B) [13].

Therefore, according to these antecedents, in this work, we designed and synthesized a series of new 2,6,9-trisubstituted purine derivatives. An inhibition study was then carried out on Bcr-Abl^WT^, followed by a rigorous cytotoxic analysis on some leukemia-related TKI-sensitive and TKI-resistant cancer cell lines that specifically contain T315I, E255K, or Y253H mutated Bcr-Abl. Finally, in silico studies were performed to understand the structure–activity relationship of these ligands on Bcr-Abl^WT^ and Bcr-Abl^T315I^.

## 2. Materials and Methods

### 2.1. Chemistry

All reagents and chemicals used in the chemical synthesis of the intermediates and final compounds were purchased from Sigma Aldrich (St. Louis, MO, USA). Intermediates that have been previously published were synthesized according to the reported procedures, as indicated by the respective reference.

The melting points (mp) of all synthesized compounds were measured without correction on a Kofler Thermogerate apparatus (Reichert, Werke A.G., Wien, Vienna, Austria). The ^1^H and ^13^C nuclear magnetic resonance (NMR) spectra of the synthesis intermediates and final compounds were recorded on a BRUKER AVANCE III HD-400 [400 MHz (^1^H) and 100 MHz (^13^C)] and 200 MHz [200 MHz (^1^H) and 50 MHz (^13^C)] spectrometers (Bruker, Karlsruhe, Germany), respectively. The compounds were dissolved in CDCl_3_ or DMSO-*d*_6_ with tetramethylsilane (TMS) as internal standard. In the NMR spectra, the chemical shift in each signal is given in parts per million (ppm) and, where appropriate, the coupling constants (*J*) are given in Hertz (Hz). The multiplicity observed in the ^1^H NMR spectra for each signal is given as s (singlet), d (doublet), t (triplet), and dd (doublet doublet), respectively. High-resolution mass spectra (HRMS) or mass spectra (MS) were measured on a Q-TOF mass spectrometer (Synapt G2-Si, Waters, Milford, MA, USA) equipped with an electrospray ionization (ESI) source. Briefly, the measurement procedure consisted of injecting an acetonitrile solution of the samples directly into the ESI source using a syringe pump at a flow rate of 10 µL/min. The positive mode molecular ions were detected on the Q-TOF mass spectrometer. Reaction monitoring and verification of the purity of the synthesis products after column chromatography was performed by thin-layer chromatography (TLC) using Merck GF-254 type 60 silica gel (Merck, Burlington, VT, USA). The purity of the final compounds for biological assays was determined by TLC and HRMS spectra.

#### 2.1.1. General Procedure for the Synthesis of Intermediates **2a**–**c**

The synthesis of intermediates **2a**–**c** was carried out according to the procedure described above, which consisted of adding to a solution of 2,6-dichloro-9*H*-purine (**1**, 2.0 g, 1.058 mol) the corresponding alkyl halides (1.58 mol) in the presence of K_2_CO_3_ (3.36 mg, 3.174 mol) and dissolved in DMF (20 mL). The mixture was stirred at room temperature for 12 h. The suspension was then filtered and concentrated on a rotary evaporator. This reaction crude was purified by silica gel chromatography using a mixture of EtOAc/hexane (1:1) as mobile phase to give the pure products **2a**–**c**.

The yields of the compounds obtained after purification were for the following:

- 2,6-Dichloro-9-(cyclopropylmethyl)-9*H*-purine, (**2a**), 51%.
- 2,6-Dichloro-9-isopentyl-9*H*-purine, (**2b**), 48%.
- 2,6-Dichloro-9-hexyl-9*H*-purine, (**2c**), 45%.

The analytical data for **2a**–**c** agreed with the literature [12].

#### 2.1.2. General Procedure for the Synthesis of Intermediates **3a**–**i**

The synthesis of intermediates **3a**–**i** was carried out according to the procedure described above, which consisted of adding the corresponding anilines (0.411 mmol) to a solution of **2a**–**c** (0.411 mmol) in the presence of the base *N*,*N*-diisopropylethylamine (DIPEA, 0.15 mL, 0.822 mmol), all dissolved in *n*-butanol (20 mL). This mixture was stirred at 110 °C for 12 h. After cooling to room temperature, the reaction mixture was concentrated on a rotary evaporator. The solid was then purified by silica gel chromatography using a mobile phase mixture of hexane:AcOEt (70:30) to give products **3a**–**i**.

The yields of the compounds obtained after purification were for the following:

- 2-Chloro-9-(cyclopropylmethyl)-*N*-phenyl-9*H*-purin-6-amine (**3a**), 85%.
- 2-Chloro-9-(cyclopropylmethyl)-*N*-(3-fluorophenyl)-9*H*-purin-6-amine (**3b**), 76%.
- 2-Chloro-9-(cyclopropylmethyl)-*N*-(3,4-difluorophenyl)-9*H*-purin-6-amine (**3c**), 77%.

The analytical data for **3a**–**c** are in accordance with the literature [12].

- 2-Chloro-9-isopentyl-*N*-phenyl-9*H*-purin-6-amine, (**3d**), 88%.
- 2-Chloro-*N*-(3-fluorophenyl)-9-isopentyl-9*H*-purin-6-amine (**3e**), 50%.
- 2-Chloro-*N*-(3,4-difluorophenyl)-9-isopentyl-9*H*-purin-6-amine (**3f**), 67%.
- 2-Chloro-9-hexyl-*N*-phenyl-9*H*-purin-6-amine (**3g**), 71%.
- 2-Chloro-*N*-(3-fluorophenyl)-9-hexyl-9*H*-purin-6-amine (**3h**), 78%.
- 2-Chloro-*N*-(3,4-difluorophenyl)-9-hexyl-9*H*-purin-6-amine (**3i**), 82%.

The analytical data for **3d**–**i** are in accordance with the literature [13].

#### 2.1.3. General Procedures for the Synthesis of Intermediates **5** and **9a**–**b**

To a solution of 1-(chloromethyl)-4-nitrobenzene **4** or 4-fluoro-nitrobenzene **8** (7.1 mmol), 1-methylpiperazine or 2-hidroxyethyl piperazine (7.1 mmol), and K_2_CO_3_ (2226 mg, 21 mmol) in DMF (20 mL) were added, and the mixture was stirred at room temperature for 6 h. Then, the reaction mixture was filtered and evaporated. The mixture was extracted with EtOAc, and the organic layer was dried by anhydrous Na_2_SO_4_ and concentrated to give products **5** and **9a**–**b**.

1-Methyl-4-(4-nitrobenzyl)piperazine, (**5**): Red solid, yield 98%, mp 103–104 °C. ^1^H NMR (400 MHz, CDCl_3_) δ 8.12 (d, *J* = 8.7 Hz, 2H, 2 CH), 7.47 (d, *J* = 8.7 Hz, 2H, 2 CH), 3.56 (s, 2H, CH_2_), 2.46 (m, 8H, 4 CH_2_), 2.29 (s, 3H, CH_3_). ^13^C NMR (101 MHz, CDCl_3_) *δ* 147.17, 146.34, 129.46 (2C), 123.51 (2C), 62.04, 55.01 (2C), 53.00 (2C), 45.89. ESI/MS for (C_12_H_17_N_3_O [M+H]^+^). Calcd: 235.1. Found: 235.1.

1-Methyl-4-(4-nitrophenyl)piperazine, (**9a**): Orange solid, yield 98%. The analytical data corresponded to the literature [13].

2-(4-(4-Nitrophenyl)piperazin-1-yl)ethan-1-ol, (**9b**): Yellow solid, yield 97%, mp 108.0–110.1 °C. ^1^H NMR (200 MHz, CDCl_3_) δ 8.19–8.02 (m, 2H, 2 CH), 6.88–6.73 (m, 2H, 2 CH), 3.68 (t, *J* = 5.3 Hz, 2H, CH_2_), 3.51–3.37 (m, 4H, 2 CH_2_), 2.64 (dt, *J* = 10.5, 5.3 Hz, 6H, 3 CH_2_). ^13^C NMR (50 MHz, CDCl_3_) δ 125.90 (2C), 112.70 (2C), 59.35, 57.89, 52.40 (2C), 47.07 (2C). ESI/MS for (C_11_H_15_N_3_O_2_ [M+H]^+^). Calcd: 251.1. Found: 251.1.

#### 2.1.4. General Procedures for the Synthesis Intermediates **6** and **10a**–**b**

To a solution of **5** or **9a**–**b** (1.35 mmol), Pd-C (30 mg) in ethanol (20 mL) with hydrogen atmosphere was added, and the mixture was stirred at room temperature for 4–6 h. After the completion of the reaction, the reaction mixture was filtered in celite and concentrated to give **6** and **10a**–**b**.

4-((4-Methylpiperazin-1-yl)methyl)aniline, (**6**): Brown solid, yield 98%, mp 122–124 °C. ^1^H NMR (400 MHz, CDCl_3_) δ 7.02 (d, *J* = 7.2 Hz, 2H, 2 CH), 6.56 (d, *J* = 6.6 Hz, 2H, 2 CH), 3.55 (NH_2_, 2H), 3.33 (s, 2H, CH_2_), 2.39 (m, 8H, 4 CH_2_), 2.21 (s, 3H, CH_3_). ESI/MS for (C_12_H_19_N_3_ [M+H]+). Calcd: 206.2. Found: 206.2.

4-(4-Methylpiperazin-1-yl)aniline, (**10a**): Purple solid, yield 99% The analytical data corresponded to the literature [13].

2-(4-(4-Aminophenyl)piperazin-1-yl)ethan-1-ol (**10b**): Purple solid, yield 98%, mp 129.3–133.5 °C. ^1^H NMR (200 MHz, CDCl_3_) δ 6.90–6.74 (m, 2H, 2 CH), 6.74–6.57 (m, 2H, 2 CH), 3.74–3.59 (m, 2H, CH_2_), 3.14–3.00 (m, 4H, 2 CH_2_), 2.75–2.54 (m, 6H, 3 CH_2_). ^13^C NMR (50 MHz, CDCl_3_) δ 118.58 (2C), 116.20 (2C), 59.30, 57.76, 53.06 (2C), 50.96 (2C). ESI/MS for (C_11_H_17_N_3_ [M+H]^+^). Calcd: 221.1. Found: 221.1.

#### 2.1.5. General Procedure for the Synthesis of Intermediates **12a**–**c**

To a solution of **2a** (100 mg, 0.411 mmol) with a solution 25% of ammonium hydroxide (154 mg, 0.411 mmol) in dioxane (20 mL) for **12a**, or benzylamine/cyclohexylamine (0.411 mmol) and DIPEA (0.15 mL, 0.822 mmol) in *n*-butanol (20 mL) for **12b**–**c**, were added and these mixtures were stirred at 100 °C for 12 h. After cooling to room temperature, the reaction mixtures were concentrated on a rotary evaporator. The respective solid was then purified by silica gel chromatography using a mobile phase mixture of acetone/dichloromethane (1:1) to give product **12a**, and a mobile phase mixture of acetone/dichloromethane (1:1) to give product **12b**–**c**.

2-Chloro-9-(cyclopropylmethyl)-9*H*-purin-6-amine, (**12a**): White solid, yield 90%, mp 110–111 °C. ^1^H NMR (200 MHz, DMSO-*d*_6_) δ 8.19 (s, 1H, NH), 7.72 (s, 2H, NH_2_), 3.95 (d, *J* = 7.2 Hz, 2H, CH_2_), 1.27 (ddd, *J* = 15.0, 7.2, 4.5 Hz, 1H, CH), 0.59–0.46 (m, 2H, CH_2_), 0.46–0.36 (m, 2H, CH_2_).^13^C NMR (50 MHz, DMSO-*d*_6_) δ 156.73, 152.89, 150.54, 141.18, 117.72, 47.45, 11.19, 3.71 (2C). ESI/MS for (C_9_H_16_ClN_5_ [M+H]^+^). Calcd: 223.06. Found: 223.10.

2-Chloro-*N*-cyclohexyl-9-(cyclopropylmethyl)-9*H*-purin-6-amine (**12b**): White solid, yield 80%, mp 79–80 °C. ^1^H NMR (200 MHz, CDCl_3_) δ 7.71 (s, 1H, CH), 6.12 (s, 1H, NH), 4.03 (s, 1H, CH), 3.85 (d, *J* = 7.2 Hz, 2H, CH_2_), 1.91 (d, *J* = 11.7 Hz, 2H, CH_2_), 1.54 (ddd, *J* = 32.2, 11.4, 6.8 Hz, 3H, 3CH), 1.40–0.93 (m, 6H, 6 CH), 0.62–0.44 (m, 2H, CH_2_), 0.28 (q, *J* = 4.9 Hz, 2H, CH_2_). ^13^C NMR (50 MHz, CDCl_3_) δ 154.38, 149.87, 139.25, 138.04, 118.11, 48.18 (2C), 32.88, 25.31 (2C), 24.52 (2C), 10.93, 4.06 (2C). ESI/MS for (C_15_H_20_ClN_5_ [M+H]^+^). Calcd: 305.14. Found: 305.15.

*N*-benzyl-2-chloro-9-(cyclopropylmethyl)-9*H*-purin-6-amine (**12c**): White solid, yield 90%, mp 100–102 °C. ^1^H NMR (200 MHz, CDCl_3_) δ 7.63 (s, 1H, NH), 7.32 (dt, *J* = 8.3, 4.7 Hz, 6H, 6CH), 4.85 (s, 2H, CH_2_), 3.97 (d, *J* = 7.2 Hz, 2H, CH_2_), 1.46–1.09 (m, 1H, CH), 0.76–0.59 (m, 2H, CH_2_), 0.41 (q, *J* = 4.9 Hz, 2H, CH_2_). ^13^C NMR (50 MHz, CDCl_3_) δ 155.28, 154.60, 139.86, 138.18, 137.57, 128.80 (2C), 128.02, 127.73 (2C), 118.54, 77.16, 48.62, 11.17, 4.42 (2C). ESI/MS for (C_16_H_16_ClN_5_ [M+H]^+^). Calcd: 313.10 Found: 314.12.

#### 2.1.6. General Procedures for the Synthesis of Final Compounds for Biological Assays **7a**–**b**, **11a**–**i**, and **13a**–**c**

A solution consisting of **3a**–**i** or **12a**–**c** (0.519 mmol) and **6** or **10a**–**b** (0.57 mmol) in dioxane (2 mL), Pd(OAc)_2_ (24 mg, 0.1 mmol), Xanthphos (120 mg, 0. 2 mmol) and 2 M K_2_CO_3_ (aq) (1 mL, 0.2 mmol) were added. These reaction mixtures were stirred at 100 °C for 12–24 h. After cooling to room temperature, these mixtures were filtered on celite, and the filtrates were diluted with dichloromethane. These residues were purified by silica gel chromatography using a methanol/dichloromethane (1:9) mobile phase to give the respective final product. The Rf values of each compound were calculated using the above mobile phase.

9-(Cyclopropylmethyl)-*N*^6^-(3-fluorophenyl)-*N*^2^-(4-((4-methylpiperazin-1-yl)methyl)phenyl)-9*H*-purine-2,6-diamine (**7a**): White solid, yield 68%, mp 132–133 °C, Rf = 0.4. ^1^H NMR (400 MHz, CDCl_3_) δ 8.23 (s, 1H, NH), 7.79 (dt, *J* = 11.4, 1.9 Hz, 1H, CH), 7.66 (s, 1H, CH), 7.57 (d, *J* = 8.4 Hz, 2H, 2CH), 7.28 (s, 1H, CH), 7.24–7.20 (m, 3H, 2CH, NH), 7.15 (dd, *J* = 14.5, 8.0 Hz, 1H, CH), 6.67 (td, *J* = 8.2, 1.6 Hz, 1H, CH), 3.89 (d, *J* = 7.2 Hz, 2H, CH_2_), 3.44 (s, 2H, CH_2_), 2.46 (s, 8H, 4CH_2_), 2.25 (s, 3H, CH_3_), 1.26 (dtd, *J* = 10.4, 7.5, 2.7 Hz, 1H, CH), 0.61 (q, *J* = 5.8 Hz, 2H, CH_2_), 0.39 (q, *J* = 5.0 Hz, 2H, CH_2_). ^13^C NMR (101 MHz, CDCl_3_) δ 163.07 (d, ^1^*J_C-F_* = 243.5 Hz, CF), 156.35, 151.96, 151.32, 140.80 (d, ^3^*J_C-F_* = 11.2 Hz, CH), 139.33, 138.26, 131.35, 129.88 (2C), 129.79 (d, ^3^*J_C-F_* = 9.7 Hz, CH), 119.10 (2C), 115.49, 115.36 (d, ^4^*J_C-F_* = 2.4 Hz, CH), 109.48 (d, ^2^*J_C-F_* = 21.5 Hz, CH), 107.51 (d, ^2^*J_C-F_* = 26.6 Hz, CH), 62.63, 55.05 (2C), 52.84 (2C), 48.28, 45.91, 11.10, 4.37 (2C). ^19^F NMR (376 MHz, CDCl_3_) δ −111.96 (s, 1F). ESI/MS for (C_27_H_31_FN_8_ [M+H]^+^): Calcd: 487.2738. Found: 487.2733.

9-(Cyclopropylmethyl)-*N*^6^-(3,4-difluorophenyl)-*N*^2^-(4-((4-methylpiperazin-1-yl)methyl)phenyl)-9*H*-purine-2,6-diamine (**7b**): Brown solid, yield 64%, mp 122–126 °C, Rf = 0.3. ^1^H NMR (400 MHz, CDCl_3_) δ 8.52 (s, 1H, NH), 7.85 (ddd, *J* = 12.7, 7.2, 2.5 Hz, 1H, CH), 7.66 (s, 1H, CH), 7.56 (d, *J* = 8.3 Hz, 3H, 2CH, NH), 7.22 (d, *J* = 8.3 Hz, 2H, 2CH), 7.13–7.06 (m, 1H, CH), 6.93 (dd, *J* = 18.4, 9.3 Hz, 1H, CH), 3.89 (d, *J* = 7.1 Hz, 2H, CH_2_), 3.44 (s, 2H, CH_2_), 2.46 (s, 8H, 4CH_2_), 2.25 (s, 3H, CH_3_), 1.31–1.18 (m, 1H, CH), 0.67–0.56 (m, 2H, CH_2_), 0.42–0.33 (m, 2H, CH_2_). ^13^C NMR (101 MHz, CDCl_3_) δ 156.32, 151.81, 151.26, 149.83 (dd, ^1,2^*J_C-F_* = 245.6, 13.1 Hz, CF), 146.02 (dd, ^1,2^*J_C-F_* = 243.6, 12.9 Hz, CF), 139.25, 138.16, 135.77 (dd, ^3,4^*J_C-F_* = 9.1, 2.9 Hz, CH), 131.38, 129.76 (2C), 119.13 (2C), 116.73 (d, ^2^*J_C-F_* = 18.1 Hz, CH), 115.67–115.55 (m, CH), 115.15, 109.73 (d, ^2^*J_C-F_* = 21.8 Hz, CH), 62.53, 54.97 (2C), 52.78 (2C), 48.18, 45.84, 10.98, 4.28 (2C). ^19^F NMR (376 MHz, CDCl_3_) δ −136.48 (d, ^3^*J_F-F_* = 22.0 Hz, 1F), −144.88 (d, ^3^*J_F-F_* = 22.1 Hz, 1F). ESI/MS for (C_27_H_31_F_2_N_8_ [M+H]^+^): Calcd: 505.2634. Found: 505.2632.

2-(4-(4-((9-(Cyclopropylmethyl)-6-(phenylamino)-9*H*-purin-2-yl)amino)phenyl)piperazin-1-yl)ethan-1-ol (**11a**): Brown solid, yield 61%, mp 98.2–100.5 °C, Rf = 0.3. ^1^H NMR (200 MHz, DMSO-*d*_6_) δ 9.49 (s, 1H, NH), 8.85 (s, 1H, NH), 8.00 (t, *J* = 4.0 Hz, 4H, 4 CH), 7.65 (d, *J* = 8.7 Hz, 2H, 2 CH), 7.28 (d, *J* = 5.9 Hz, 2H, 2 CH), 7.02 (d, *J* = 7.3 Hz, 1H, CH), 6.86 (d, *J* = 8.8 Hz, 2H, 2 CH), 3.94 (t, *J* = 6.2 Hz, 2H, CH_2_), 3.57 (t, *J* = 6.1 Hz, 2H, CH_2_), 3.22–3.02 (m, 4H, 2 CH_2_), 2.72–2.49 (m, 6H, 3 CH_2_), 1.32 (ddt, *J* = 10.9, 8.0, 3.8 Hz, 1H, CH_2_), 0.50 (ddt, *J* = 13.8, 4.7, 2.7 Hz, 4H, 2 CH_2_). ^13^C NMR (50 MHz, DMSO-*d*_6_) δ 156.20, 151.91, 151.15, 145.51, 140.05, 138.59, 133.72, 128.24 (2C), 121.98, 120.49 (2C), 120.11, 115.87 (2C), 114.53, 59.99, 58.15, 53.04 (2C), 48.86 (2C), 47.03, 11.30, 3.74 (2C). ESI/MS for (C_27_H_32_N_8_O [M+H]^+^): Calcd: 485.2777. Found: 485.2771.

2-(4-(4-((9-(Cyclopropylmethyl)-6-((3-fluorophenyl)amino)-9*H*-purin-2-yl)amino)phenyl)piperazin-1-yl)ethan-1-ol (**11b**): Yellow solid, yield 53%, mp 118.5–120.2 °C, Rf = 0.3. ^1^H NMR (200 MHz, CDCl_3_) δ 7.95 (s, 1H, NH), 7.94–7.83 (m, 1H, NH), 7.71 (s, 1H, CH), 7.55 (d, *J* = 8.9 Hz, 2H, CH), 7.35–7.17 (m, 2H, 2 CH), 7.04–6.85 (m, 3H, 3 CH), 6.74 (ddd, *J* = 9.2, 5.1, 2.6 Hz, 1H, CH), 3.95 (d, *J* = 7.1 Hz, 2H, CH_2_), 3.70 (t, *J* = 5.4 Hz, 2H, CH_2_), 3.19 (dd, *J* = 6.3, 3.6 Hz, 4H, 2 CH_2_), 2.78–2.68 (m, 4H, 2 CH_2_), 2.64 (t, *J* = 5.4 Hz, 2H, CH_2_), 1.45–1.22 (m, 2H, CH_2_), 0.78–0.59 (m, 2H, CH_2_), 0.54–0.37 (m, 2H, CH_2_). ^13^C NMR (50 MHz, CDCl_3_) δ 163.07 (d, ^1^*J_C-F_* = 243.6 Hz), 156.82, 151.82, 151.43, 146.95, 140.77 (d, ^3^*J_C-F_* = 11.4 Hz), 137.97, 132.99, 129.72 (d, ^3^*J_C-F_* = 9.5 Hz), 121.30 (2C), 117.03 (2C), 115.14 (d, ^2^*J_C-F_* = 15.6 Hz), 109.08, 107.32 (d, ^2^*J_C-F_* = 26.7 Hz), 59.38, 57.83, 53.00 (2C), 50.08 (2C), 48.13, 11.05, 4.29 (2C). ^19^F NMR (188 MHz, CDCl_3_) δ −111.74. ESI/MS for (C_27_H_31_FN_8_O [M+H]^+^): Calcd: 503.2683. Found: 503.2682

2-(4-(4-((9-(Cyclopropylmethyl)-6-((3,4-difluorophenyl)amino)-9*H*-purin-2-yl)amino)phenyl)piperazin-1-yl)ethan-1-ol (**11c**): Brown solid, yield 36%, mp 98.1–101.0 °C, Rf = 0.3. ^1^H NMR (400 MHz, CDCl_3_) δ 7.98 (ddd, *J* = 12.9, 7.2, 2.6 Hz, 1H, CH), 7.91 (s, 1H, NH), 7.66 (s, 1H, CH), 7.54–7.45 (m, 2H, 2 CH), 7.17–7.08 (m, 1H, CH), 7.03 (dt, *J* = 10.1, 8.8 Hz, 1H, CH), 6.96–6.87 (m, 2H, CH), 3.91 (d, *J* = 7.2 Hz, 2H, CH_2_), 3.69–3.61 (m, 2H, CH_2_), 3.19–3.12 (m, 4H, 2 CH_2_), 2.71–2.63 (m, 4H, 2 CH_2_), 2.60 (t, *J* = 5.4 Hz, 2H, CH_2_), 1.29 (ddd, *J* = 7.5, 6.1, 3.8 Hz, 1H, CH), 0.64 (q, *J* = 6.0 Hz, 2H, CH_2_), 0.42 (q, *J* = 4.8 Hz, 2H, CH_2_). ^13^C NMR (101 MHz, CDCl_3_) δ 158.33, 156.86, 151.73, 151.46, 148.84, 147.06, 138.00, 135.79 (dd, ^3^*J_C-F_* = 9.3, 2.9 Hz), 132.89, 121.46, 117.00 (2C), 116.81, 115.23 (dd, ^3^*J_C-F_* = 5.4, 3.6 Hz), 115.09, 109.62 (d, ^2^*J_C-F_* = 22.4 Hz), 59.39, 57.83, 53.00 (2C), 50.04 (2C), 48.16, 11.06, 4.30 (2C). ^19^F NMR (376 MHz, CDCl_3_) δ −136.13 (d, ^3^*J_F-F_* = 22.1 Hz), -145.01 (d, ^3^*J_F-F_* = 22.0 Hz). ESI/MS for (C_27_H_30_F_2_N_8_O [M+H]^+^): Calcd: 521.2589. Found: 521.25991.

2-(4-(4-((9-Isopentyl-6-(phenylamino)-9*H*-purin-2-yl)amino)phenyl)piperazin-1-yl)ethan-1-ol (**11d**): Orange solid, yield 51%, mp 88.6–89.2 °C, Rf = 0.3. ^1^H NMR (400 MHz, CDCl_3_) δ 7.81 (s, 1H, NH), 7.67 (d, *J* = 8.0 Hz, 2H, 2 CH), 7.50 (d, *J* = 9.1 Hz, 2H, CH, NH), 7.23 (t, *J* = 7.8 Hz, 2H, 2 CH), 7.02–6.94 (m, 2H, 2 CH), 6.83 (d, *J* = 8.8 Hz, 2H, 2 CH), 4.02 (t, *J* = 7.5 Hz, 2H, CH_2_), 3.62 (t, *J* = 5.4 Hz, 2H, CH_2_), 3.09 (t, *J* = 5.0 Hz, 4H, 2 CH_2_), 2.63 (t, *J* = 4.9 Hz, 4H, 2 CH_2_), 2.56 (t, *J* = 5.4 Hz, 2H, CH_2_), 1.71 (q, *J* = 7.2 Hz, 2H, CH_2_), 1.55 (dp, *J* = 13.3, 6.7 Hz, 1H, CH), 0.91 (d, *J* = 6.6 Hz, 6H. 2 CH_3_). ^13^C NMR (101 MHz, CDCl_3_) δ 156.68, 152.20, 151.26, 146.56, 139.12, 137.97, 133.56, 128.80 (2C), 123.00, 120.71, 120.27 (2C), 117.06 (2C), 115.35, 77.39, 77.07, 76.75, 59.50, 57.85, 53.05 (2C), 50.15 (2C), 41.88, 38.76, 25.62, 22.36 (2C). ESI/MS for (C_28_H_36_N_8_O [M+H]^+^): Calcd: 501.2589. Found: 501.3082.

2-(4-(4-((6-((3-Fluorophenyl)amino)-9-isopentyl-9*H*-purin-2-yl)amino)phenyl)piperazin-1-yl)ethan-1-ol (**11e**): Brown solid, yield 20%, mp 77.2–79.2 °C, Rf = 0.4. ^1^H NMR (200 MHz, CDCl_3_) δ 8.22 (s, 1H, NH), 7.80 (dt, *J* = 11.7, 2.1 Hz, 1H, CH), 7.56–7.39 (m, 3H, NH y 2 CH), 7.23–7.00 (m, 3H, 3 CH), 6.83 (d, *J* = 9.0 Hz, 2H, 2 CH), 6.75–6.53 (m, 1H, CH), 4.00 (t, *J* = 7.4 Hz, 2H, CH_2_), 3.62 (t, *J* = 5.4 Hz, 2H, CH_2_), 3.52 (s, 1H, OH), 3.15–3.01 (m, 4H, 2 CH_2_), 2.68–2.47 (m, 6H, 3 CH_2_), 1.79–1.61 (m, 2H, CH_2_), 1.51 (dq, *J* = 13.3, 6.5 Hz, 1H, CH), 0.89 (d, *J* = 6.4 Hz, 6H, 2 CH_3_). ^13^C NMR (50 MHz, CDCl_3_) δ 165.44, 160.60, 156.77, 151.86, 151.40, 146.81, 140.90 (d, ^3^*J_C-F_* = 11.3 Hz), 138.09, 133.18, 129.65 (d, ^3^*J_C-F_* = 9.6 Hz), 121.16 (2C), 117.04 (2C), 115.10 (d, ^4^*J_C-F_* = 2.4 Hz), 109.20 (d, ^2^*J_C-F_* = 21.6 Hz), 107.33 (d, ^2^*J_C-F_* = 26.8 Hz), 59.57, 57.90, 53.05 (2C), 50.01 (2C), 41.88, 38.68, 25.61, 22.32 (2C). ^19^F NMR (188 MHz, CDCl_3_) δ −111.83. ESI/MS for (C_28_H_35_FN_8_O [M+H]^+^): Calcd: 519.2996. Found: 519.2990.

2-(4-(4-((6-((3,4-Difluorophenyl)amino)-9-isopentyl-9*H*-purin-2-yl)amino)phenyl)piperazin-1-yl)ethan-1-ol (**11f**): Brown solid, yield 48%, mp 110.5–110.9 °C, Rf = 0.4. ^1^H NMR (400 MHz, CDCl_3_) δ 8.26–8.17 (m, 1H, CH), 8.12 (s, 1H, NH), 7.81–7.73 (m, 3H, 3 CH), 7.32 (dd, *J* = 23.8, 9.0 Hz, 2H, 2 CH), 7.25–7.12 (m, 3H, 2 CH, NH), 4.33 (t, *J* = 7.5 Hz, 2H, CH_2_), 3.92 (t, *J* = 5.4 Hz, 2H, CH_2_), 3.40 (t, *J* = 4.9 Hz, 4H, 2 CH_2_), 3.26 (s, 1H, OH), 2.93 (t, *J* = 4.9 Hz, 4H, 2 CH_2_), 2.86 (t, *J* = 5.4 Hz, 2H, CH_2_), 2.01 (q, *J* = 7.2 Hz, 2H, CH_2_), 1.85 (dp, *J* = 13.3, 6.6 Hz, 1H, CH), 1.21 (d, *J* = 6.6 Hz, 6H, 2 CH_3_). ^13^C NMR (101 MHz, CDCl_3_) δ 156.75, 151.73, 151.47, 149.98 (dd, ^3^*J_C-F_* = 12.7, 11.8 Hz), 146.96, 146.08 (dd, ^1^*J_C-F_* = 243.5, 12.9 Hz), 138.27, 135.76 (dd, ^3^*J_C-F_* = 9.2, 2.8 Hz), 133.01, 121.29 (2C), 117.02 (3C), 116.80, 115.41–115.08 (m), 109.63 (d, ^2^*J_C-F_* = 22.2 Hz), 59.43, 57.82, 53.01 (2C), 50.03 (2C), 41.92, 38.72, 25.63, 22.34 (2C). ^19^F NMR (376 MHz, CDCl_3_) δ −136.19 (d, ^3^*J_F-F_* = 22.1 Hz), -144.91 (d, ^3^*J_F-F_* = 22.1 Hz). ESI/MS for (C_28_H_34_F_2_N_8_O [M+H]^+^): Calcd: 537.2902. Found: 537.2902.

2-(4-(4-((9-Hexyl-6-(phenylamino)-9*H*-purin-2-yl)amino)phenyl)piperazin-1-yl)ethan-1-ol (**11g**): Brown solid, yield 78%, mp 114.2–116.7 °C, Rf = 0.3. ^1^H NMR (400 MHz, CDCl_3_) δ 8.26 (s, 1H, NH), 7.70 (d, *J* = 7.8 Hz, 2H, 2 CH), 7.51 (dd, *J* = 7.3, 4.4 Hz, 2H, 2 CH), 7.23 (t, *J* = 7.5 Hz, 2H, 2 CH), 7.00 (q, *J* = 7.2, 6.8 Hz, 1H, NH), 6.89–6.81 (m, 2H, 2 CH), 3.99 (t, *J* = 6.9 Hz, 2H, CH_2_), 3.69 (t, *J* = 5.4 Hz, 2H, CH_2_), 3.11 (q, *J* = 4.8, 4.4 Hz, 4H, 2 CH_2_), 2.72–2.64 (m, 4H, 2 CH_2_), 2.59 (dd, *J* = 10.5, 5.1 Hz, 2H, CH_2_), 1.81 (dq, *J* = 14.5, 7.3, 6.5 Hz, 2H, CH_2_), 1.27 (d, *J* = 4.8 Hz, 6H, 3 CH_2_), 0.83 (s, 3H, CH_3_). ^13^C NMR (101 MHz, CDCl_3_) δ 152.23, 151.27, 146.45, 138.08, 128.76 (2C), 122.94, 120.78 (2C), 120.39 (2C), 117.05 (2C), 115.21, 59.73, 57.94, 53.12 (2C), 49.96 (2C), 43.60, 31.25, 29.79, 26.36, 22.50, 14.04. ESI/MS for (C_29_H_38_N_8_O [M+H]^+^): Calcd: 5515.3247. Found: 515.3240.

2-(4-(4-((6-((3-Fluorophenyl)amino)-9-hexyl-9*H*-purin-2-yl)amino)phenyl)piperazin-1-yl)ethan-1-ol (**11h**): Yellow solid, yield 75%, mp 120.6–122.5 °C, Rf = 0.3. ^1^H NMR (200 MHz, CDCl_3_) δ 8.42 (s, 1H, NH), 7.88 (d, *J* = 11.1 Hz, 1H, CH), 7.61–7.48 (m, 3H, 3 CH), 7.42 (s, 1H, NH), 7.28–7.06 (m, 2H, 2 CH), 6.92 (d, *J* = 8.9 Hz, 2H, 2 CH), 6.70 (ddd, *J* = 8.8, 5.9, 2.1 Hz, 1H, CH), 4.04 (t, *J* = 7.2 Hz, 2H, CH_2_), 3.95 (s, 1H, OH), 3.80–3.65 (m, 2H, CH_2_), 3.15 (dd, *J* = 6.6, 3.4 Hz, 4H, 2 CH_2_), 2.76–2.64 (m, 4H, 2 CH_2_), 2.61 (d, *J* = 5.4 Hz, 2H, CH_2_), 1.84 (q, *J* = 7.1 Hz, 2H, CH_2_), 1.30 (d, *J* = 4.4 Hz, 6H, 3 CH_2_), 0.96–0.79 (m, 3H, CH_3_). ^13^C NMR (50 MHz, CDCl_3_) δ 163.00 (d, ^1^*J_C-F_* = 243.3 Hz), 156.84, 151.90, 151.47, 146.82, 140.96 (d, ^3^*J_C-F_* = 11.3 Hz), 133.19, 129.60 (d, ^3^*J_C-F_* = 9.5 Hz), 121.21 (2C), 116.99 (2C), 115.20 (d, ^4^*J_C-F_* = 3.7 Hz), 109.14 (d, ^2^*J_C-F_* = 21.3 Hz), 107.37 (d, ^2^*J_C-F_* = 26.7 Hz), 67.06, 59.71, 58.01, 53.12 (2C), 49.97 (2C), 43.59, 31.22, 29.75, 26.34, 22.47, 14.00. ^19^F NMR (188 MHz, DCl_3_) δ −111.82. ESI/MS for (C_29_H_37_FN_8_O [M+H]^+^): Calcd: 5533.3153. Found: 533.3148.

2-(4-(4-((6-((3,4-Difluorophenyl)amino)-9-hexyl-9*H*-purin-2-yl)amino)phenyl)piperazin-1-yl)ethan-1-ol (**11i**): Brown solid, yield 55%, mp 135.1–137.1 °C, Rf = 0.3. ^1^H NMR (200 MHz, DMSO-*d*_6_) δ 9.76 (s, 1H, NH), 8.96 (s, 1H, NH), 8.32 (dd, *J* = 14.7, 7.6 Hz, 1H, CH), 7.96 (s, 1H, CH), 7.73 (d, *J* = 8.9 Hz, 1H, CH), 7.62 (d, *J* = 8.4 Hz, 2H, 2 CH), 7.29 (q, *J* = 9.7 Hz, 1H, CH), 6.87 (d, *J* = 8.5 Hz, 2H, 2 CH), 4.44 (s, 1H, OH), 4.08 (t, *J* = 7.0 Hz, 2H, CH_2_), 3.55 (t, *J* = 6.1 Hz, 2H, CH_2_), 3.18 (s, 2H, CH_2_), 3.06 (d, *J* = 5.9 Hz, 4H, 2 CH_2_), 2.65–2.52 (m, 4H, 2 CH_2_), 1.84 (t, *J* = 7.2 Hz, 2H, CH_2_), 1.28 (s, 6H, 3 CH_2_), 0.85 (d, *J* = 6.6 Hz, 3H, CH_3_). ^13^C NMR (50 MHz, DMSO-*d*_6_) δ 156.08, 151.42 (d, ^3^*J_C-F_* = 7.5 Hz), 151.24 (d, ^1^*J_C-F_* = 191.0 Hz), 145.82, 145.20 (d, ^1^*J_C-F_* = 133.7 Hz), 139.07, 138.63, 137.26 (d, ^3^*J_C-F_* = 7.0 Hz), 133.42, 120.33 (2C), 120.25, 116.82, 116.48, 116.21–116.03 (m), 115.83 (2C), 114.51, 109.02 (d, ^2^*J_C-F_* = 22.2 Hz), 60.21, 58.44, 53.20 (2C), 49.07 (2C), 48.55, 30.64, 29.11, 25.67, 21.92, 13.79. ^19^F NMR (188 MHz, DMSO-*d*_6_) δ −137.59 (d, ^3^*J_F-F_* = 23.4 Hz), -147.06 (d, ^3^*J_F-F_* = 23.4 Hz). ESI/MS for (C_29_H_36_F_2_N_8_O [M+H]^+^): Calcd: 551.3059. Found: 551.3064

9-(Cyclopropylmethyl)-*N*^2^-(4-(4-methylpiperazin-1-yl)phenyl)-9*H*-purine-2,6-diamine (**13a**): White solid, yield 54%, mp 133–134 °C, Rf = 0.3. ^1^H NMR (200 MHz, CDCl_3_) 7.81 (s, 1H, CH), 7.66 (s, 1H, CH), 7.64–7.58 (m, 2H, 2 CH), 7.04 (s, NH_2_), 6.91 (d, *J* = 9.6 Hz, 2H, 2 CH), 3.93 (d, *J* = 7.2 Hz, 2H, CH_2_), 3.80 (s, 3H, CH_3_), 3.19 (t, *J* = 3.6 Hz, 4H, 2 CH_2_), 2.64 (dd, *J* = 5.9, 3.8 Hz, 4H, 2 CH_2_), 1.30 (dd, *J* = 11.0, 6.2 Hz, 1H, CH), 0.72–0.60 (m, 2H, CH_2_), 0.44 (q, *J* = 6.4, 5.7 Hz, 2H, CH_2_). ^13^C NMR (101 MHz, CDCl_3_) δ 156.77, 152.41, 151.12, 146.45, 137.59, 132.12, 122.44 (2C), 115.04 (2C), 114.02, 77.68, 77.05, 76.41, 55.51, 55.12 (2C), 48.07, 45.98, 11.07, 4.26 (2C). ESI/MS for (C_20_H_26_N_8_ [M+H]^+^). Calcd: 353.2197. Found: 353.2656.

*N*^6^-cyclohexyl-9-(cyclopropylmethyl)-*N*^2^-(4-(4-methylpiperazin-1-yl)phenyl)-9*H*-purine-2,6-diamine (**13b**): Black solid, yield 60%, mp 122–123 °C, Rf = 0.3. ^1^H NMR (200 MHz, CDCl_3_) δ 7.64–7.44 (m, 3H, 3CH), 7.07 (s, 1H, NH), 6.88 (d, *J* = 8.9 Hz, 2H, 2 CH), 5.75 (d, *J* = 7.7 Hz, 1H, NH), 4.08 (s, 1H, CH), 3.86 (d, *J* = 7.1 Hz, 2H, CH_2_), 3.23–3.07 (m, 4H, 2 CH_2_), 2.70–2.50 (m, 4H, 2 CH_2_), 2.33 (s, 3H, CH_3_), 2.19–2.01 (m, 2H, 2 CH), 1.88–1.51 (m, 2H, 2 CH), 1.51–1.07 (m, 7H, 7 CH), 0.70–0.50 (m, 2H, CH_2_), 0.38 (q, *J* = 5.1 Hz, 2H, CH_2_). ^13^C NMR (101 MHz, CDCl_3_) δ 156.89, 154.16, 146.07, 136.81, 134.14, 119.90, 119.81 (2C), 117.09 (2C), 114.57, 55.16 (2C), 50.10 (2C), 49.41, 47.90, 46.03, 33.25, 30.88, 25.69, 25.04, 11.10, 4.19, 4.14 (2C). ESI/MS for (C_26_H_36_N_8_ [M+H]^+^). Calcd: 461.3136. Found: 461.3139.

*N*^6^-benzyl-9-(cyclopropylmethyl)-*N*^2^-(4-(4-methylpiperazin-1-yl)phenyl)-9*H*-purine-2,6-diamine (**13c**): White solid, yield 63%, mp 152–154 °C, Rf = 0.4. ^1^H NMR (200 MHz, CDCl_3_) δ 7.51–7.41 (m, 3H, 3 CH), 7.33–7.13 (m, 5H, 5 CH), 6.88–6.75 (m, 3H, 2 CH, NH), 6.25 (s, 1H, NH), 4.73 (d, *J* = 5.5 Hz, 2H, CH_2_), 3.81 (d, *J* = 7.1 Hz, 2H, CH_2_), 3.18–2.90 (m, 4H, 2 CH_2_), 2.58–2.46 (m, 4H, 2 CH_2_), 2.28 (s, 3H, CH_3_), 1.33–1.02 (m, 1H, CH), 0.66–0.47 (m, 2H, CH_2_), 0.34 (q, *J* = 4.9 Hz, 2H, CH_2_). ^13^C NMR (50 MHz, CDCl_3_) δ 156.85, 154.79, 151.19, 146.25, 139.06, 137.18, 133.81, 128.53 (2C), 127.63 (2C), 127.20, 119.98 (2C), 117.05 (2C), 114.82, 55.20 (2C), 50.08 (2C), 47.98, 46.09, 44.59, 11.09, 4.22 (2C). ESI/MS for (C_27_H_32_N_8_ [M+H]^+^). Calcd: 469.2823. Found: 469.2824.

### 2.2. Kinase Assays

Inhibitory activity of prepared compounds was tested on a recombinant Abl1 kinase produced in-house. The kinase was assayed with a substrate 500 µM peptide GGEAIYAAPFKK in the presence of 1 µM ATP + [γ-^33^P]ATP (0.05 µCi per reaction) and the test compound in a final volume of 10 µL of reaction buffer (60 mM HEPES-NaOH, pH 7.5, 3 mM MgCl_2_, 3 mM MnCl_2_, 3 μM Na-orthovanadate, 1.2 mM DTT, 2.5 μg/50 μL PEG_20.000_). The reactions were stopped by adding 5 µL of 3% aq. H_3_PO_4_. Aliquots were spotted onto P-81 phosphocellulose (Whatman, Maidstone, UK), washed 3× with 0.5% aq. H_3_PO_4_, and air-dried. Peptide phosphorylation was quantified using digital autoradiography (FLA-7000, Fujifilm, Tokyo, Japan). The concentration of the test compounds required to reduce the activity by 50% was determined from dose–response curves and reported as the IC_50_ value.

### 2.3. Docking

For the 14 final products proposed as inhibitors of these kinases, their three-dimensional structures were constructed using OECHEM and then the respective protonation states were adjusted at pH 7.2 using FixpKa from the QUACPAC package. Conformers for these ligands were generated using OMEGA v. 4.1.2.0 software. The crystal structures of Bcr-Abl^WT^ and Bcr-Abl^T315I^ kinases were downloaded from the RCSB PDB Protein Data Bank (6BL8 and 4TWP, respectively). These crystals were stripped of any water molecules present, as well as any ions and cofactors present. Hydrogen atoms and protons were then added, and partial charges assigned (according to the protonation state at physiological pH). The kinases were prepared using the Chimera USCF program [14]. A local minimization was then performed to eliminate possible bad contacts. The minimization was carried out in the presence of certain constraints to keep the kinase conformation very similar to that observed in the experimental model. Molecular docking studies were carried out using Glide [15] and docking runs were performed using the standard and extra precision scoring approaches (SP and XP scoring) implemented in the software [16]. The best poses for each ligand were optimized using Prime, and the binding energy was estimated by considering the solvation energies of the interacting molecules in addition to the molecular mechanics (MM) energies. The contribution of polar solvation energies was calculated using the generalized Born implicit solvent (GBI) model, while the non-polar contribution of solvation energy depended on the solvent-accessible area (SA) [17]. The interactions of the active site amino acid residues with the ligands were identified using a 6 Å radius around the docked position as a reference. Images were obtained using PyMol v.2.5.2 software.

### 2.4. Cell Cultures

Human cell lines KCL22 and BV173 were obtained from the German Collection of Microorganisms and Cell Cultures, K562 and HEK-293T from the European Collection of Authenticated Cell Cultures. KBM5 cell line was kindly gifted by Vladimír Divoký. Development of KCL22 subclones B8 (T315I, 100%), F4 (E255K, 100%), and B10 (Y253H, 50%) was reported previously [18]. All cell lines were cultivated in humidified incubator at 37 °C and in 5% CO_2_ according to the provider’s instructions in appropriate media supplemented with 10% fetal bovine serum, penicillin (100 IU/mL), streptomycin (100 µg/mL), and glutamine (4 mM).

### 2.5. Cytotoxicity Assay

Cytotoxicity was determined using resazurin, an indicator dye to measure oxidation-reduction reactions occurring in the mitochondria of live cells. The cells were seeded into 96-well plates and treated with compounds for 72 h (six different doses of each, in triplicate). After treatment, resazurin (Merck, Darmstadt, Germany) solution was added for 4 h, and the fluorescence of formed resorufin corresponding to live cell quantity was measured at 544 nm/590 nm (excitation/emission) using a Fluoroskan Ascent microplate reader (Labsystems, Vantaa, Finland). The GI_50_ value, the drug concentration lethal to 50% of the cells, was calculated from the dose–response curves in Origin 6.0 software.

### 2.6. Flow Cytometry

The cell lines were seeded into 96-well plates and then treated with compounds for 24 h. After treatment, the cells were directly stained by adding 5 × staining solution (17 mM trisodium citrate dihydrate, 0.5% IGEPAL^®^ CA-630, 7.5 mM spermine tetrahydrochloride, 2.5 mM Tris; pH 7.6 containing 50 μg/mL propidium iodide). DNA content was measured by flow cytometry using a 488 nm laser (BD FACS Verse with software BD FACSuite™, version 1.0.6.). Cell cycle distribution was analyzed using ModFit LT 5.0.9 (Verity Software House, Augusta, ME, USA).

### 2.7. Immunoblotting and Antibodies

Cell lysates were separated on SDS–polyacrylamide gels and electroblotted onto nitrocellulose membranes. After blocking, overnight incubation with specific primary antibodies was performed, followed by wash and incubation with peroxidase-conjugated secondary antibodies (Cell Signalling). Peroxidase activity was detected with SuperSignal West Pico reagents (Thermo Scientific, Waltham, MA, USA) using a CCD camera LAS-4000 (Fujifilm, Tokyo, Japan). The following specific primary antibodies were used: Cell Signalling: anti-STAT5 (#94205), anti-p-STAT5 Y694 (#9351), anti-Crkl (#3182), anti-p-Crkl Y207 (#3181), anti-cyclin A (#4656), anti-p-Bcr Y177 (#3901), and anti-PARP (#9532); Santa Cruz Biotechnology: anti-β-actin (#sc-47778).

## 3. Results and Discussion

### 3.1. Design and Synthesis

**Figure 3:**
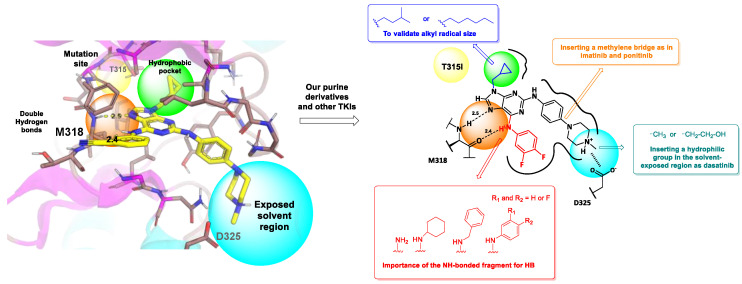
Design of new purine derivatives as potential inhibitors of Bcr-Abl.

In the design of our compounds, we considered our previous results and the chemical structures of the purine-based kinase inhibitors (Figure 3). Firstly, the aminophenyl fragment on C-6 was mainly substituted with one or two fluorine atoms in *meta* and/or *para* positions, like **II** or **III** (Figure 2). In addition, an amino (-NH_2_), cyclohexylamino (-NH-Cy), or benzyl (-NH-Bz) group was substituted on C-6. This modification led us to validate the hypothesis that to inhibit Bcr-Abl, a hydrogen bond in the binding pocket had to be between an aminophenyl fragment and Met318 (Figure 3). Secondly, to confirm the evidence found in our previous work that the optimal hydrophobic moiety on *N*-9 is a cyclopropylmethyl group (**I**–**VI**, Figure 2); modifications in the volume and length of this alkyl group were considered. To complete this design, the *N*-methyl-arylpiperazine fragment on C-2 was conserved in some of these new purine derivatives, and in others, (i) the methyl group was replaced by a hydroxyethyl group mimicking the moiety present in dasatinib, and this portion was oriented towards the exposed solvent region, or (ii) a methylene group was incorporated between the piperazine and aromatic rings to explore the influence of the flexibility of this fragment on Bcr-Abl activity, mimicking imatinib or ponatinib.

**Scheme 1:**
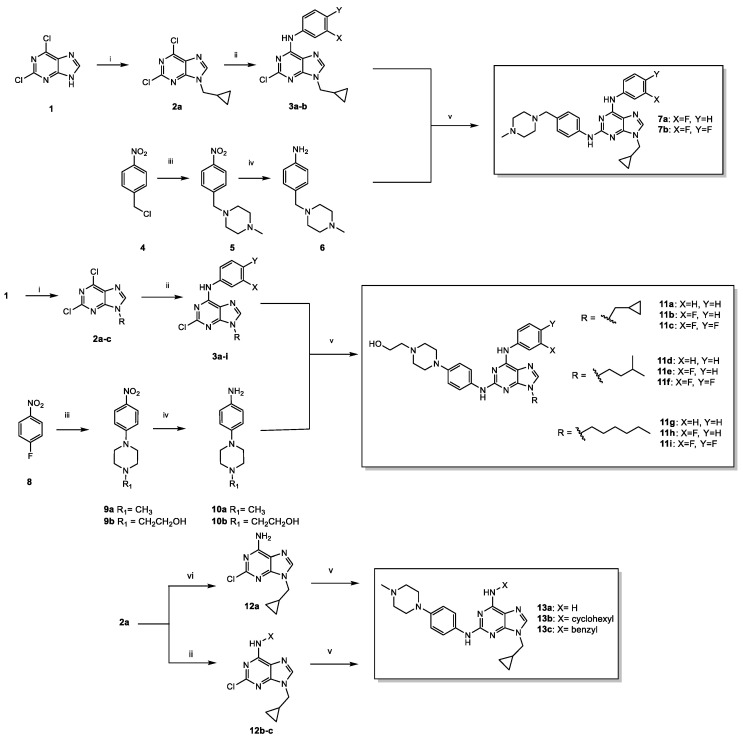
Reagents and conditions: (i) Alkyl halides, K_2_CO_3_, DMF, 12 h, r.t., 45–51%. (ii) Anilines, DIPEA, *n*-butanol, reflux, 12 h, 50–88%. (iii) 4-Methylpiperazine or *N*-(2-hydroxyethyl)piperazine, K_2_CO_3_, DMF, r.t., 6 h, 97–98%. (iv) H_2_, Pd-C, ethanol, r.t., 4–6 h, 98–99%. (v) Piperazinyl anilines, 2 M K_2_CO_3_, Pd(OAc)_2_, Xantphos, dioxane, reflux, 12–24 h, 20–78%. (vi) NH_4_OH, dioxane, 100 °C, 8 h, 90%.

The synthesis of these new 2,6,9-trisubstituted purines **7a**–**b**, **11a**–**i**, and **13a**–**c** was carried out by short, simple, and efficient synthetic methods described by our group and shown in Scheme 1 [12,13,19]. We obtained 14 compounds using 2,6-dichloropurine (**1**) as a starting material. The first step was the alkylation of **1** with the respective alkyl halides under basic conditions to give **2a**–**c** [12,13,19]. To obtain **7a** or **7b**, later, a regioselective nucleophilic substitution (S_N_Ar) at position C-6 with 3-floroaniline or 3,4-difluoroaniline, using *n*-butanol as the solvent and *N*,*N*-diisopropylethylamine as the base, in reflux for 12 h gave compounds **3a**–**b**. Subsequently, a Buchwald–Hartwig C-N coupling reaction at C-2 of **3a**–**b** with 4-((4-methylpiperazin-1-yl)methyl)aniline (**6**) catalyzed by palladium (II) afforded the purine derivatives **7a**–**b** in 67–68% yields. Compound **6** was previously synthesized from 1-(chloromethyl)-4-nitrobenzene (**4**) in two synthetic steps, as shown in Scheme 1. The synthesis of **11a**–**i** considered a similar route to that of **7a**–**b** and our previously reported methodology [12,13], the difference being that in this case, we used *N*-(2-hydroxyethyl)piperazine and 4-fluoro-nitrobenzene (**8**) as starting materials to quantitatively obtain the required aniline derivative **10a**. Finally, the purine derivatives **13a**–**c** were synthesized following the same logic of the previous syntheses but using amines or ammonia in the second step (nucleophilic substitution at C-6, 80–90% yield) and the *N*-methyl-piperazinyl-aniline **10b** for the C-N coupling reaction on C-2 with moderate yields (54–63%). All compounds were purified by column chromatography, and their structures were established based on their spectral properties (^1^H NMR and ^13^C NMR; see the Materials and Methods section and Supplementary Materials).

### 3.2. Kinase Inhibition and Structure–Activity Relationship

In accordance with the goal of this work to discover new Bcr-Abl inhibitors, the next step was to screen all the synthesized compounds for the inhibitory activity on this kinase. Considering the results shown in Table 1, there are some compounds with better activity than the reference drugs, imatinib and nilotinib, such as **11b** and **11c**, which elicited lower IC_50_ values (0.327 and 0.047 μM vs. 0.015 and 0.020 μM, respectively).

On the other hand, from a chemical point of view, a structure–activity relationship (SAR) can be established for this target:

1. The incorporation of a methylene linker between the piperazine and phenylamino ring at C-6 diminished the inhibitory potency on Bcr-Abl in **7a** and **7b** compared to their analogues **II** and **III** (0.180 and 0.225 μM vs. 0.040 μM, respectively). This indicates that the flexibility of this moiety is disadvantageous for the Bcr-Abl activity.
2. The substitution of methyl by a hydroxymethyl group in the *N*-piperazine ring increased the inhibition of Bcr-Abl activity, this behavior being observed in **11a**–**c** compared to **I**–**III**. The IC_50_ values diminished from 0.090 to 0.037 μM (for **I** to **11a**), from 0.045 to 0.015 μM (for **II** to **11b**), and from 0.040 to 0.020 μM (for **III** to **11c**). Interestingly, this fragment is present in dasatinib, and according to our previous docking results, this fragment is in the solvent-exposed region. This effect is so significant that even compounds with more voluminous hydrophobic moieties increased their inhibitory activity (**11f** vs. **IV** and **11i** vs. **VI**).
3. The fact that the optimal substitution for alkyl chains at *N*-9 is by the cyclopropylmethyl group is confirmed, as can be seen by the comparison of compounds **11a**–**c** with their respective analogues **11d**–**i**. This result support the hypothesis about the size of the hydrophobic region in Bcr-Abl compared to other kinases, such as BTK or FLT3 [13].
4. These results also prove that the substitution of hydrogens by fluorine atoms in *meta* and *para* positions of the 6-aminophenyl fragment is beneficial for the inhibition of this kinase.
5. Finally, it is also demonstrated that it must be a 6-aminophenyl fragment and not just a substituent on the purine that possesses the amino group, because according to the IC_50_ values, any option other than the benzene ring drastically decreases the kinase activity (**13a**–**c**, with IC_50_ values between 0.845 μM and 11.06 μM). This would indicate that not only the hydrogen bond donor group is a key to the binding site, but there are also important steric and electronic effects to consider.

### 3.3. Molecular Docking Studies for Bcr-Abl^WT^

Docking calculations were performed to understand the differences in activity of these purine derivatives on Bcr-Abl^WT^. The molecular docking protocol was first validated by performing self-docking of the co-crystallized ligand for the purvalanol B-Bcr-Abl (PDB ID: 6BL8) [20]. The docking protocol showed that the program succeeds in reproducing the main interactions between the co-crystallized ligand and their respective proteins (Figure S1). The complexes with the best docking poses were further energy minimized and the interaction energy was estimated using the MM/GBSA method. The results of the binding energies for all compounds are shown in Table S1. According to these results, a trend was observed in the affinity energies obtained by different calculation methods, such as RDock, XP Score (Glide), and MM-GBSA, and the IC_50_ values on Bcr-Abl^WT^. These computer programs allowed for efficient discrimination between the most and least active compounds. For example, the ΔG binding values were −74.32 and −48.37 kcal/mol for **11c** (IC_50_ = 0.020 μM) and for **13a** (IC_50_ = 3.96 μM). The correlation of the biological activity is shown in Figure S2.

**Figure 4:**
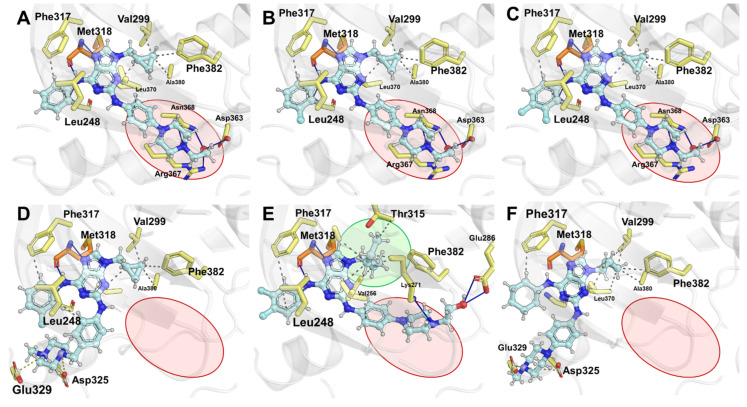
Graphical representation of the poses obtained by molecular docking of the most active compounds, **11a** (**A**), **11b** (**B**), and **11c** (**C**), and the less active compounds, **7a** (**D**), **11i** (**E**), and **13b** (**F**), into the binding site of Bcr-Abl (PDB ID: 6BL8). The red region is occupied when piperazine has a hydroxyl group and the green region is highlighted by Thr315 in the hydrophobic pocket.

In addition, the binding interactions, and the orientation patterns for the most and the least active compounds (**11a**–**c** and **13a**–**b**) are determinants for Bcr-Abl inhibition. As we previously reported [13], a network of multiple interactions was observed between **11a**–**c** and the binding pocket of this kinase (Figure 4A–C), considering a double hydrogen bridge between the *N*-7/NH-phenyl amino group and Met318, which also allowed for a series of hydrophobic interactions with Val299, Ala380, Phe382, and Leu370 in the hydrophobic cavity. However, the increase in the inhibitory activity of **11a**–**c** compared to **I**–**III** could be attributed to the new hydrogen bonds formed between the hydroxymethyl fragment of these purines with Asp363, Asp367, and Asn368 (red color in Figure 4) in the solvent-exposed region. This evidence confirms our hypothesis mentioned in the design section. Similarly, these mentioned interactions could also explain the increase in potency of **11i** (Figure 4E) compared to **VI** (Figure 2).

On the other hand, this docking study can explain the low activity of **7a**–**b** and **13 a**–**c**. For **7a**–**b**, the incorporation of a methylene linker between the piperazine and the phenylamino ring at C-2 prevents the formation of the pattern interactions observed for **11a**–**c** in the red region (Figure 4D). Similarly, for **13a**–**c**, the absence of the phenylamino moiety at C-6 prevents the formation of interactions with Leu248 (Figure 4F) and eventually with Phe317 for **13a** and **13c**, confirming the importance of this fragment as part of the purine pharmacophore.

Finally, according to these in silico results, the optimal size of the of alkyl substitutions at *N*-9 would be a cyclopropylmethyl group instead of *n*-hexyl for Bcr-Abl^WT^. However, an isopentyl group on *N*-9 could establish hydrophobic interactions with T315 (Figure 4E, green region). Likewise, the substitution of hydrogens atoms by fluorine atoms of the aminophenyl ring at C-6 is beneficial for Bcr-Abl inhibition.

### 3.4. Cytotoxic Studies

First, all tested compounds were screened for cytotoxic activity against cell lines with Bcr-Abl rearrangement, such as KBM5 (chronic myeloid leukemia), BV173 (B-cell precursor leukemia), K562 (chronic myelogenous leukemia), as well as against non-neoplastic cells HEK-293T (Table 2). Overall, the cytotoxicity of the 2,6,9-trisubstituted purines was heterogeneous depending on the cell type, as well as the assayed compounds. K562 was the most sensitive cell line (all compounds with GI_50_ values between 0.7 and 6.3 μM, except **13a** with GI_50_ > 100 μM), while KCL22 was the least sensitive (only six compounds with GI_50_ values < 6.5 μM). Among the compounds tested, **11a**–**c** proved to be the most potent, with GI_50_ values not exceeding 3 µM in the Bcr-Abl rearranged cell lines tested. Interestingly, their cytotoxicity was in most cases more pronounced than their template analogues **I**–**III**, indicating the importance of the *N*-hydroxyethyl group in their structure. Likewise, similarly to the Bcr-Abl inhibition, the aminophenyl fragment at C-6 was pivotal for the antiproliferative effect demonstrated by comparing **11a** with **13a**–**c**, which were less cytotoxic in all cell lines. In general, the cytotoxicity of the purine derivatives was consistent with enhanced Abl inhibition, especially in the K562 and KCL22 cell lines, with similar structure–activity relationships to those described above. Compound **11b** was found to be the most potent derivative with GI_50_ values of 1.3, 1.5, and 0.7 μM for KBM5, BV173, and K562 cell lines. However, none of the synthetized compounds showed better performance than TKIs.

To directly compare the sensitivity of synthesized compounds to the most common Bcr-Abl mutations, they were evaluated on a panel of KCL22 subclones expressing different Bcr-Abl point mutants, including B8 (T315I, 100%), F4 (E255K, 100%), and B10 (Y253H, 50%) [18]. The sensitivity to these compounds was evaluated by cytotoxicity assays, and these results were expressed as GI_50_ values (Table 3). The growth of KCL22 cells harboring WT or mutant Bcr-Abl was inhibited at different potencies depending on the purine derivative. In particular, the WT cell line was sensitive to all derivatives with GI_50_ values in the micromolar range except **13a**–**c** and **11c**. From a chemical point of view, this confirms that the aminophenyl fragment on C-6 is pivotal for the antiproliferative effect, which was demonstrated by comparing **13a**–**c** with its respective analogues. Compared to newly synthesized compounds, template molecules **II** and **III** showed only weak antiproliferative effects overall and the incorporation of a methylene linker in **7a** and **7b** did not lead to any major improvements. Among all compounds tested, **11b** and **11c** exhibited the strongest antiproliferative properties on Bcr-Abl^WT^ cells (GI_50_ = 2.0 and 2.9 µM, respectively), consistent with enhanced Abl inhibition. However, none of the synthesized derivatives overcame the potency of the reference drugs, imatinib (GI_50_ = 0.482 μM) and nilotinib (GI_50_ = 0.019 μM). Interestingly, both standards were ineffective in inhibiting the growth of B8 cells carrying the T315I mutation (GI_50_ > 20 µM), whereas compounds **11c**–**f** and **11h** reached single-digit micromolar GI_50_ values. It should be recalled that the T315I mutation is known to be responsible for resistance to nilotinib [21] and imatinib [22], and thus this result agrees with this clinical evidence. Such antiproliferative effect of **11c**–**f** (GI_50_ = 6.4–7.2 µM) suggests that possible inhibition of Bcr-Abl^T315I^ is favored by the presence of an isopentyl group at *N*-9, rather than that observed for Bcr-Abl^WT^, where the most optimal substitution was cyclopropyl group. This challenging hypothesis will be discussed in detail in the next section using in silico studies. In addition, when comparing the GI_50_ values obtained in F4 and B10 mutated cell lines, the synthesized derivatives **11b**–**f** and **I** showed promising antiproliferative effects in both mutated cell lines (GI_50_ = 4.1–5.7 µM and 2.6–7.8 µM, respectively), overcoming the potency of imatinib, which only weakly inhibited the proliferation of the F4 mutant cell line (GI_50_ = 9.6 µM), with B10 cells being resistant (GI_50_ > 20 µM). Consequently, **11b**, as the compound with the best overall potency, and **11e**, with approximately equal antiproliferative activity in the KCL22-derived cell lines tested (including the resistant B8 mutant model), were selected for detailed experiments to clarify their cellular effects.

### 3.5. In Silico Studies for Bcr-Abl ^T315I^

To explain the observed results on the antiproliferative effect of these purines between KCL22 WT and KCL22 B8 cells, we assume that they are related to the presence of Bcr-Abl in its WT or T315I mutant state, as well as to the degree of inhibition of these purines. Therefore, to validate this hypothesis, we carried out molecular docking studies on the Bcr-Abl^T315I^ (PDB ID: 4TWP [23]). Results showed a correlation between the binding affinity energies and the GI_50_ values shown in Table 3 (Figure S3), highlighting the positioning of the most potent antiproliferative compounds, **11b**–**f**, on KCL22 B8 cells (Table 4). According to Table 4, some purine derivatives with isopentyl group at *N*-9 (**11d**–**f**) had higher ΔG binding than those with cyclopropylmethyl group (**11b**–**c**), which could be related to the level of possible inhibition on Bcr-Abl^T315I^ and could indicate the role of this fragment.

**Figure 5:**
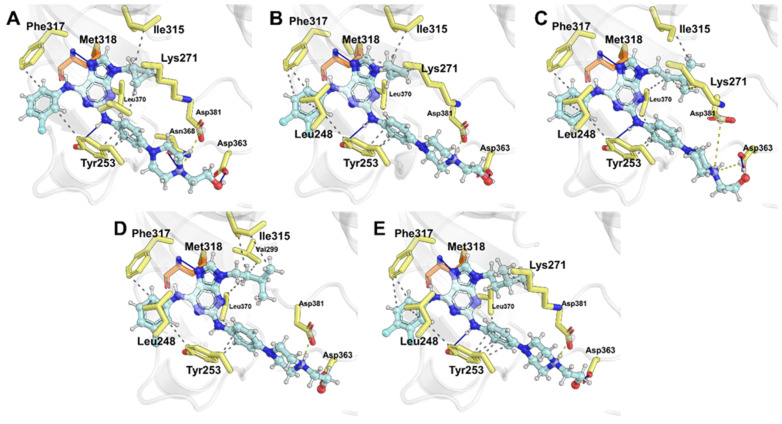
Graphical representation of the poses obtained by molecular docking of the ligands **11b** (**A**), **11c** (**B**), **11d** (**C**), **11e** (**D**), **11f** (**E**) and in the binding site of Bcr-Abl^T315I^.

Comparing the pattern of interactions to stabilize the ligand–Bcr-Abl^T315I^ complex with the ligand–Bcr-Abl^WT^ complex, it is observed that some of them are conserved in both models, such as the essential hydrogen bonds with Met318 with the purine ring and Asp363 with the hydroxyethyl group (Figure 5A–E). However, some new interactions and conformations were observed in Bcr-Abl^T315I^. In the hydrophobic pocket, **11c**–**e** showed interactions with the residues Ile315, Ala269, and Lys271. Interestingly, Ile315 is the point of mutation and Ala269 and Lys271 are residues present in the deepest hydrophobic pocket and contribute significantly to the improved affinity energy, as shown in Table 4 (ΔG Lipo). Moreover, a new hydrogen bonding interaction between the aminophenyl group at C-2 of the purine ring with Tyr253 was observed for **11b**–**d** and **11f**, and an electrostatic interaction between the protonated piperazine fragment moiety and Asp381 was also observed for these purines, the latter being interactions that strongly contribute to ligand–kinase stabilization (Table 4, ΔG Coul.). On the other hand, the eventual most active compounds against Bcr-Abl^T315I^, **11e** and **11f**, showed interaction profiles similar to that of compound **11c** (Figure 5B,D,E). However, the incorporation of one or two fluorine atoms in the phenyl of these compounds enhances the hydrophobic interactions with Phe317 and Leu248. This effect is clearly reflected in the calculated van der Waals energies values (Table 4, ΔGvdW).

**Figure 6:**
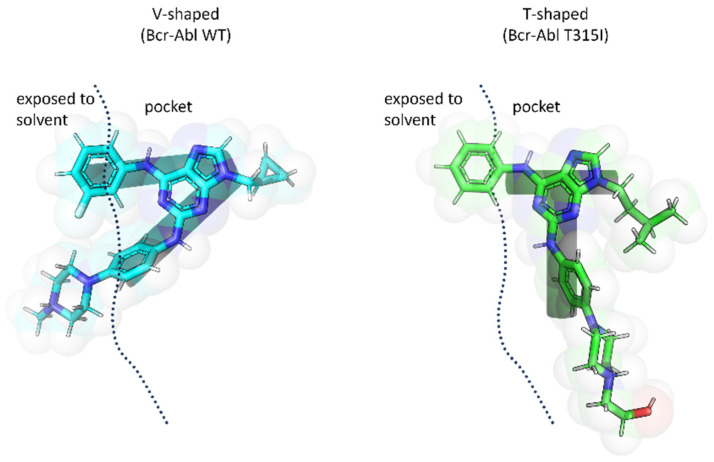
Proposed conformations of **II** at the binding site of Bcr-Abl^WT^ and of **11e** in Bcr-Abl^T315I^.

An important aspect is the conformation adopted by these ligands during their interaction with Bcr-Abl^WT^, as opposed to Bcr-Abl^T315I^. This is because, we hypothesized, that the presence of T315I drastically changes the conformation adopted by the ligands, being a “V” type shape for Bcr-Abl^WT^ and a “T” type shape for Bcr-Abl^T315I^ (Figure 6). In the “T” form, the ligands showed a better fit, aided by the larger size of the hydrophobic region, which easily accommodates the *N*-9 isopentyl group, as well as a better pose for the phenylamino-*N*-hydroxyethyl-piperazine fragment at C-2.

### 3.6. Cell Cycle Cytometry Analysis and Immunodetection

**Figure 7:**
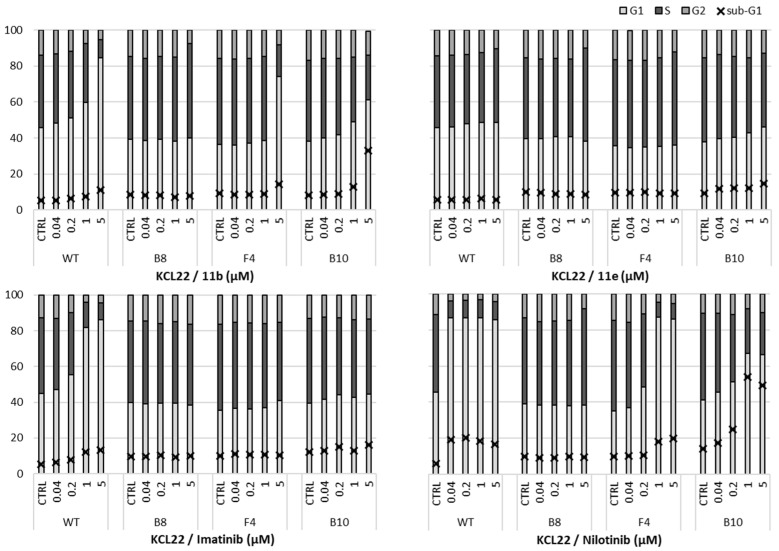
Effects of compounds **11b** and **11e** on the phases of cell cycle (designated as % of G1, S, and G2) and apoptosis (sub-G1) compared to imatinib and nilotinib after 24 h treatment.

To gain insight into the mechanism of action of the selected compounds **11b** and **11e**, we performed cell cycle cytometry analysis of KCL22-derived cells treated for 24 h (Figure 7 and Figure S4). An increasing concentration of **11b** resulted in a significant block of WT cells in G1 phase, with an increase in the percentage of dead cells at 5 µM concentration, which could correspond to Bcr-Abl inhibition, and was comparable to the effect of imatinib. Interestingly, **11b** treatment also arrested F4 and B10 mutant models in G1 phase, in contrast to imatinib treatment, which is consistent with previously shown cytotoxicity assay data. However, the KCL22 B8 cell line containing Bcr-Abl^T315I^ was resistant to treatment with both selected compounds, including **11e**, which had previously shown promising antiproliferative properties in all KCL22 models (GI_50_ = 5.6–6.9 µM). However, this was clearly not reflected in the cell cycle distribution of any of the KCL22-derived models and, as expected, no effect was also observed when treated with imatinib and nilotinib. These results suggest that the compound **11e** probably exerts its antiproliferative effect in KCL22 models through mechanisms other than cell cycle blockade.

**Figure 8:**
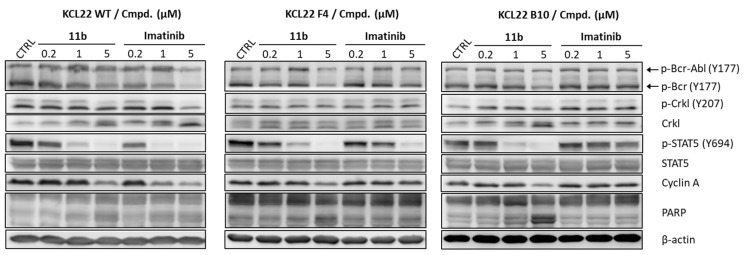
Effects of compounds **11b** and imatinib on the expression or phosphorylation of proteins involved in the proliferation and Bcr-Abl pathways after 24 h treatment.

To determine whether the antiproliferative effect of **11b** was dependent on the inhibition of Bcr-Abl, we immunodetected changes in the expression or phosphorylation of proteins known to be involved in the regulation of proliferation and the Bcr-Abl signaling pathway. The results obtained in **11b**-sensitive WT, F4 and B10 KCL22 cell lines were compared with those obtained after imatinib treatment (Figure 8). Notably, **11b** downregulated the levels of p-Crkl and p-STAT5, downstream proteins of Bcr-Abl, in the WT KCL22 cell line, but to a lesser extent than imatinib. We also observed cleavage of PARP and a decrease of cyclin A levels, both of which are associated with G1 cell cycle arrest and an increase in cell death. These cellular effects of **11b** were significantly pronounced in both mutant cell lines, F4 and B10, especially observed in the decrease in STAT5 phosphorylation and cleavage of PARP. In contrast, imatinib was less effective in the F4 cells and B10 cells were not affected in any of proteins studied.

### 3.7. Calculated Physicochemical Properties and ADME Parameters

**Figure 9:**
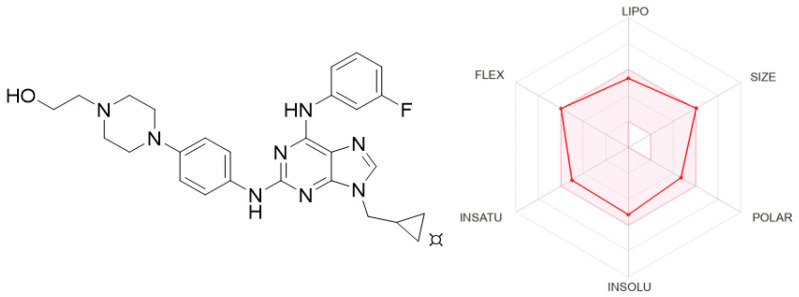
Radar plot for the bioavailability of **11b**. The pink area indicates the range of optimal values for each property related to oral bioavailability. The predicted properties are shown as red lines.

Finally, it should be remembered that both pharmacological properties and pharmacokinetic profiles are important in the drug discovery and development process. It is therefore necessary to be able to predict or determine the latter properties, which relate to administration, distribution, metabolism, and excretion (ADME), and to take them into account during the optimization of a bioactive compound until it becomes a successful candidate for preclinical studies [24]. The free online platform SwissADME (http://www.swissadme.ch/index.php, accessed on 29 January 2024) was used to determine the physicochemical properties of compound **11b** based on Lipinski’s rules. As shown in Table 5, **11b** fulfils the criteria for good permeability and bioavailability based on the values of molecular weight (MW), hydrogen bond donor (HBD), hydrogen bond acceptor (HBA), and cLogP [25]. Furthermore, according to Veber’s rules, **11b** has a topological polar surface area (TPSA) and number of rotatable bonds (NRB) values less than 140 Å^2^ and ≤10 NRB (Table 5) [26]. All these values would indicate that **11b** would have a high ability to penetrate cell membranes and a good oral absorption according to the Lipinski and Veber rules. In addition, the SwissADME platform provides a bioavailability radar plot that considers the following parameters: flexibility (FLEX), lipophilicity (LIPO), solubility (INSOLU), size (SIZE), polarity (POLAR), and saturation (INSATU), and if all parameters are within the desired range (pink region), good oral absorption is expected for this compound. Figure 9 shows that these criteria are met for **11b**.

## 4. Conclusions

Continuing with our search for new Bcr-Abl inhibitors based on purine scaffold, in this work, we report the design and synthesis of 14 new 2,6,9-trisustituted purine derivatives. The biological results showed that the incorporation of *N*-hydroxyethyl-piperazine linked to a phenylamino fragment at C-2 of the purine ring increased the inhibitory activity on Abl as well as the antiproliferative properties on three CML cell lines harboring the Bcr-Abl rearrangement. Notably, **11b** was the most potent inhibitor of Bcr-Abl among all derivatives as well as compared to nilotinib, with an IC_50_ = 0.015 μM. In addition, **11b** showed high cytotoxicity in CML cell lines with a GI_50_ values between 0.7 and 1.3 μM. However, the most interesting results were obtained when these purines were evaluated in a panel of KCL22 subclones expressing different Bcr-Abl point mutants. For B8 (Bcr-Abl^T315I^) and F4 (Bcr-Abl^E255K^) cells, **11b**–**f** were more effective than imatinib and nilotinib in inhibiting the growth of these cells, and more effective than imatinib for B10 cells (Bcr-Abl^Y253H^). Molecular docking studies identified the main interactions of these inhibitors at the binding sites of Bcr-Abl^WT^ and Bcr-Abl^T315I^ and helped to show the difference in potency against both kinases, confirming our hypothesis about the relevance of certain fragments decorating the purine ring. In addition, a possible different binding mode in Bcr-Abl^WT^ and Bcr-Abl^T315I^ was proposed. Furthermore, **11b** was able to inhibit Bcr-Abl in F4 and B10 cells and to arrest the cell cycle in the G1 phase, which correlated with the antiproliferative effects. Finally, **11b** was predicted to have a good pharmacokinetic profile for oral administration. Our results showed that **11b** can be considered as a promising compound for the development of new drugs for CML treatment.

## Figures and Tables

**Table 1:** Inhibition of recombinant Abl1 kinase in vitro by tested compounds.

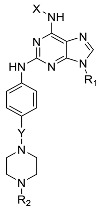					
**Compound**					**IC_50_ (µM) ^a^**
	**X**	**Y**	**R_1_**	**R_2_**	**Abl**
**7a**	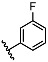	CH_2_	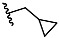	CH_3_	0.180 ± 0.068
**7b**	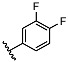	CH_2_	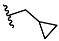	CH_3_	0.225 ± 0.042
**11a**	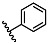	--	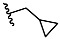	CH_2_CH_2_OH	0.037 ± 0.012
**11b**	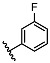	--	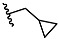	CH_2_CH_2_OH	0.015 ± 0.010
**11c**	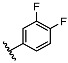	--	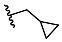	CH_2_CH_2_OH	0.020 ± 0.001
**11d**	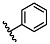	--	isopentyl	CH_2_CH_2_OH	1.24 ± 0.22
**11e**	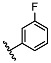	--	isopentyl	CH_2_CH_2_OH	0.131 ± 0.071
**11f**	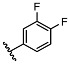	--	isopentyl	CH_2_CH_2_OH	1.76 ± 0.74
**11g**	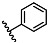	--	*n*-hexyl	CH_2_CH_2_OH	0.834 ± 0.447
**11h**	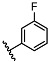	--	*n*-hexyl	CH_2_CH_2_OH	0.674 ± 0.069
**11i**	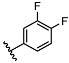	--	*n*-hexyl	CH_2_CH_2_OH	1.68 ± 0.01
**13a**	H	--	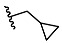	CH_3_	3.96 ± 0.51
**13b**	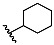	--	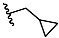	CH_3_	11.06 ± 3.31
**13c**	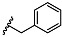	--	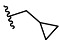	CH_3_	0.845 ± 0.066
**I**	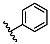	--	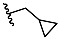	CH_3_	0.090 ± 0.007 ^b^
**II**	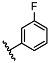	--	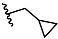	CH_3_	0.040 ± 0.013 ^b^
**III**	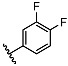	--	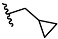	CH_3_	0.040 ± 0.013 ^b^
Imatinib					0.327 ± 0.121
Nilotinib					0.047 ± 0.039

^a^ IC_50_ values were determined in at least three independent experiments. ^b^ Values reported in [12].

**Table 2:** In vitro cytotoxicity of tested compounds on cancer cell lines and non-neoplastic cells HEK-293T.

	GI_50_ (μM) ^a^			
Compound	KBM5	BV173	K562	HEK-293T
**7a**	4.4 ± 0.8	3.3 ± 1.6	1.5 ± 0.1	6.3 ± 0.7
**7b**	4.2 ± 0.2	1.9 ± 0.1	1.4 ± 0.2	6.3 ± 1.2
**11a**	3.0 ± 0.1	3.0 ± 0.6	1.2 ± 0.3	9.5 ± 0.7
**11b**	1.3 ± 0.1	1.5 ± 0.2	0.7 ± 0.2	5.5 ± 0.6
**11c**	2.1 ± 0.3	1.7 ± 0.1	0.9 ± 0.2	5.0 ± 0.9
**11d**	5.6 ± 0.4	3.6 ± 0.7	6.3 ± 0.7	5.7 ± 1.2
**11e**	5.0 ± 0.1	3.8 ± 0.1	4.3 ± 0.2	4.7 ± 0.7
**11f**	5.2 ± 0.1	4.5 ± 0.1	5.9 ± 0.6	5.2 ± 0.7
**11g**	5.4 ± 0.2	5.2 ± 0.2	4.5 ± 1.4	8.6 ± 3.2
**11h**	5.8 ± 0.2	5.2 ± 0.5	4.8 ± 0.4	6.0 ± 1.0
**11i**	5.6 ± 0.2	4.8 ± 0.3	3.1 ± 0.6	>20
**13a**	>100	>20	>100	>100
**13b**	>20	>20	4.8 ± 0.3	>20
**13c**	>20	11.9 ± 1.4	5.7 ± 0.6	16.7 ± 1.1
**I**	2.7 ± 0.1	2.2 ± 0.2	1.5 ± 0.1	7.5 ± 1.4
**II**	5.1 ± 0.5	5.3 ± 0.2	0.8 ± 0.1	5.6 ± 0.6
**III**	1.1 ± 0.8	1.4 ± 0.1	1.2 ± 0.4	5.3 ± 0.3
Imatinib	0.482 ± 0.311	0.199 ± 0.061	0.231 ± 0.001	>10
Nilotinib	0.019 ± 0.009	0.003 ± 0.000	0.025 ± 0.010	>10

^a^ GI_50_ values were determined in at least three independent experiments using the resazurin assay.

**Table 3:** GI_50_ values of tested compounds for KCL22 cells containing Bcr-Abl^WT^ or mutated kinase domain in T315I (B8), E255K (F4), or Y253H (B10).

	GI_50_ KCL22 (μM) ^a^			
Compound	WT -	B8 T315I (100%)	F4 E255K (100%)	B10 Y253H (50%)
**7a**	8.1 ± 0.5	13.8 ± 2.0	11.1 ± 0.5	7.4 ± 0.6
**7b**	8.5 ± 1.4	11.4 ± 3.2	10.1 ± 1.2	6.6 ± 0.2
**11a**	5.7 ± 0.8	>20	12.7 ± 0.1	6.3 ± 0.4
**11b**	2.0 ± 0.4	11.5 ± 1.1	4.1 ± 0.1	2.6 ± 0.2
**11c**	2.9 ± 0.0	6.4 ± 0.4	4.1 ± 0.2	4.1 ± 0.2
**11d**	8.4 ± 0.3	7.8 ± 1.3	6.6 ± 0.6	7.8 ± 0.5
**11e**	6.1 ± 0.1	6.9 ± 0.8	5.6 ± 0.5	6.9 ± 0.1
**11f**	6.5 ± 0.2	7.2 ± 1.0	5.7 ± 0.1	7.0 ± 0.5
**11g**	15.1 ± 2.2	10.4 ± 1.8	8.5 ± 0.9	7.7 ± 0.2
**11h**	9.6 ± 1.0	9.5 ± 1.5	8.1 ± 1.2	5.9 ± 0.7
**11i**	>20	>20	>20	>20
**13a**	>100	>100	>100	>100
**13b**	>20	>20	>20	>20
**13c**	>20	>20	>20	>20
**I**	3.4 ± 0.1	17.3 ± 2.0	5.7 ± 0.3	5.2 ± 0.2
**II**	10.6 ± 0.9	>20	>20	8.8 ± 0.4
**III**	18.3 ± 1.7	>20	>20	3.4 ± 0.4
Imatinib	0.582 ± 0.027	>20	9.6 ± 0.5	>20
Nilotinib	0.047 ± 0.008	>20	0.789 ± 0.081	1.4 ± 0.3

^a^ GI_50_ values were determined in at least three independent experiments using the resazurin assay.

**Table 4:** MM-GBSA and energy contribution calculations (kcal/mol) for ligand–Bcr-Abl^T315I^ complex.

Compound	∆G Bind	∆G Coul.	∆G H Bond	∆G Lipo	∆G Packing	∆G SelfCont	∆G Solv_GB	∆G vdW
**11b**	−64.41	−50.09	−1.52	−29.10	−4.24	0.42	61.64	−50.55
**11c**	−65.17	−55.93	−2.31	−28.50	−3.71	0.37	65.65	−48.54
**11d**	−69.82	−50.24	−1.90	−30.20	−3.69	0.67	64.65	−57.76
**11e**	−71.67	−54.62	−3.05	−30.02	−3.11	0.15	65.42	−56.74
**11f**	−70.14	−54.22	−2.69	−29.63	−4.01	0.36	62.97	−52.97

**Table 5:** Calculated physicochemical properties and ADME parameters for **11b**.

Compound	MW (Da)	HBA	HBD	cLogP	TPSA (Å^2^)	NRB
Desirable value	≤500	≤10	≤5	≤5	≤140	≤10
**11b**	502.59	6	3	3.30	94.37	9

All values predicted by SwissADME (http://www.swissadme.ch/index.php, accessed on 29 January 2024).

## Data Availability

The data presented in this study are available on request from the corresponding author.

## Supplementary Materials

The following supporting information can be downloaded at: https://www.mdpi.com/article/10.3390/pharmaceutics16050649/s1, ^1^H-, ^13^C-, and ^19^F-NMR and HRMS of selected compounds. Table S1. Binding affinity scores (kcal/mol) of the purine derivatives with RDOCK, XP Score and MM-GBSA (∆G_bind_ and their respective energy contributions) in Bcr-Abl^WT^. Figure S1. Co-crystallized ligand, purvalanol, in the Bcr-Abl binding site. Its experimentally determined binding mode is shown in blue, while the docking pose of purvalanol from our self-docking protocol is shown in green. Figure S2. Correlation plot between biological activity in pIC_50_ and affinity energies achieved with RDOCK, Glide (XP Score), and MM-GBSA of the synthesized compounds. Figure S3. Correlation plot between biological activity in pGI_50_ values on B8 cells and affinity energies achieved with MM-GBSA of the selected compounds. Figure S4. Histograms of flow cytometry of selected compounds on KCL cells and their subclones.
