# Supplementary material for: New Inhibitors of Bcr-Abl Based on 2,6,9-Trisubstituted Purine Scaffold Elicit Cytotoxicity in Chronic Myeloid Leukemia-Derived Cell Lines Sensitive and Resistant to TKIs
Source: Pharmaceutics. 2024 May 11;16(5):649. doi: 10.3390/pharmaceutics16050649 (PMC11125181; doi:10.3390/pharmaceutics16050649)

Supporting Information

New inhibitors of Bcr-Abl based on 2,6,9-trisubstituted purine scaffold elicited cytotoxicity in chronic myeloid leukaemia-derived cell lines sensitive and resistant to TKIs

Thalia Delgado,[1] Denisa Veselá,[2] Hana Dostálová,[2] Vladimír Kryštof,[2,3*] Veronika Vojáčková,[2] Radek Jorda,[2] Alejandro Castro,[4] Jeanluc Bertrand,[1] Gildardo Ribera,[5] Mario Faúndez,[6] Miroslav Strnad,[7] Christian Espinosa-Bustos[6] and Cristian O. Salas[1,*]

1 Departamento de Química Orgánica, Facultad de Química y de Farmacia, Pontificia Universidad Católica de Chile, Santiago de Chile 702843, Chile

2 Department of Experimental Biology, Palacký University Olomouc, Šlechtitelů 27, 783 71 Olomouc, Czech Republic

3 Institute of Molecular and Translational Medicine, Faculty of Medicine and Dentistry, Palacký University Olomouc, Hněvotínská 5, 779 00 Olomouc, Czech Republic

4 Laboratorio de Bioproductos Farmacéuticos y Cosméticos, Centro de Excelencia en Medicina Traslacional, Facultad de Medicina, Universidad de La Frontera, Av. Francisco Salazar 01145, Temuco 4780000, Chile

5 Laboratorio de Biotecnología Farmacéutica, Centro de Biotecnología Genómica, Instituto Politécnico Nacional, Boulevard del Maestro s/n, Reynosa 88710, Mexico

6 Departamento de Farmacia, Facultad de Química y de Farmacia, Pontificia Universidad Católica de Chile, Santiago de Chile 702843, Chile

7 Laboratory of Growth Regulators, Institute of Experimental Botany of the Czech Academy of Sciences & Palacký University, Šlechtitelů 27, 783 71 Olomouc, Czech Republic

Index

^{1}H,^{13}C NMR and ^{19}F spectra of final compounds..pags 2-19

HRMS of final compounds..pags 20-26

Table S1. Binding affinity scores of the purine derivatives in the Bcr-Abl^{WT}...............pag 27

Figure S1. Co-crystallized ligand, purvalanol, in the Bcr-Abl binding site...............pag 28

Figure S2. Correlation plot between biological activity in pIC_{50} and affinity energies of the synthesised compounds...pag 29

Figure S3. Correlation plot between biological activity in pGI_{50} values on B8 cells and affinity energies of the selected compounds...pag30

Figure S4. Histograms of flow cytometry of selected compounds on KCL cells and their subclones..pags31-33

^{1}H NMR spectra of compound **7a**

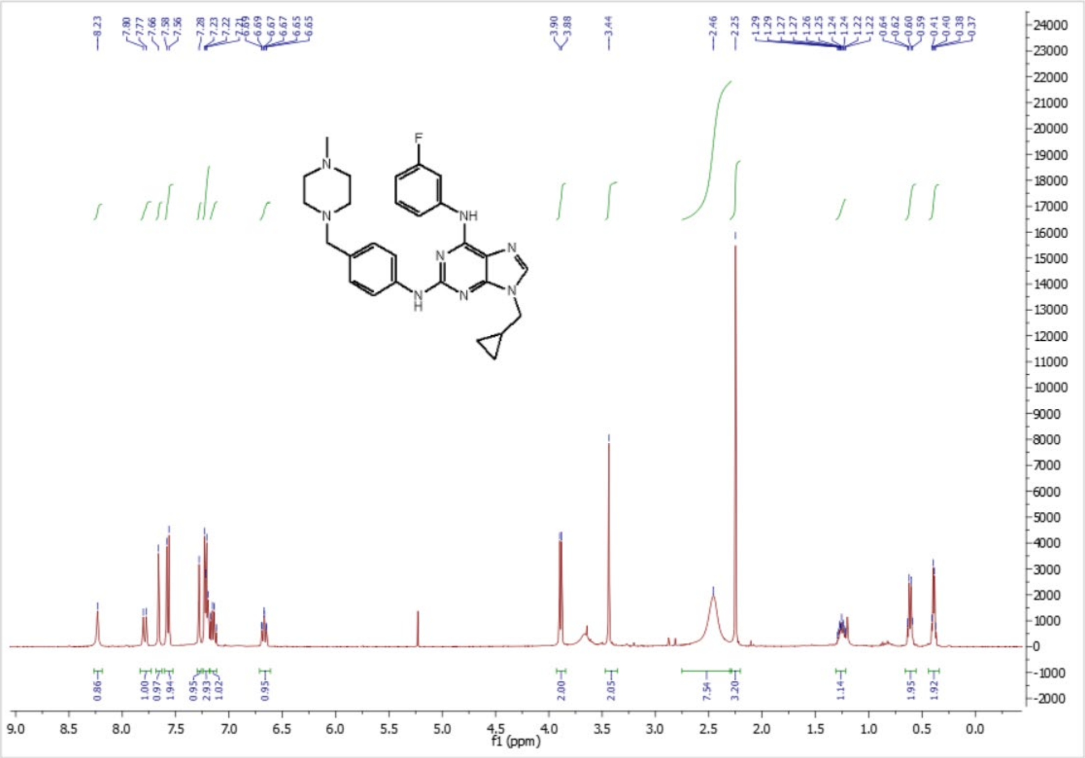

^{13}C NMR spectra of compound **7a**

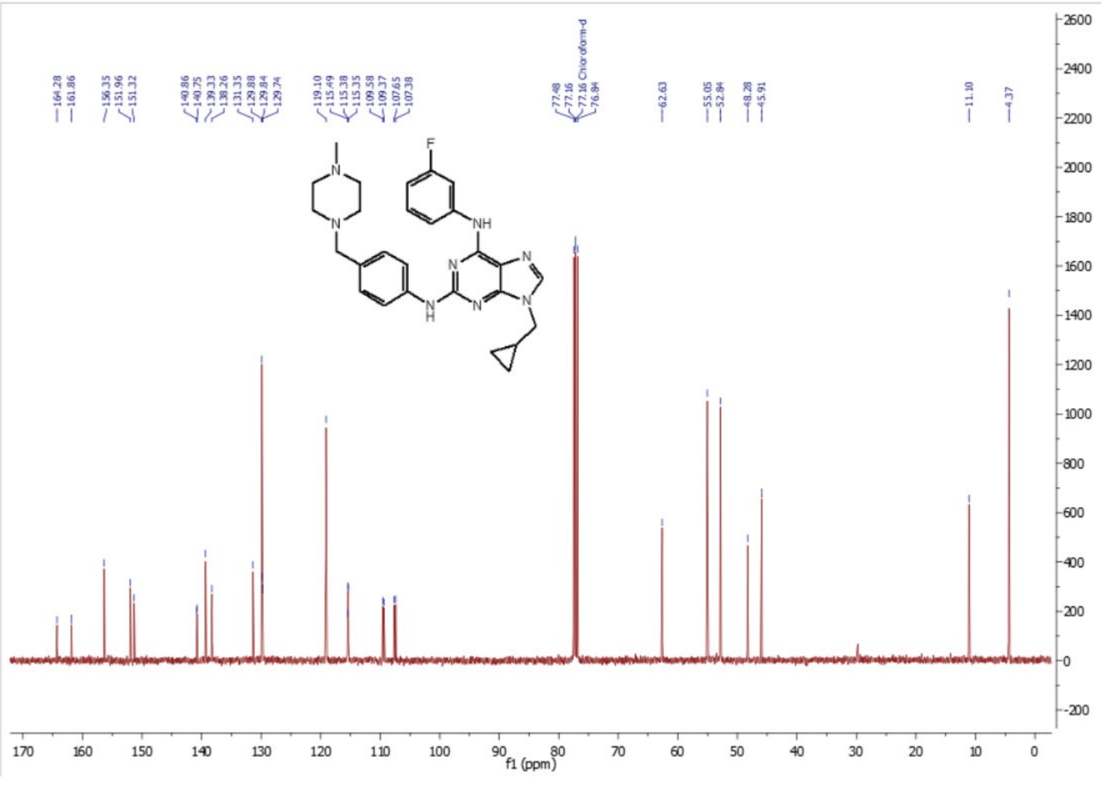

^{19}F NMR spectra of compound **7a**

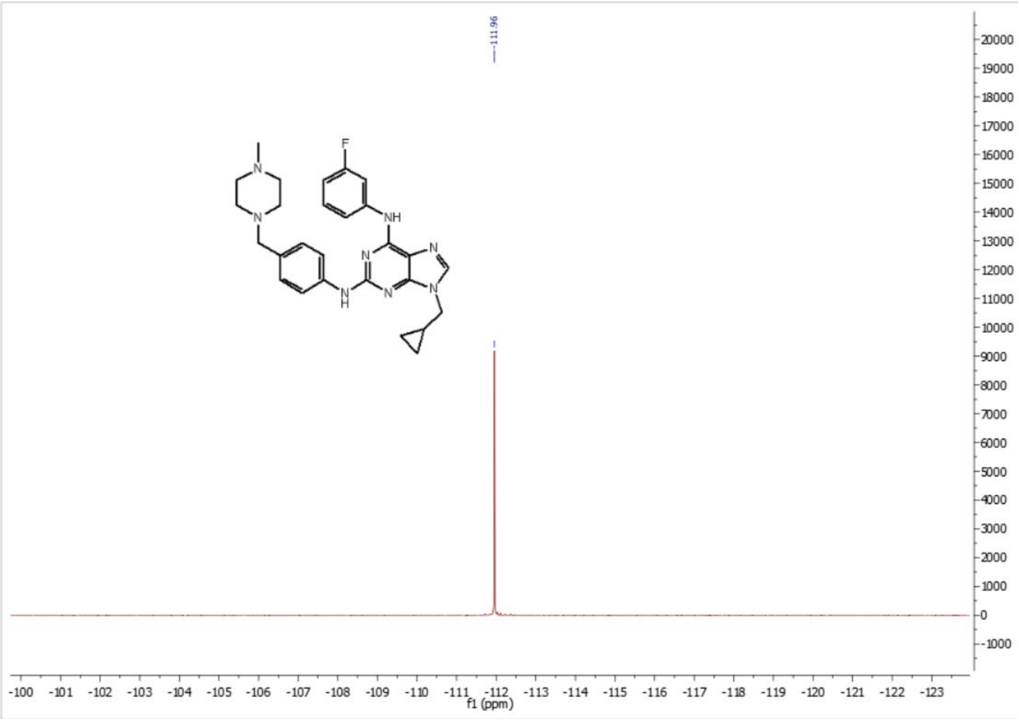

^{1}H NMR spectra of compound **7b**

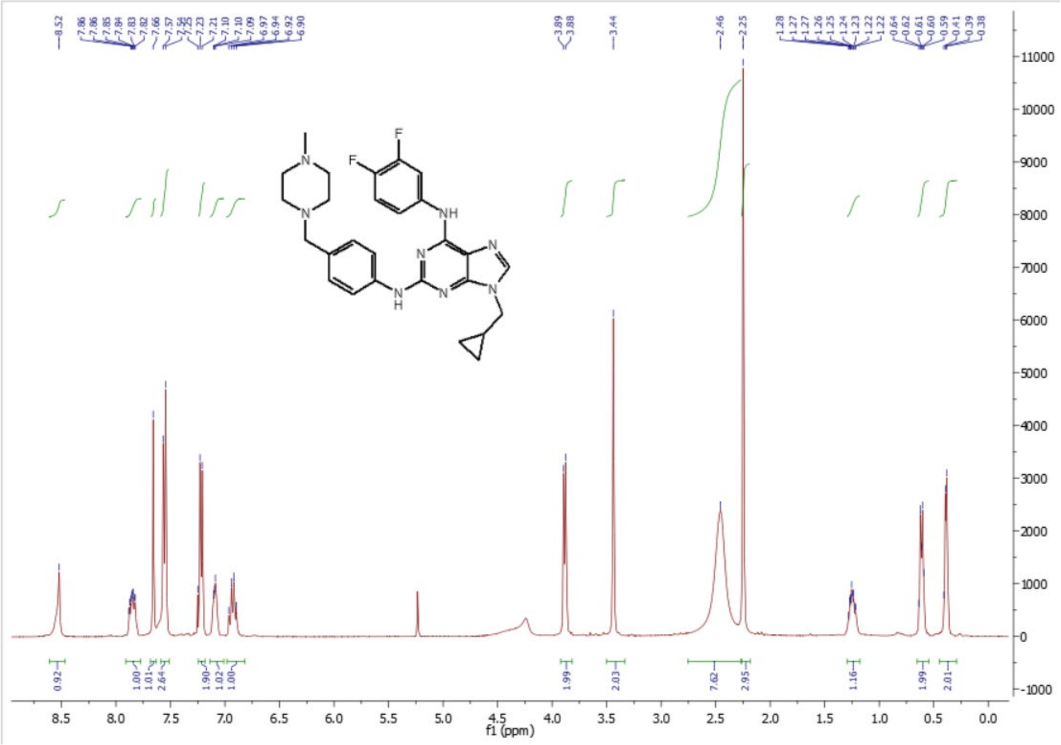

^{13}C NMR spectra of compound **7b**

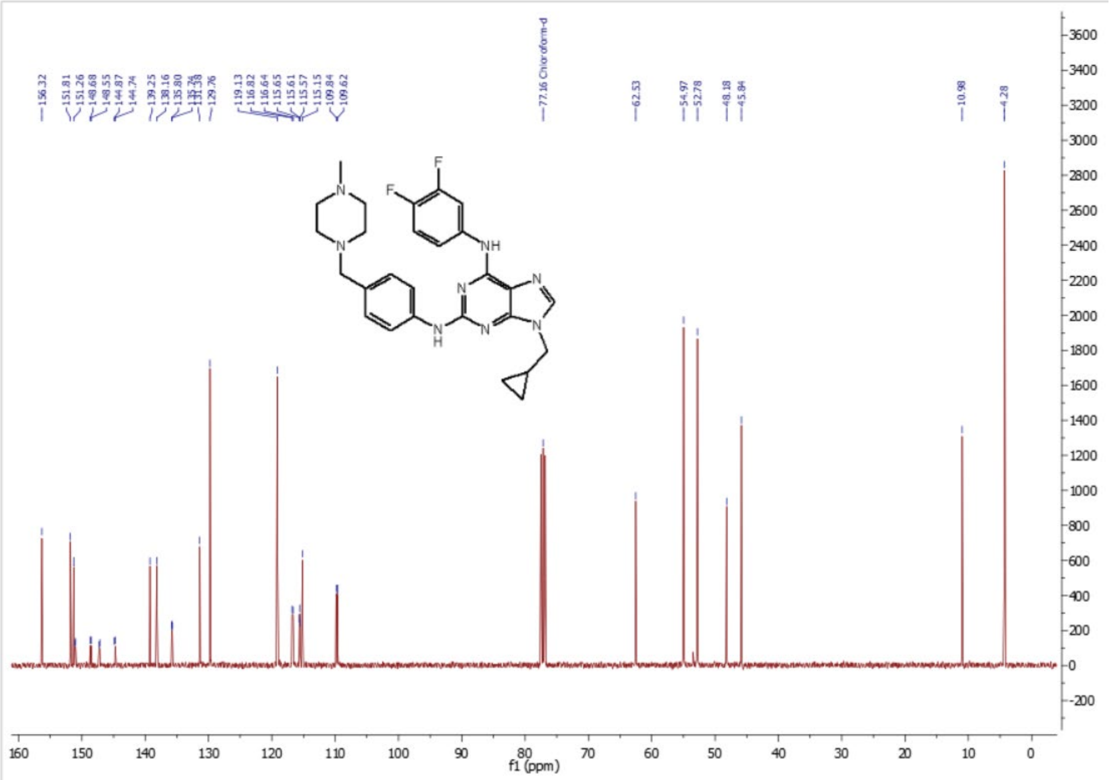

^{19}F NMR spectra of compound **7b**

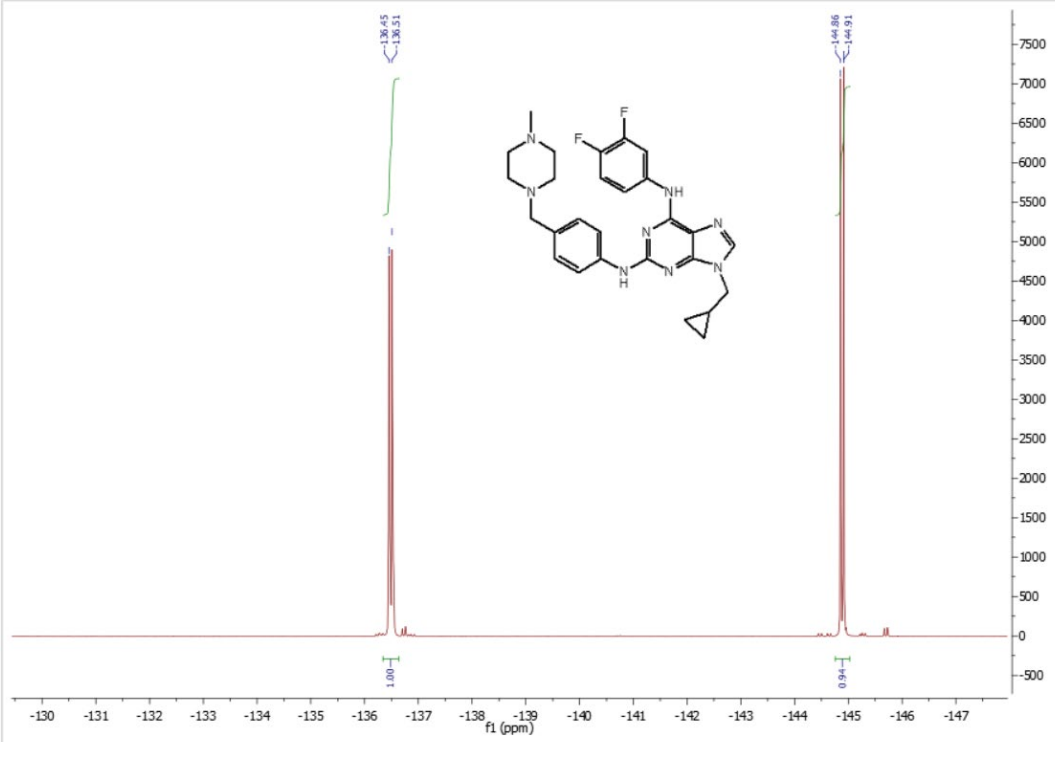

^{1}H NMR spectra of compound **11a**

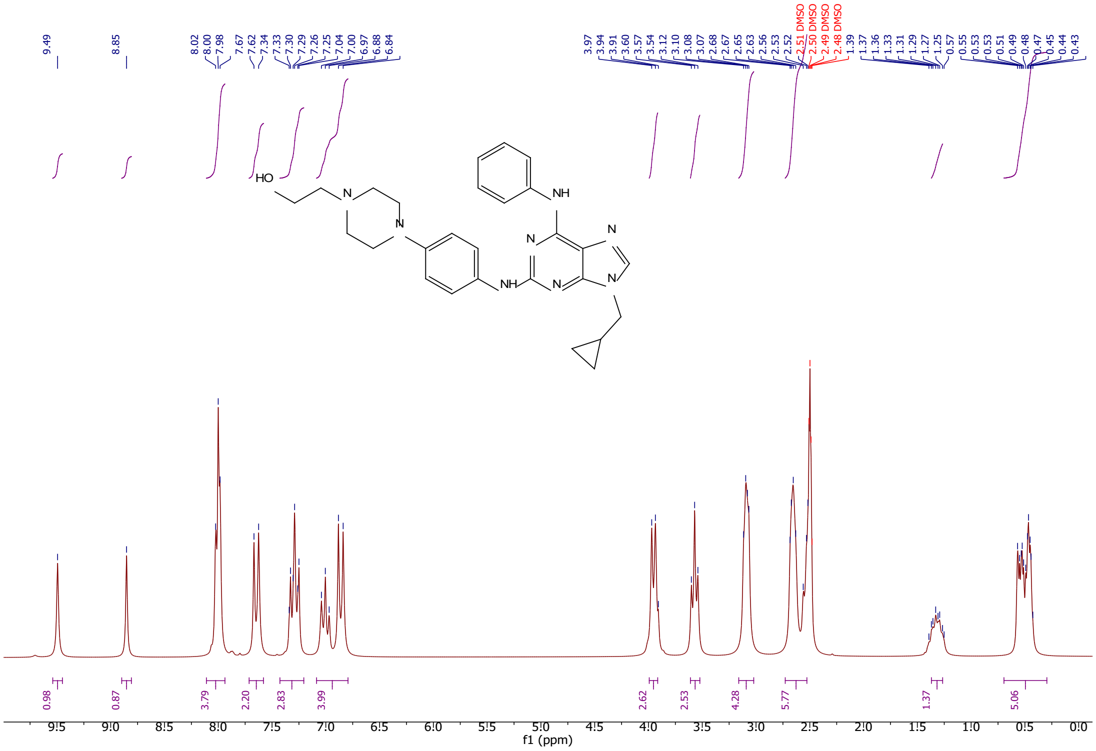

^{13}C NMR spectra of compound **11a**

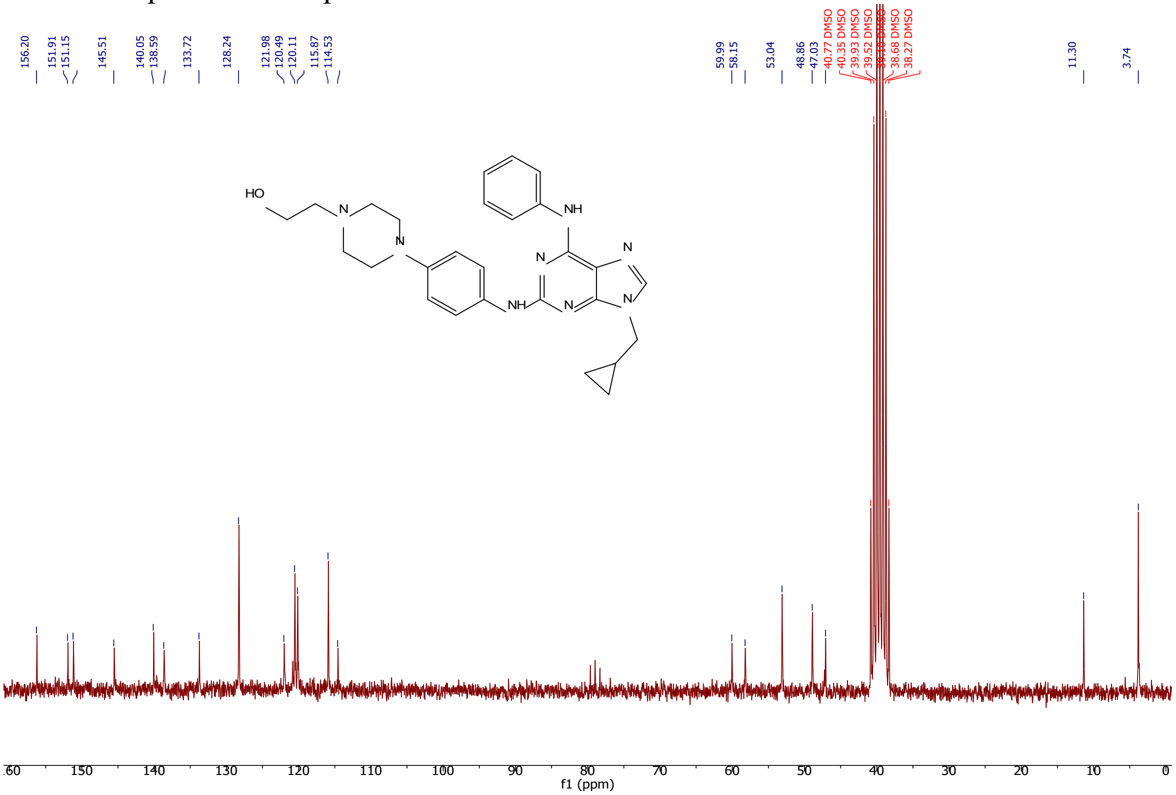

^{1}H NMR spectra of compound **11b**

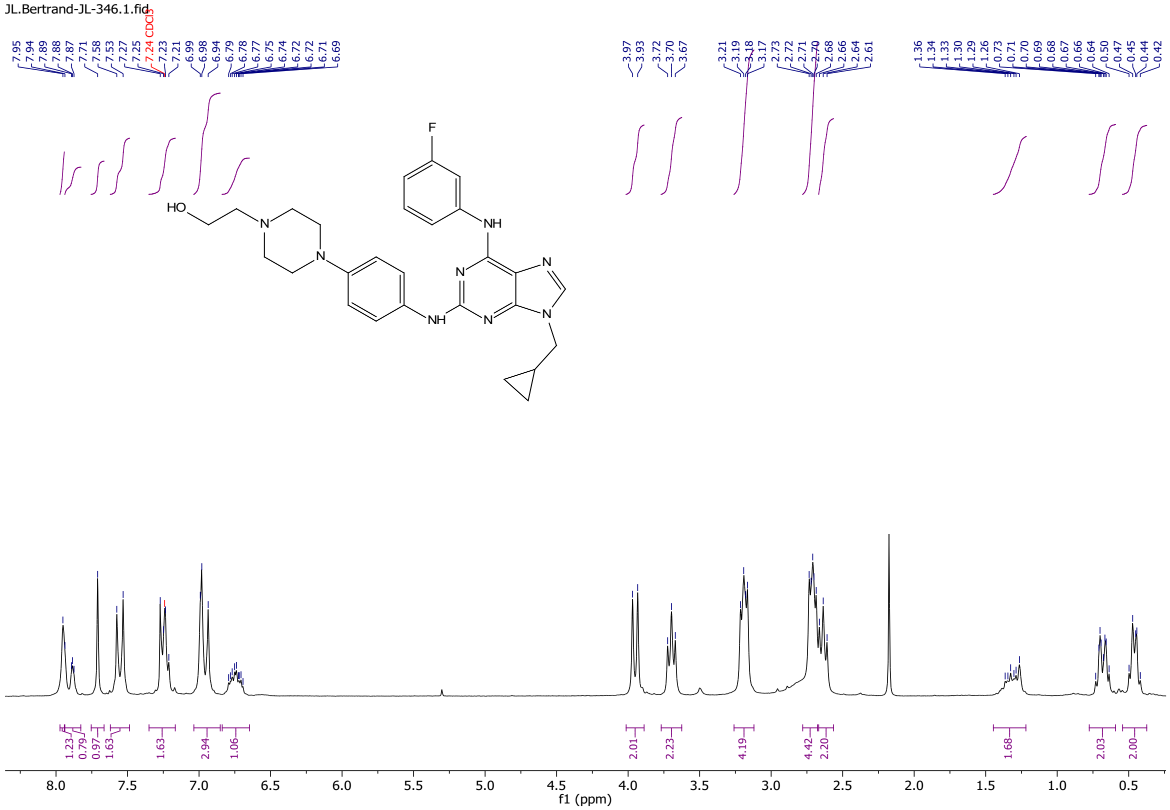

^{13}C NMR spectra of compound **11b**

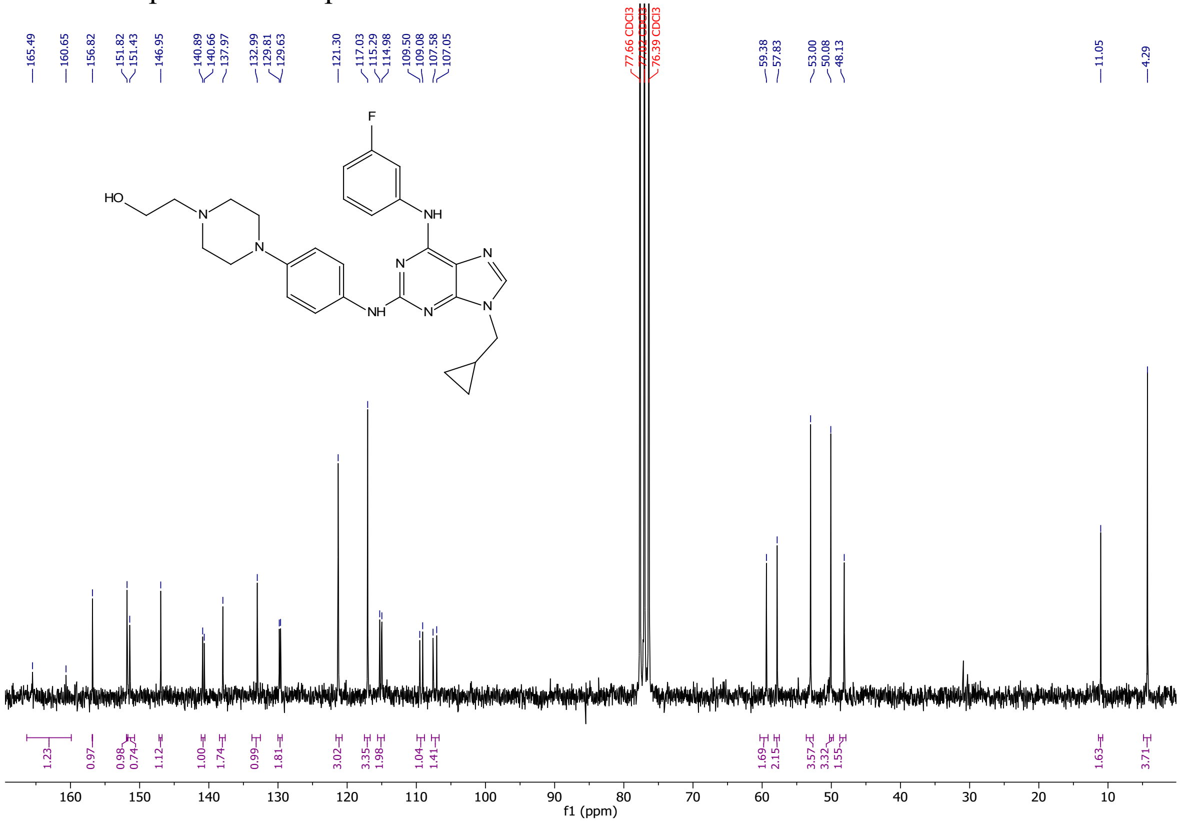

^{19}F NMR spectra of compound **11b**

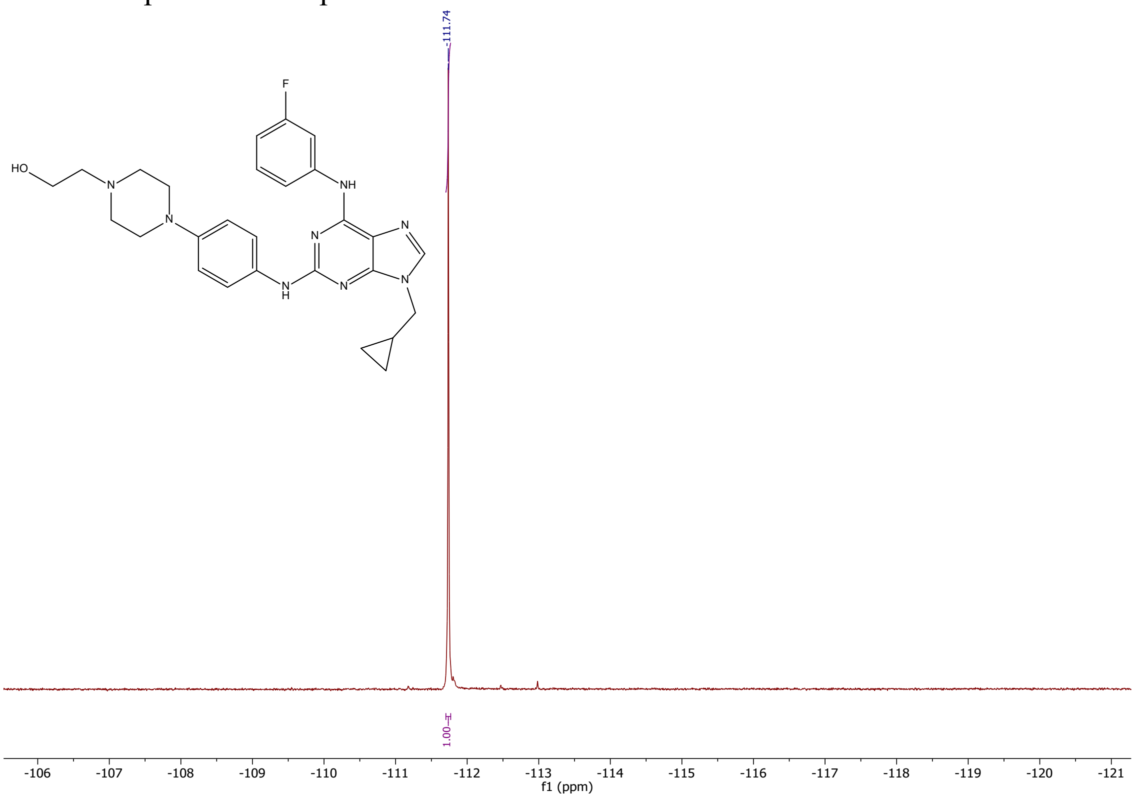

^{1}H NMR spectra of compound **11c**

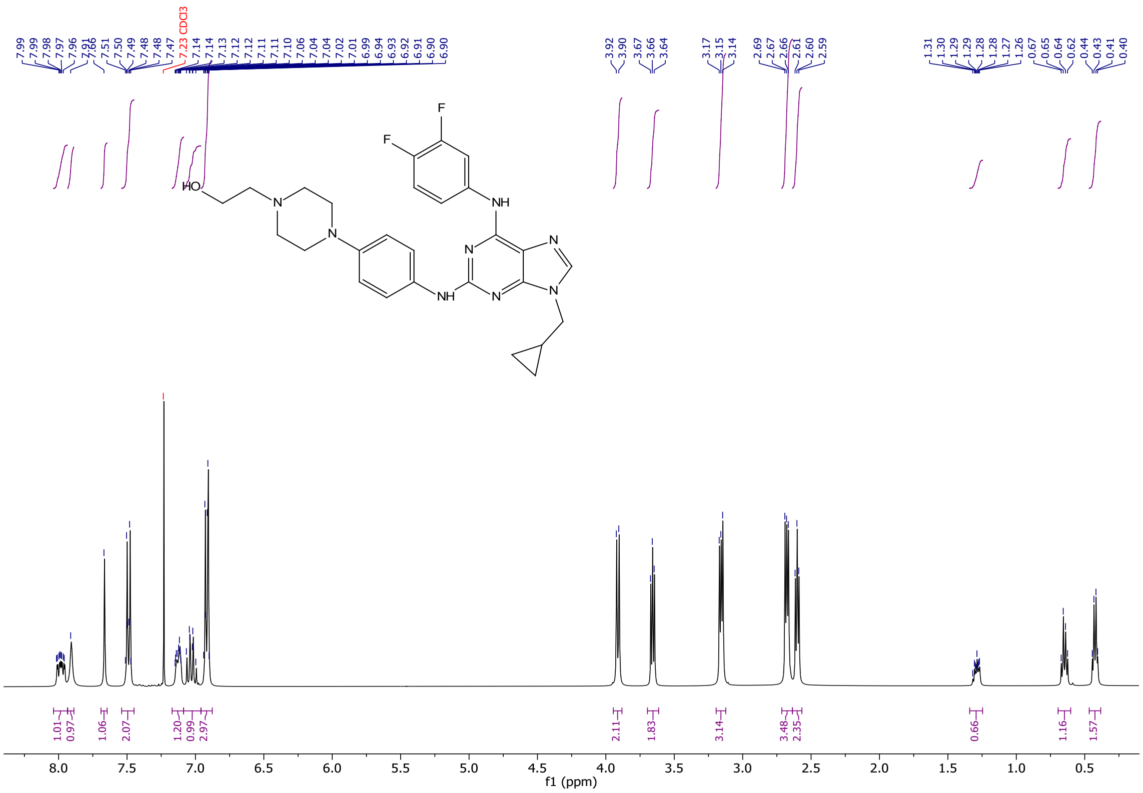

^{13}C NMR spectra of compound **11c**

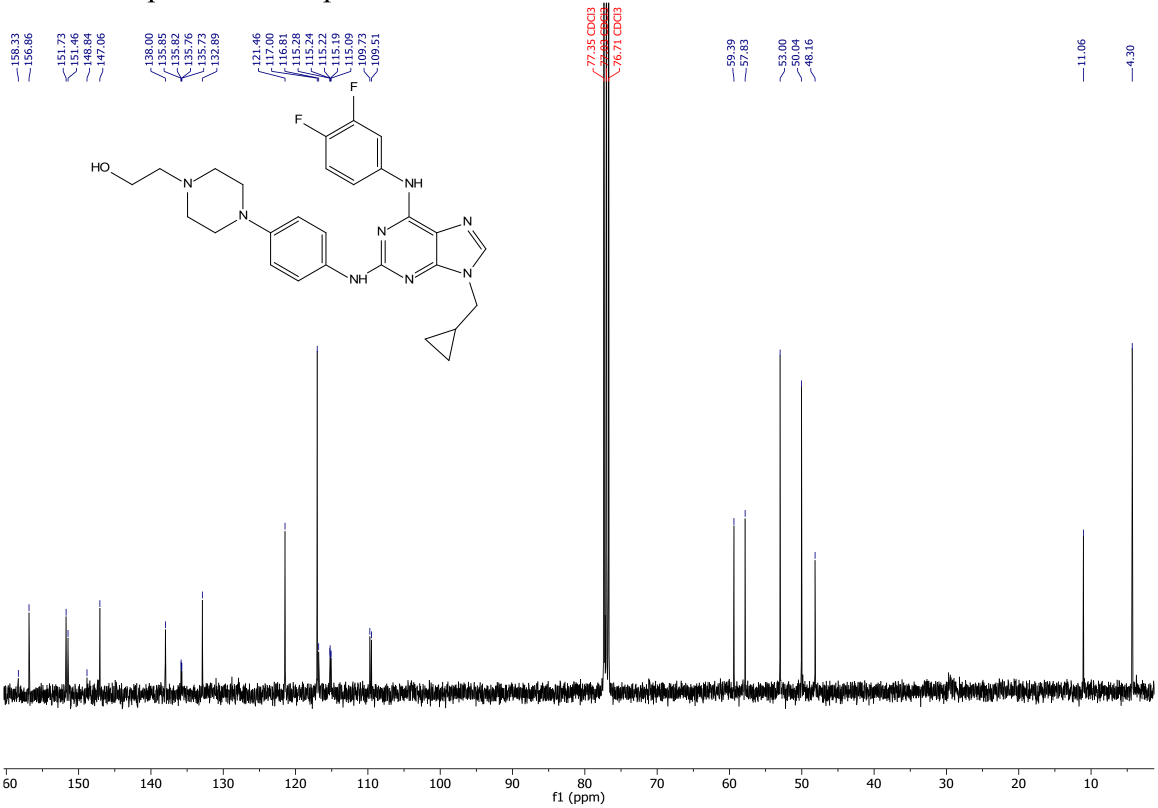

^{19}F NMR spectra of compound **11c**

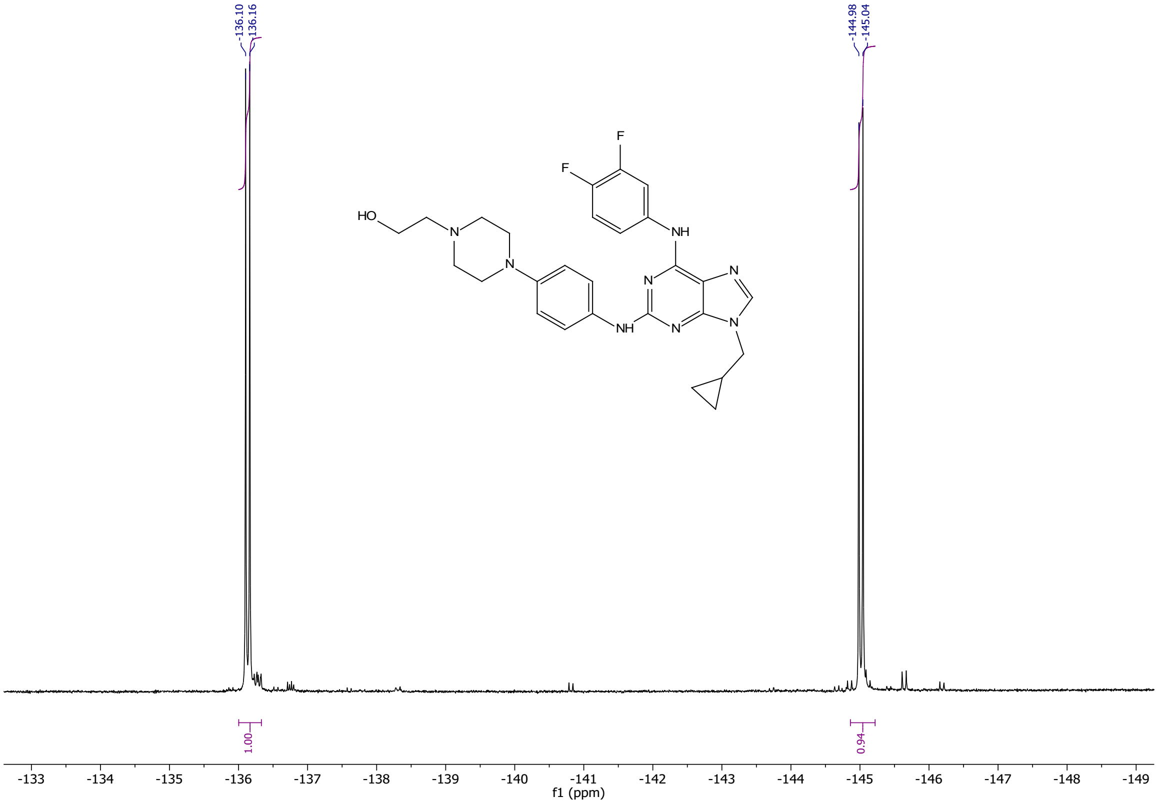

^{1}H NMR spectra of compound **11d**

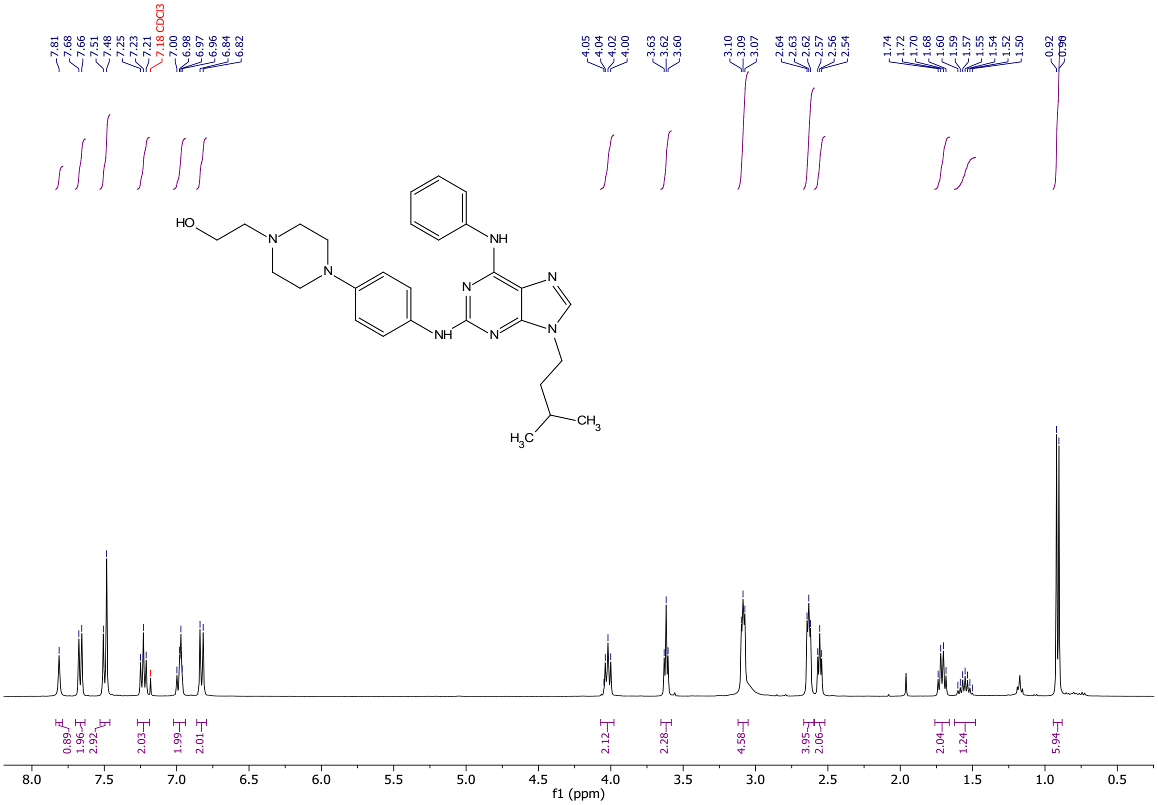

^{13}C NMR spectra of compound **11d**

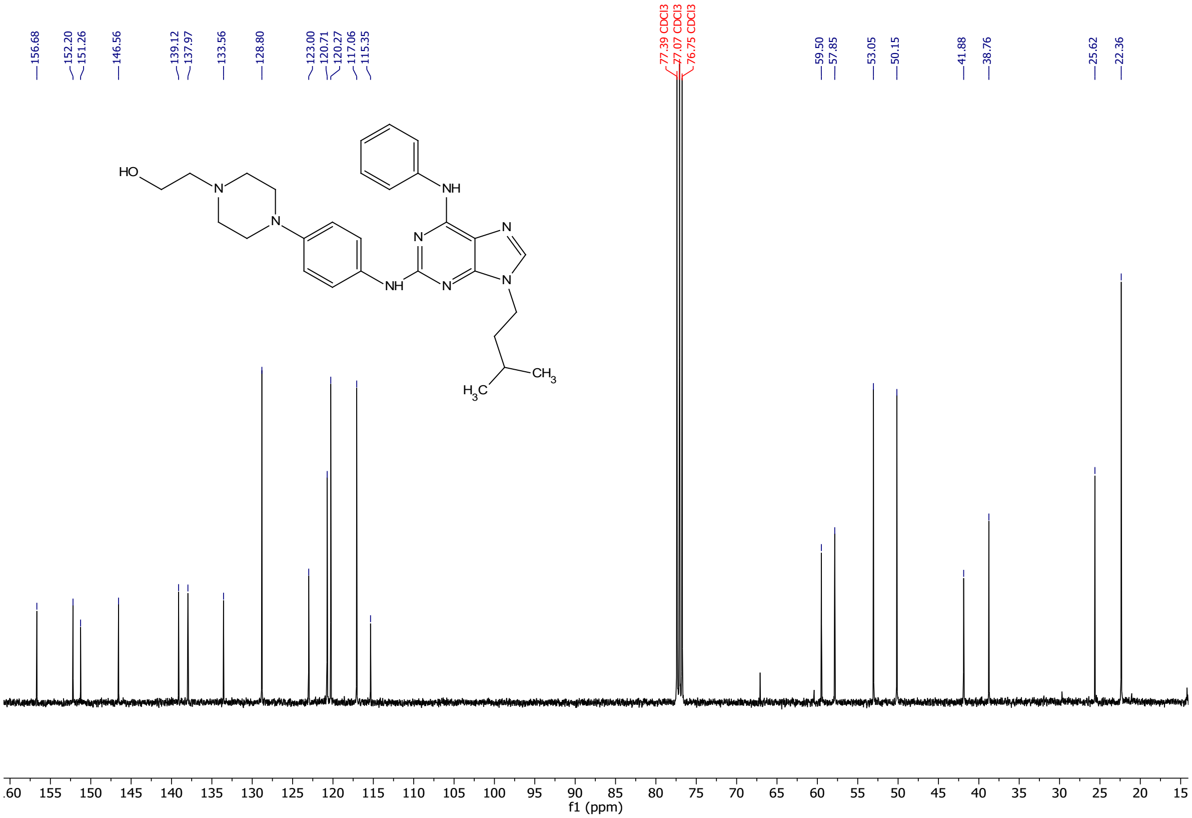

^{1}H NMR spectra of compound **11e**

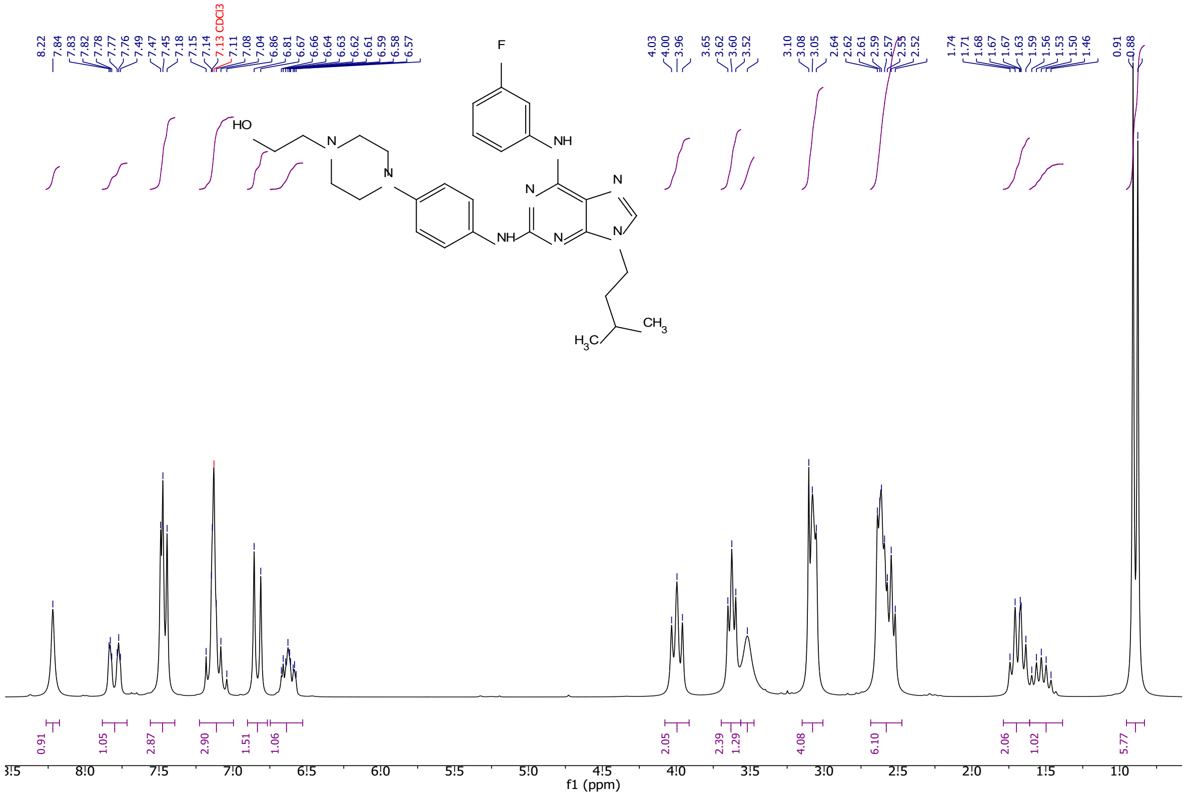

^{13}C NMR spectra of compound **11e**

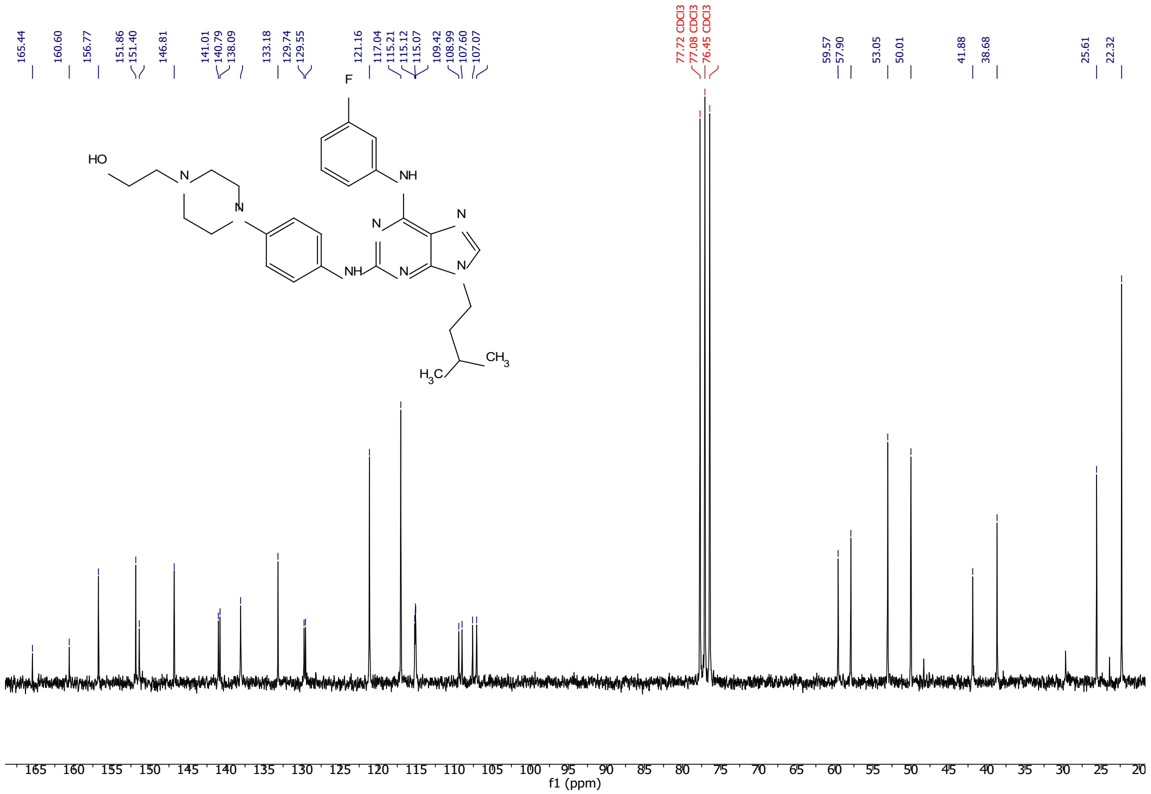

^{19}F NMR spectra of compound **11e**

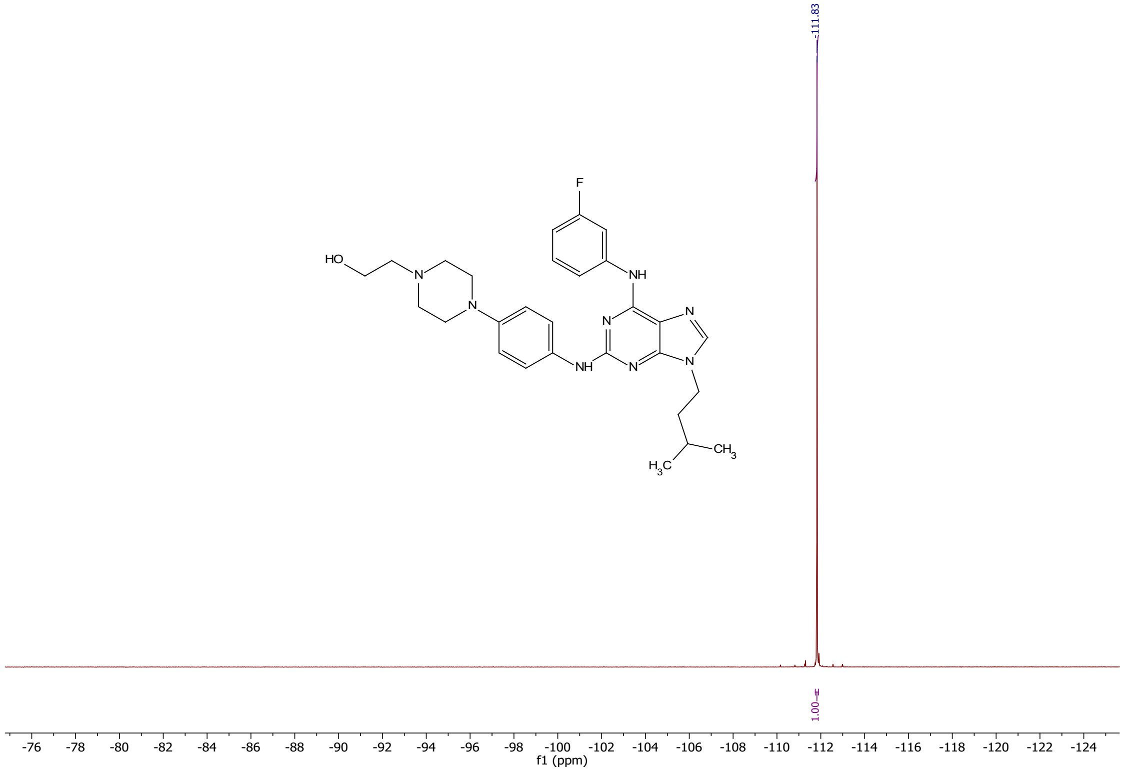

^{1}H NMR spectra of compound **11f**

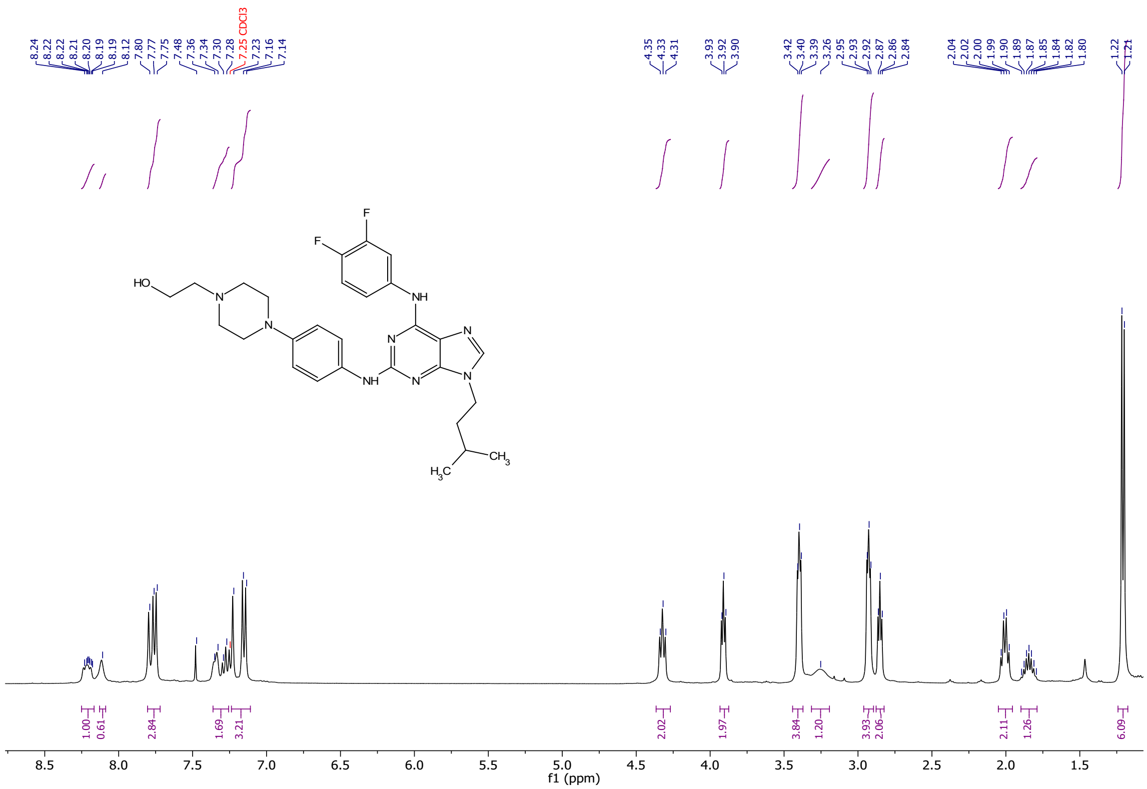

^{13}C NMR spectra of compound **11f**

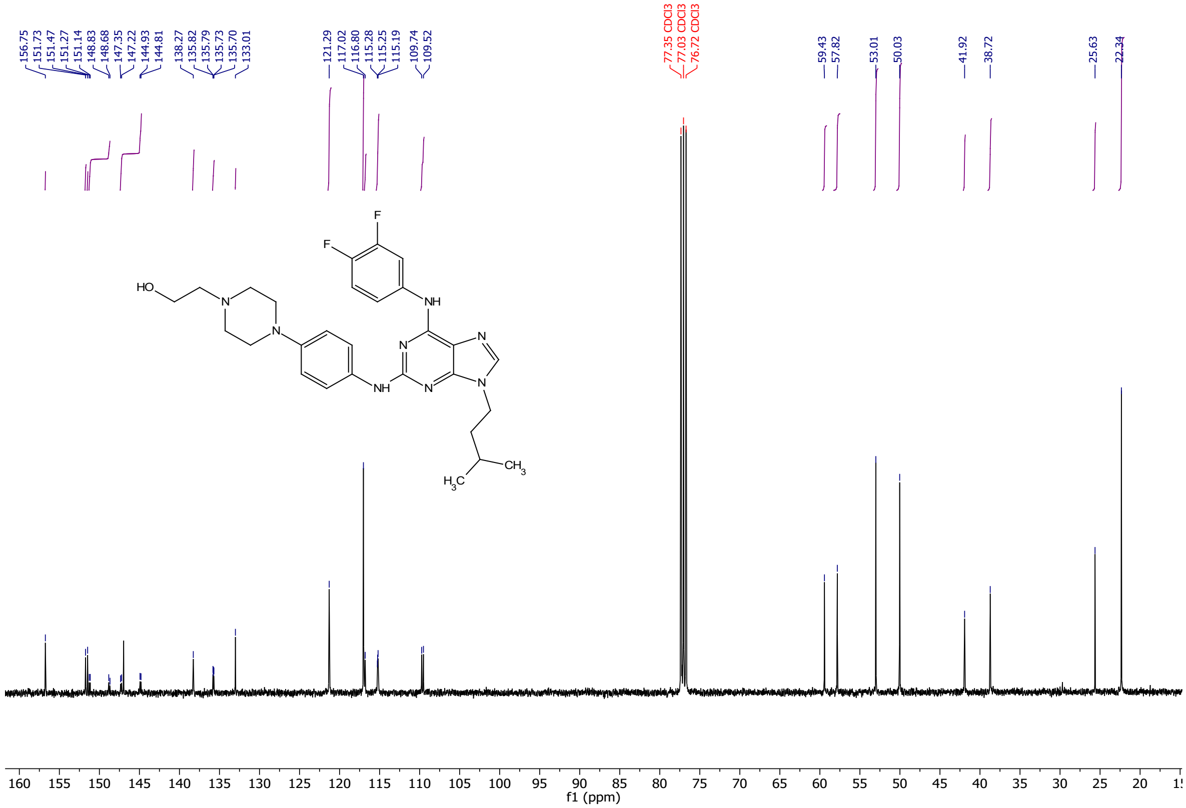

^{19}F NMR spectra of compound **11f**

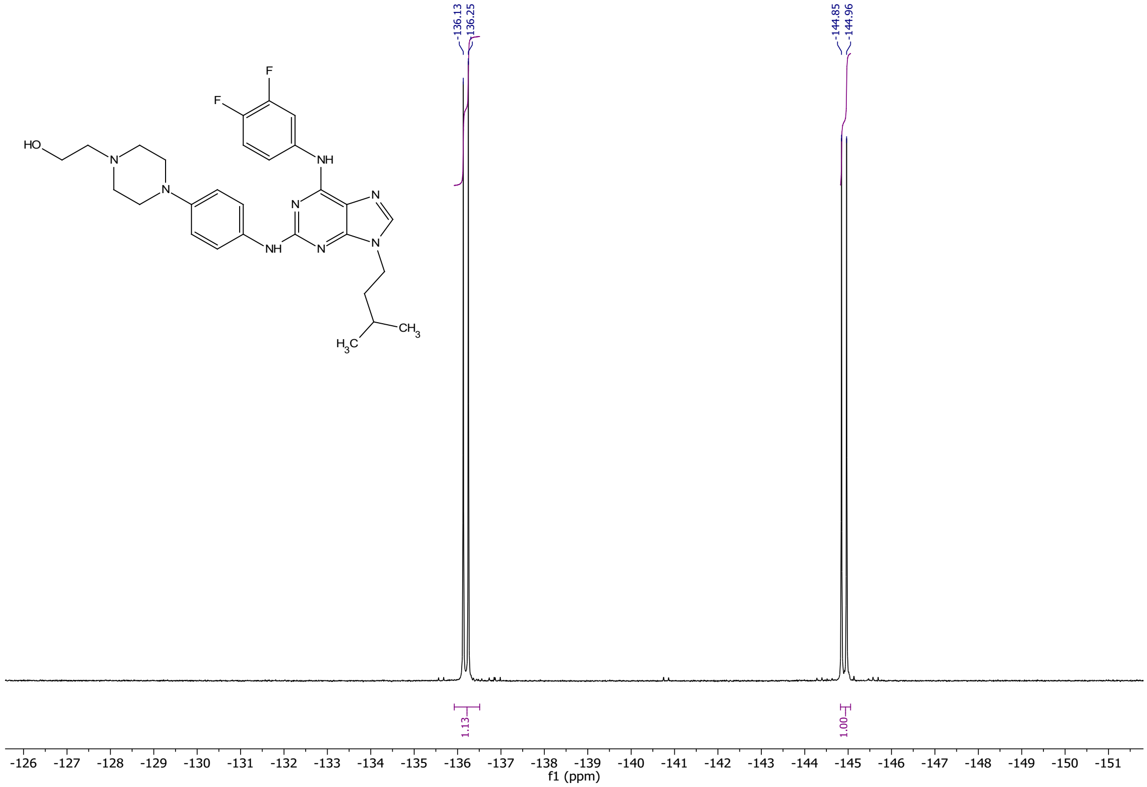

^{1}H NMR spectra of compound **11g**

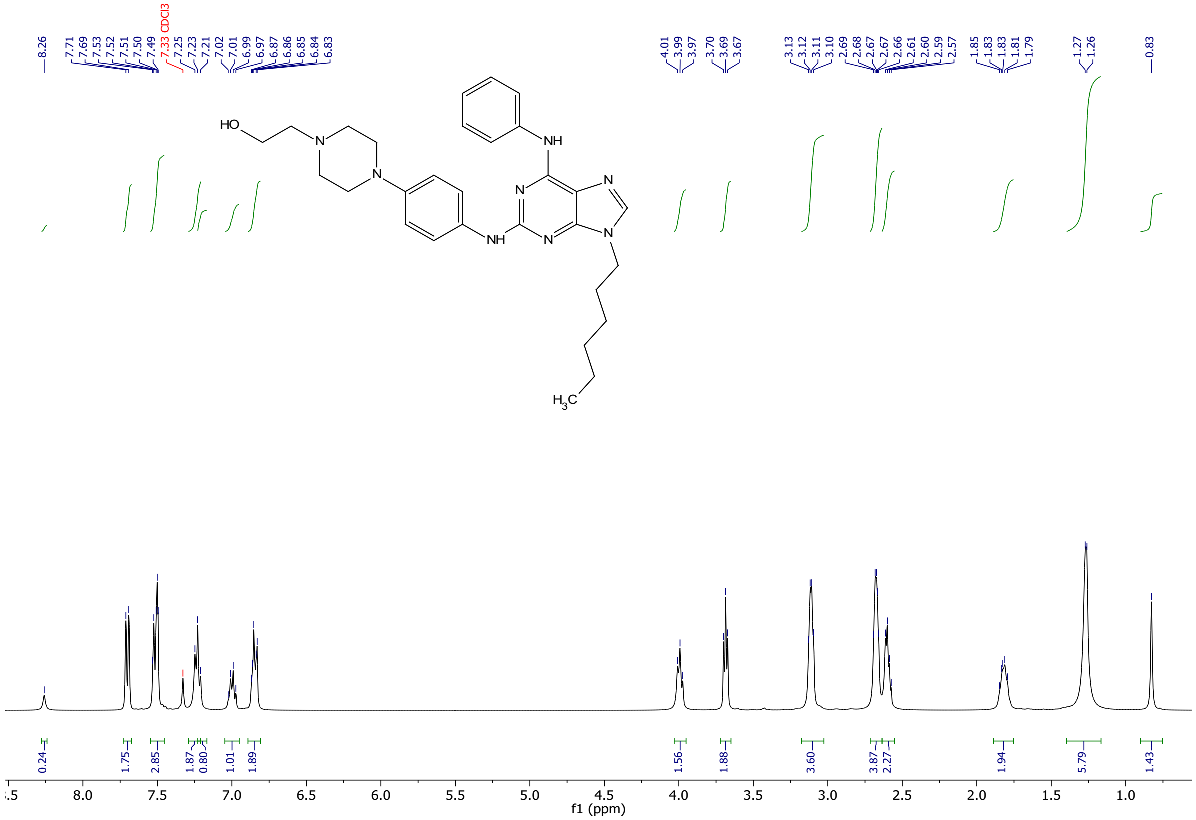

^{13}C NMR spectra of compound **11g**

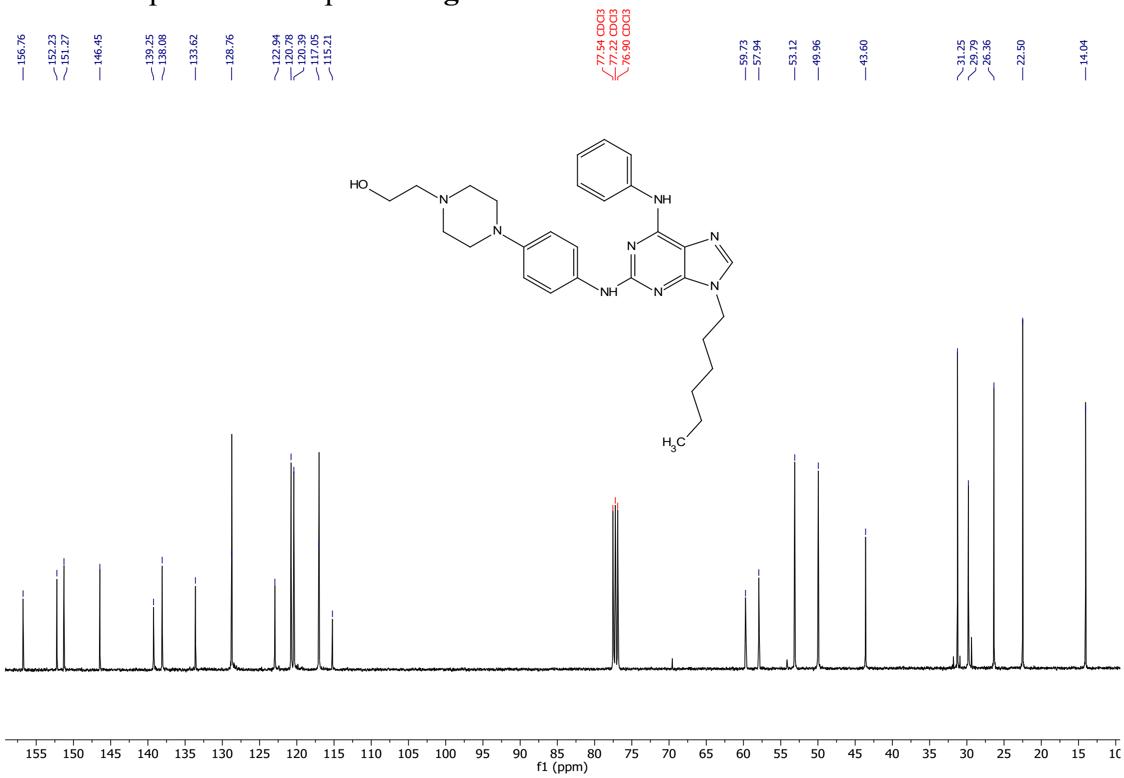

^{1}H NMR spectra of compound **11h**

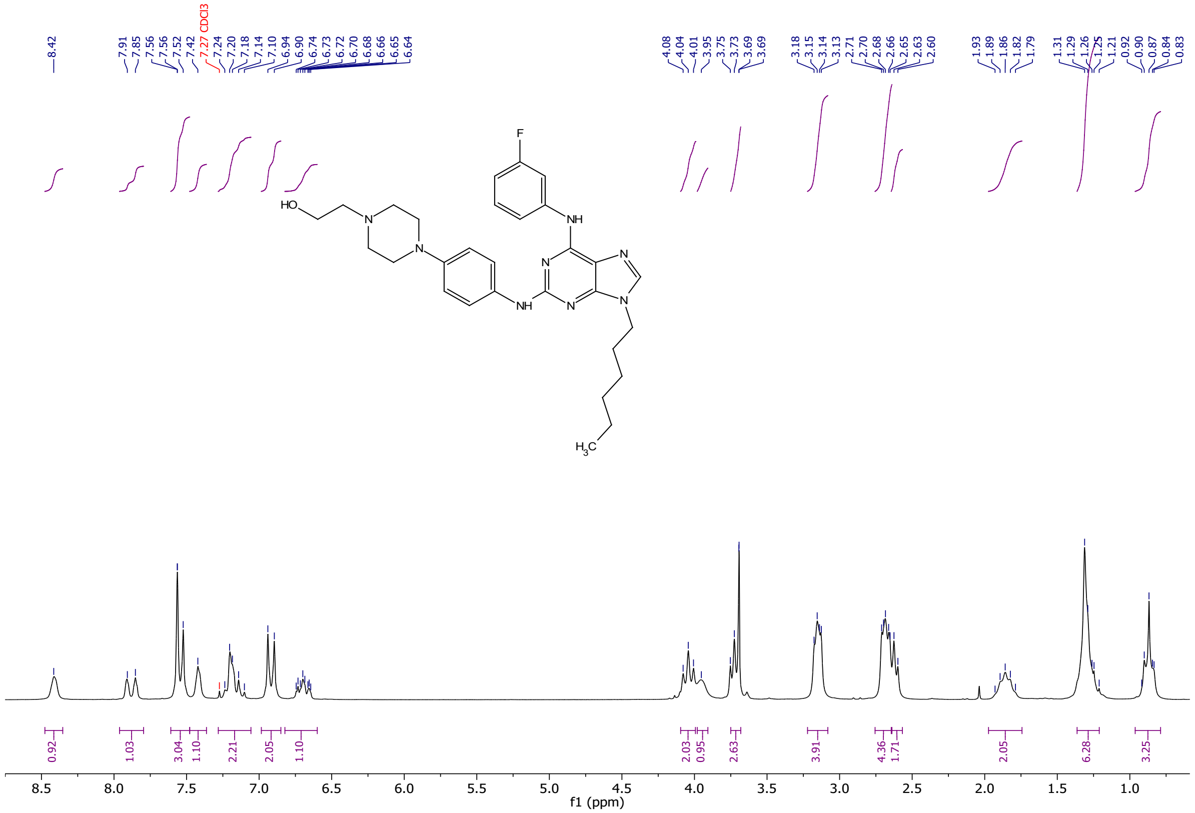

^{13}C NMR spectra of compound **11h**

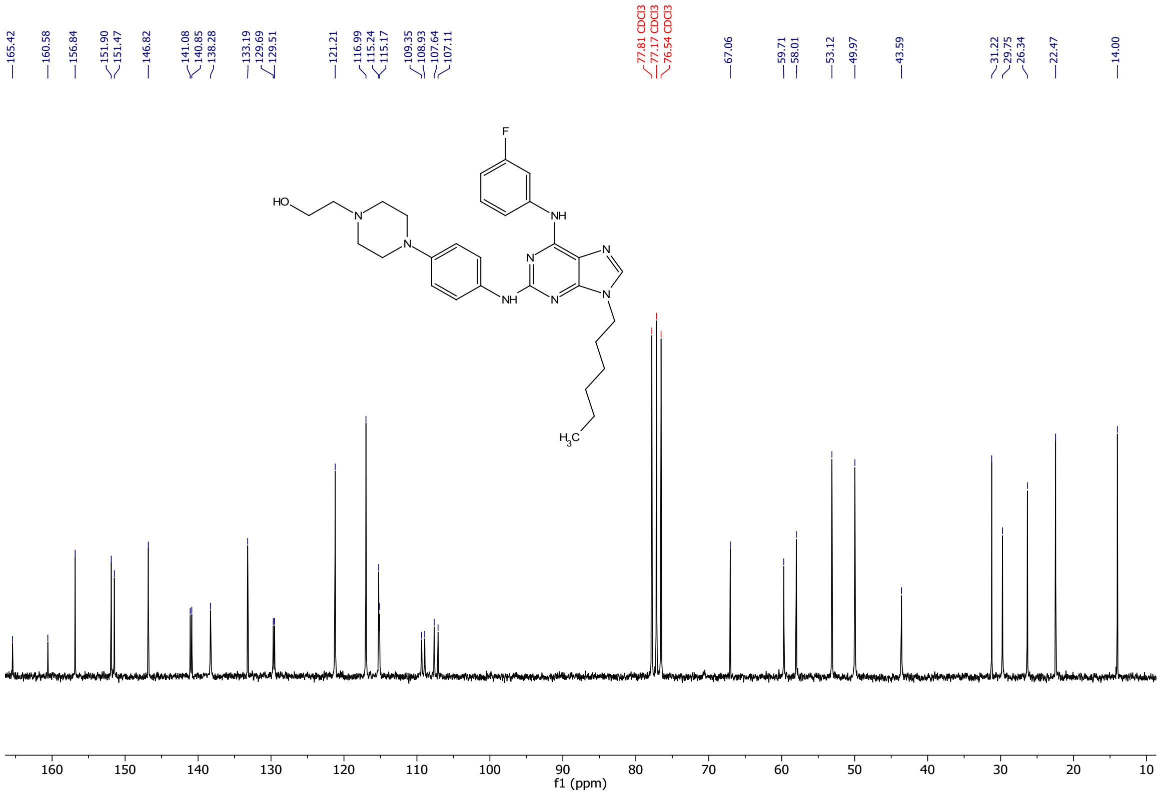

^{19}F NMR spectra of compound **11h**

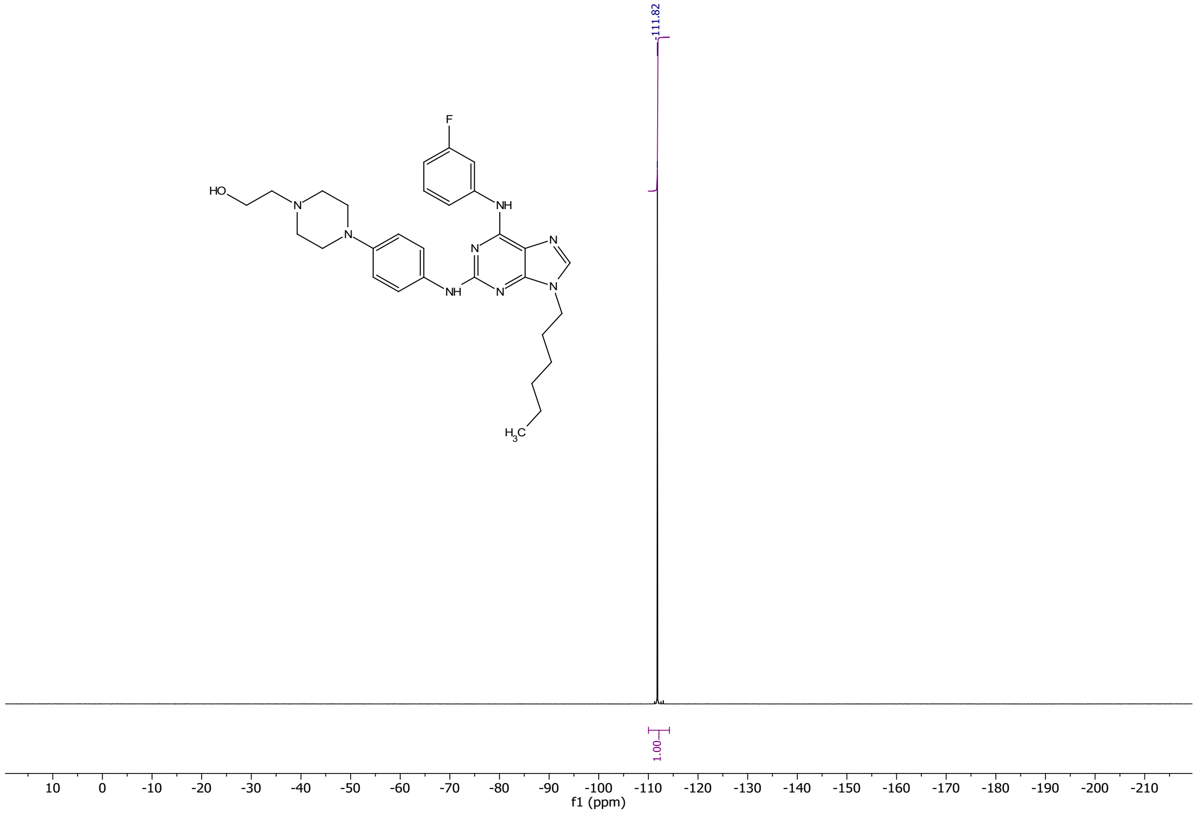

^{1}H NMR spectra of compound **11i**

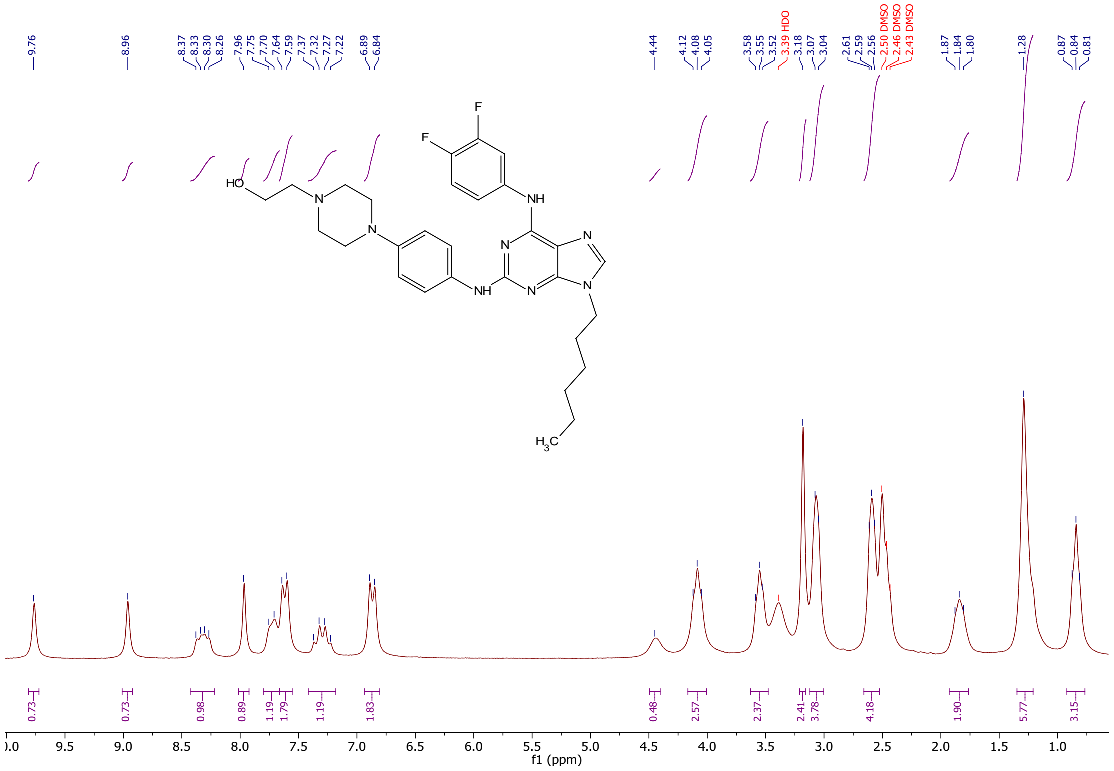

^{13}C NMR spectra of compound **11i**

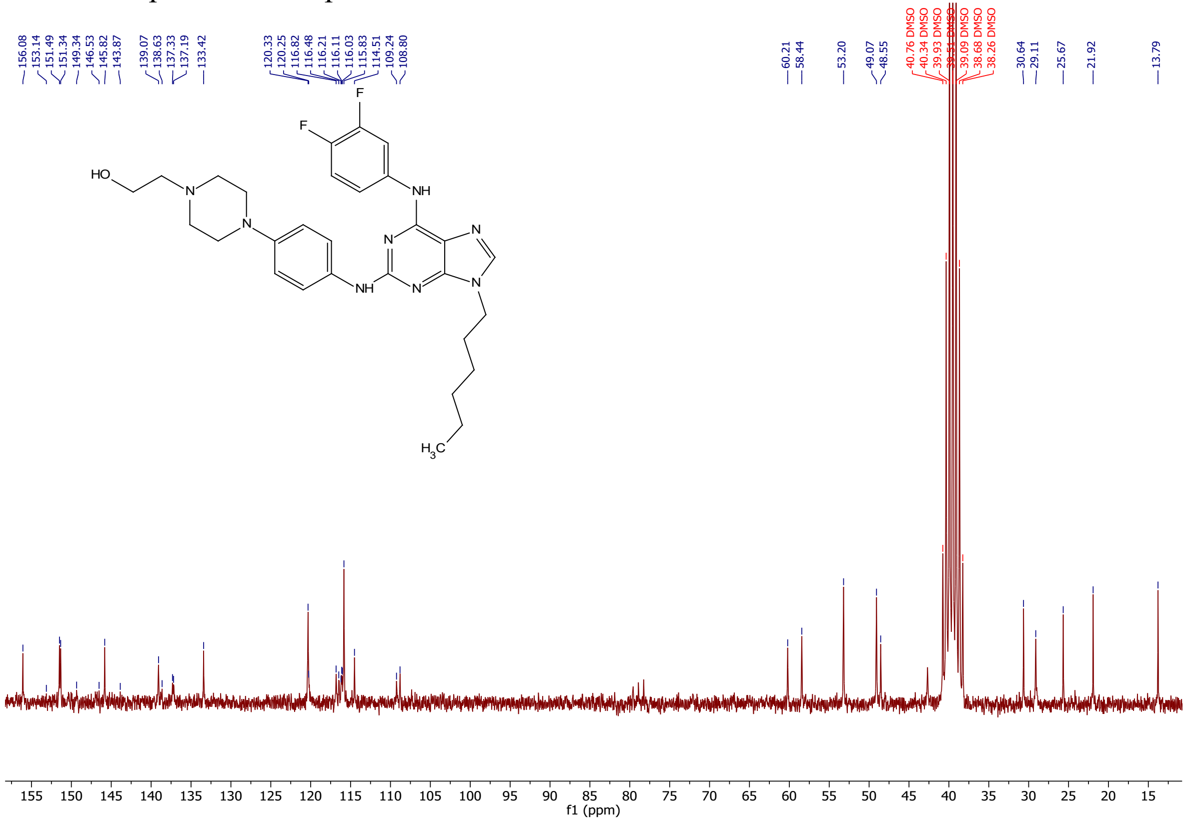

^{19}F NMR spectra of compound **11i**

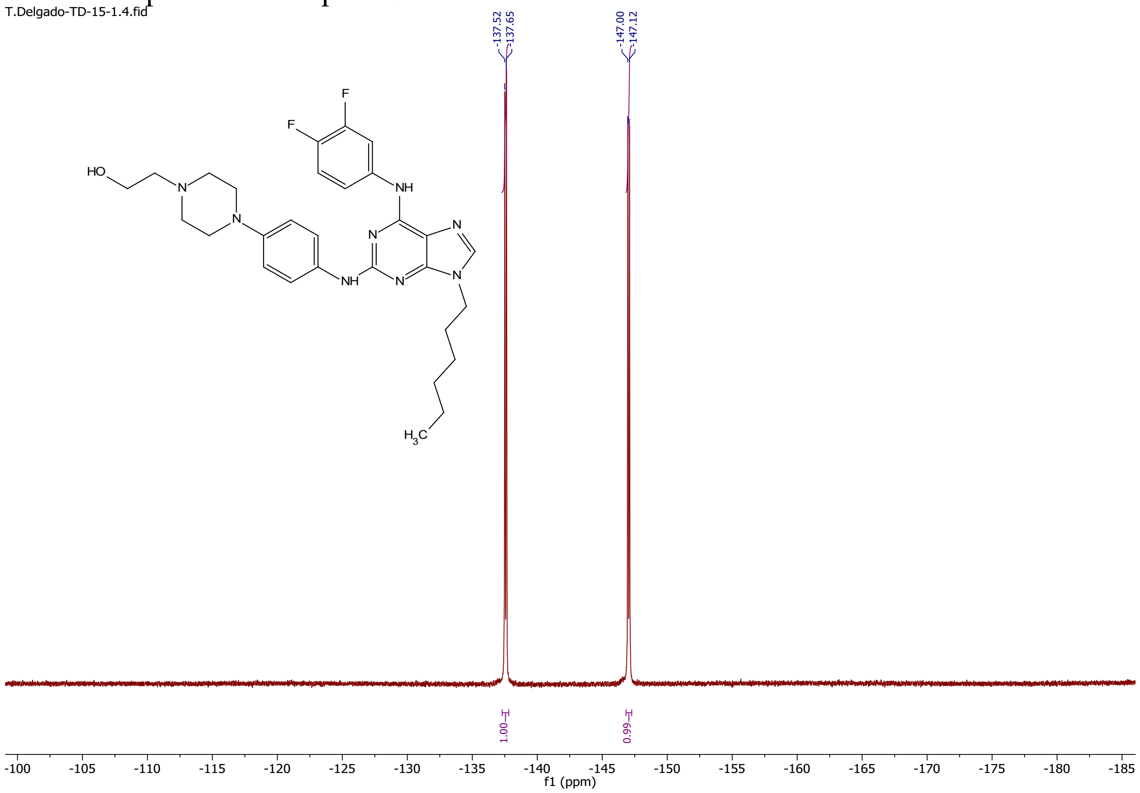

^{1}H NMR spectra of compound **13a**

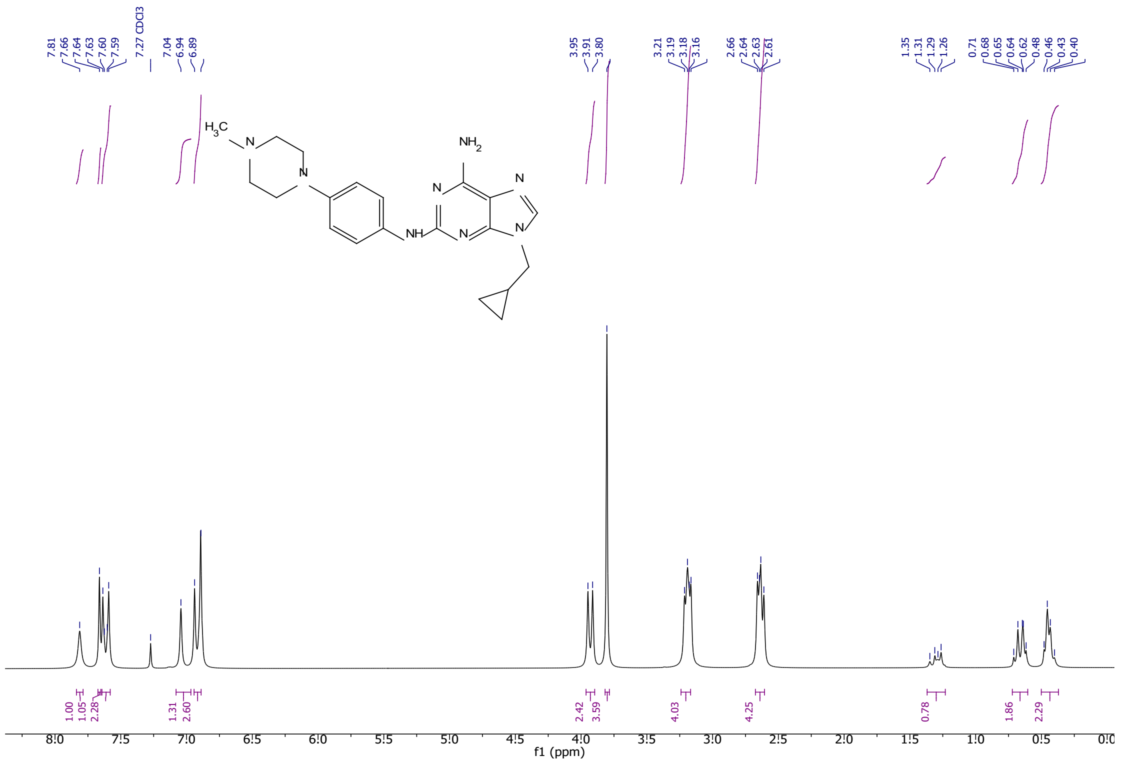

^{13}C NMR spectra of compound **13b**

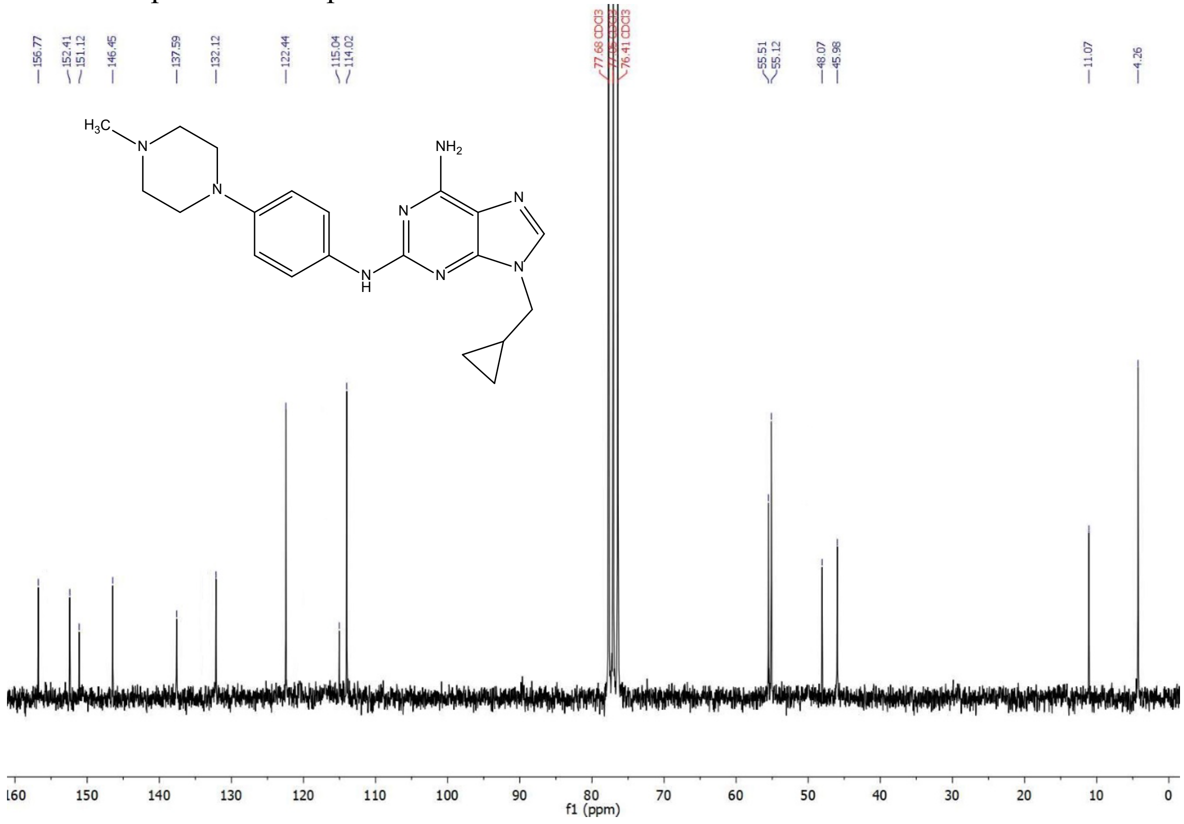

^{1}H NMR spectra of compound **13b**

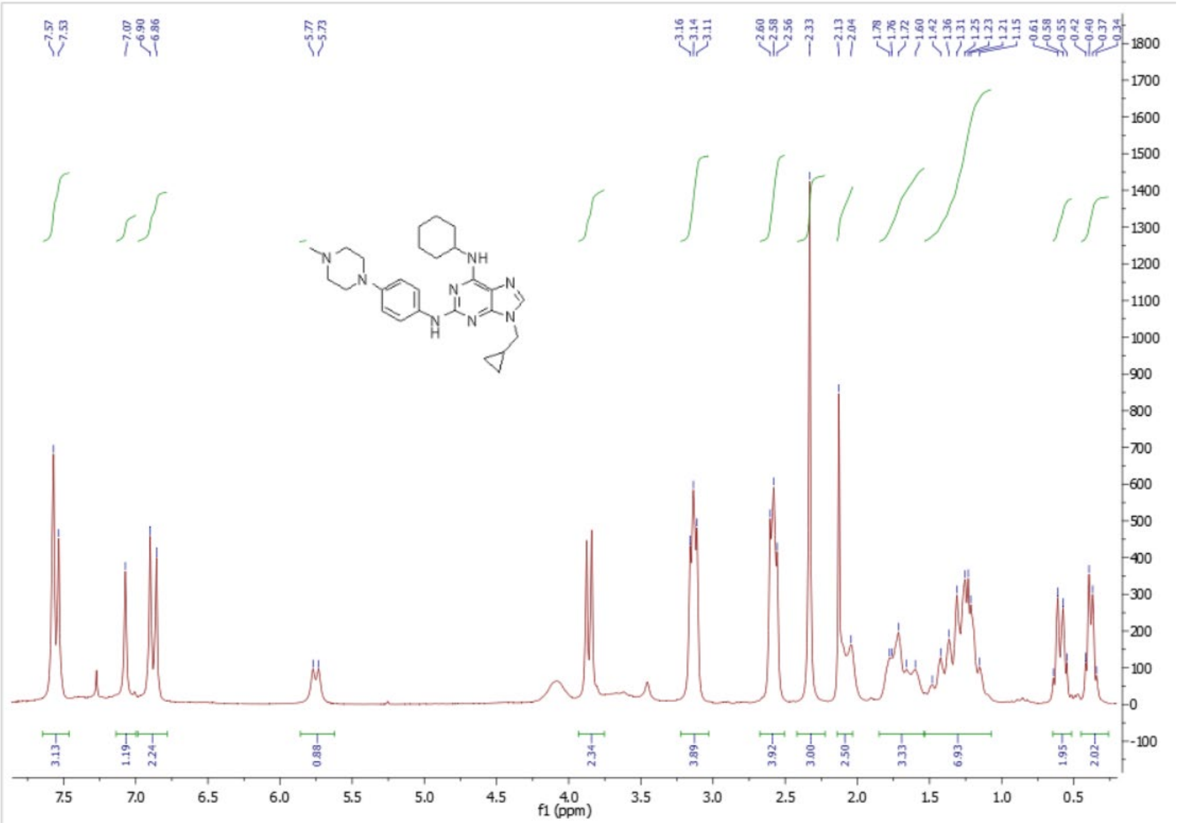

^{13}C NMR spectra of compound **13b**

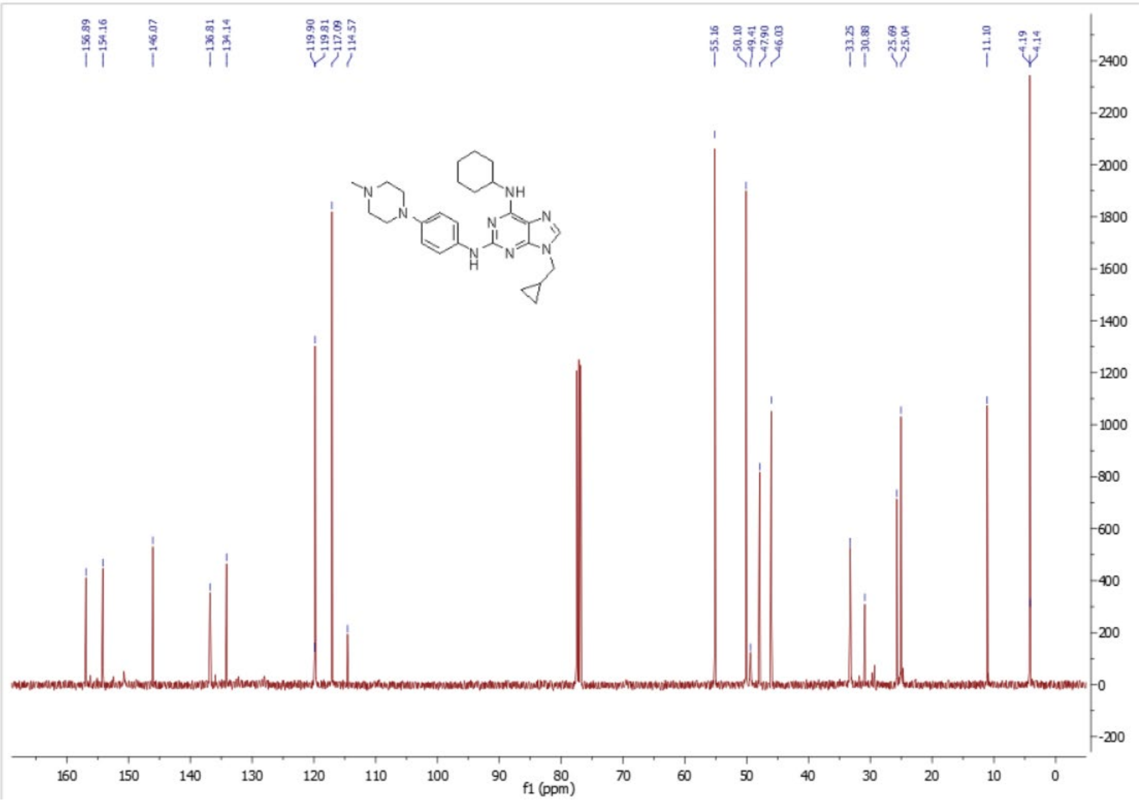

^{1}H NMR spectra of compound **13c**

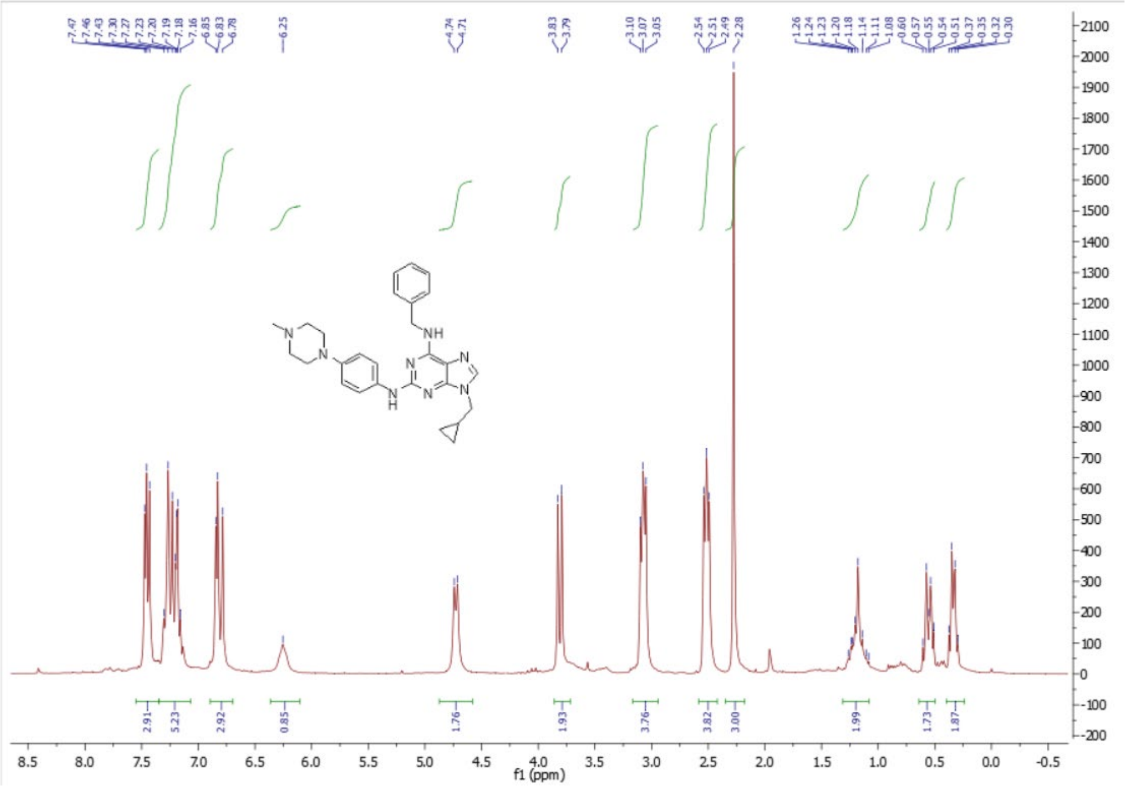

^{13}C NMR spectra of compound **13c**

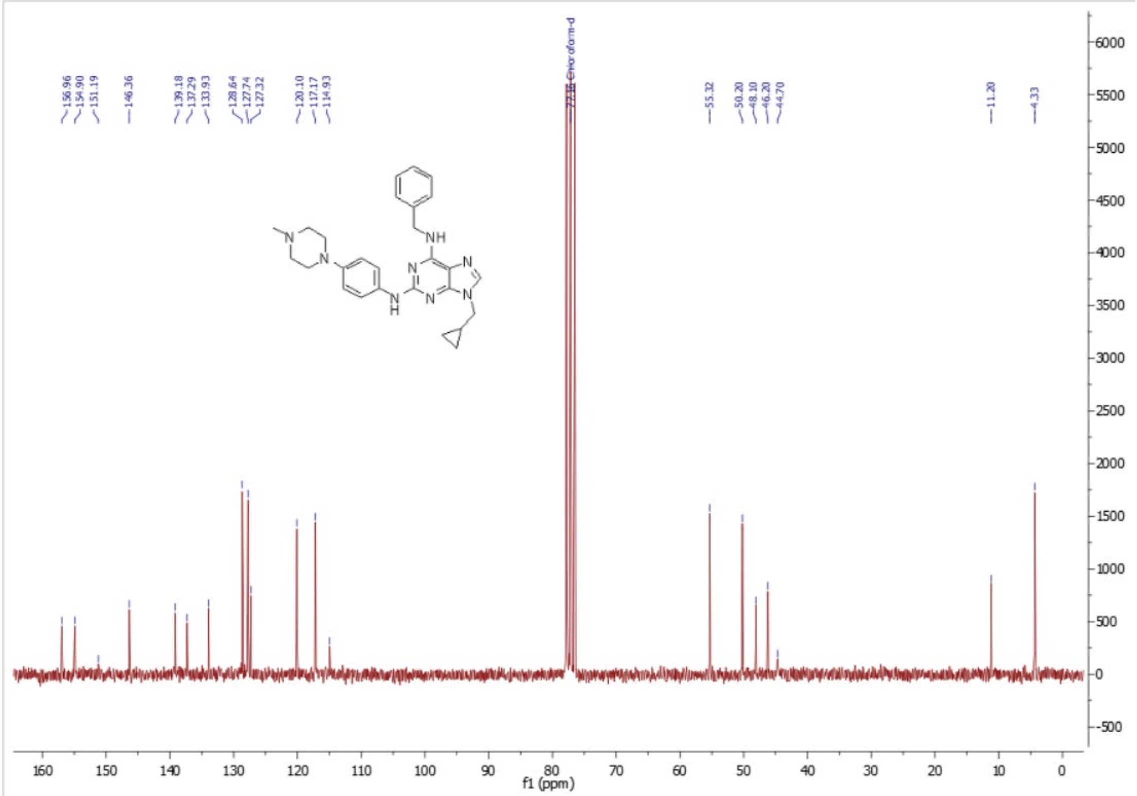

Mass spectra of compound **7a**

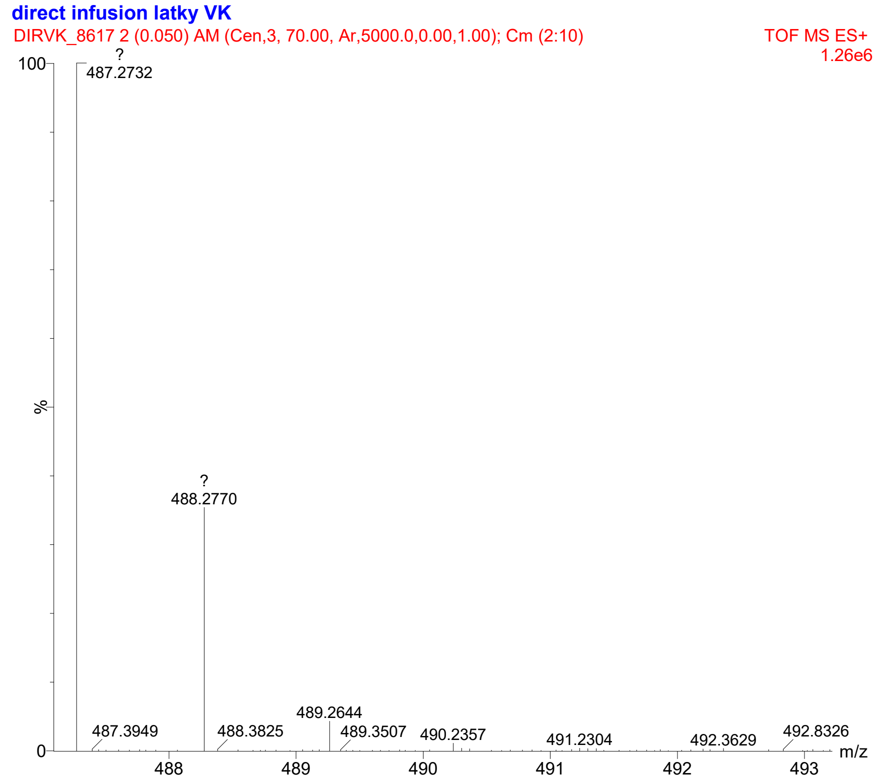

Mass spectra of compound **7b**

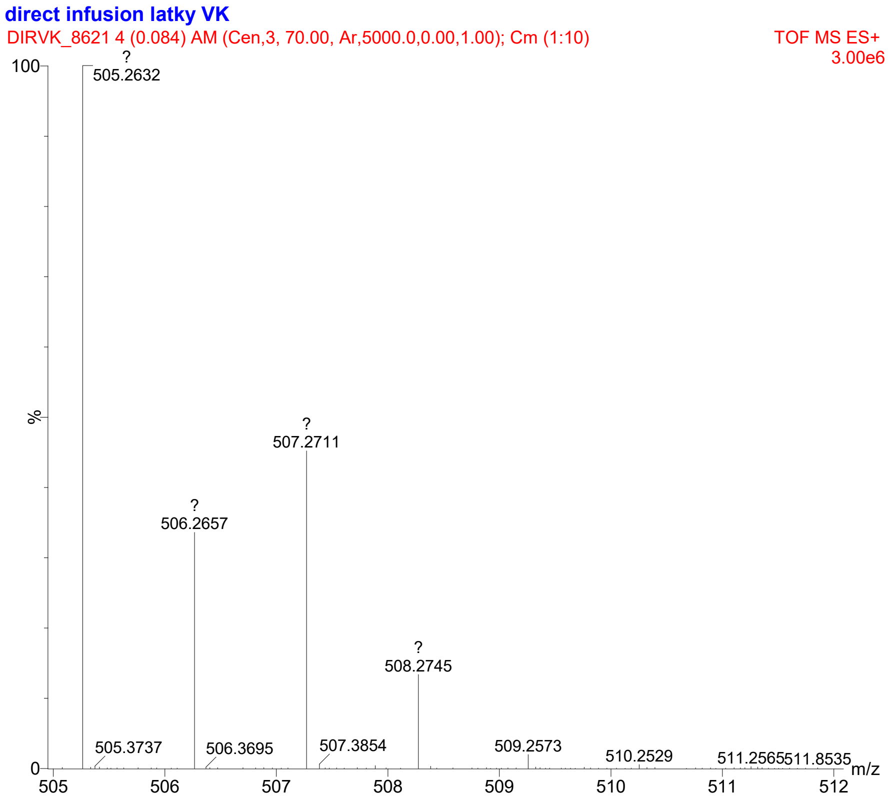

Mass spectra of compound **11a**

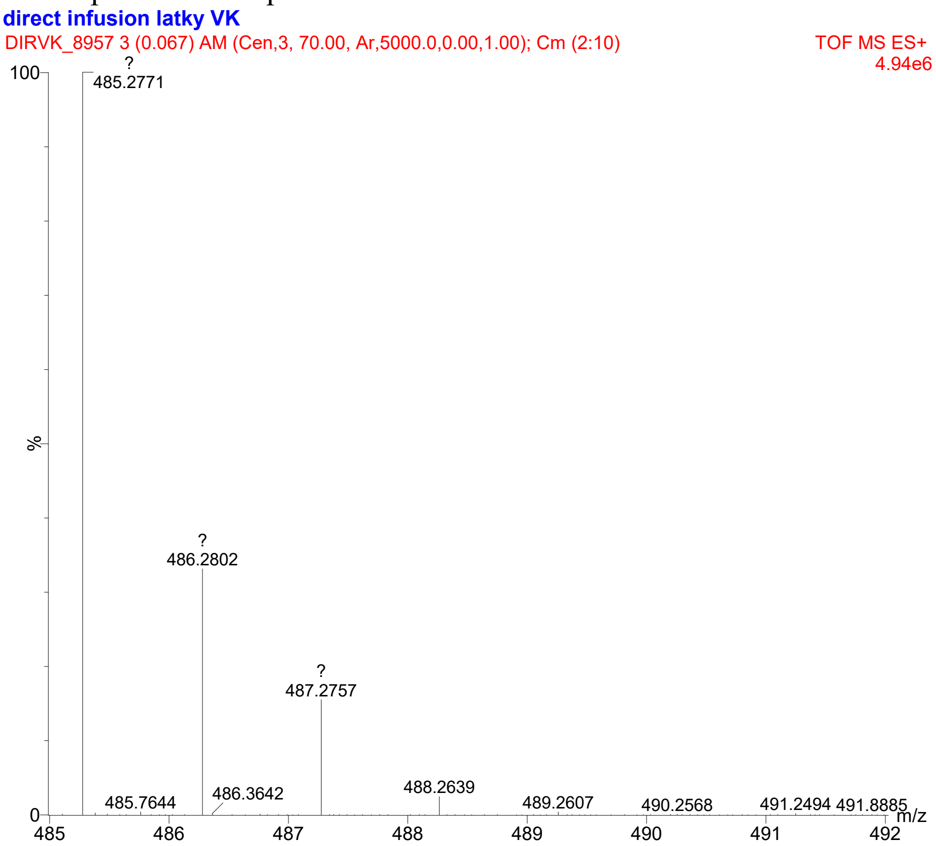

Mass spectra of compound **11b**

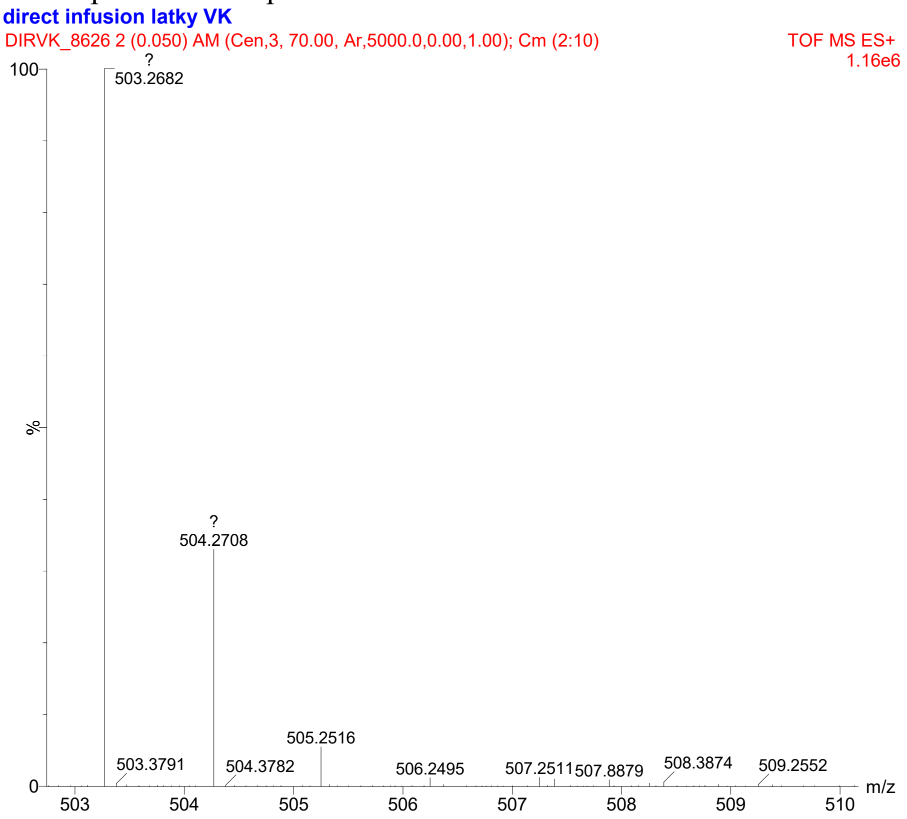

Mass spectra of compound **11c**

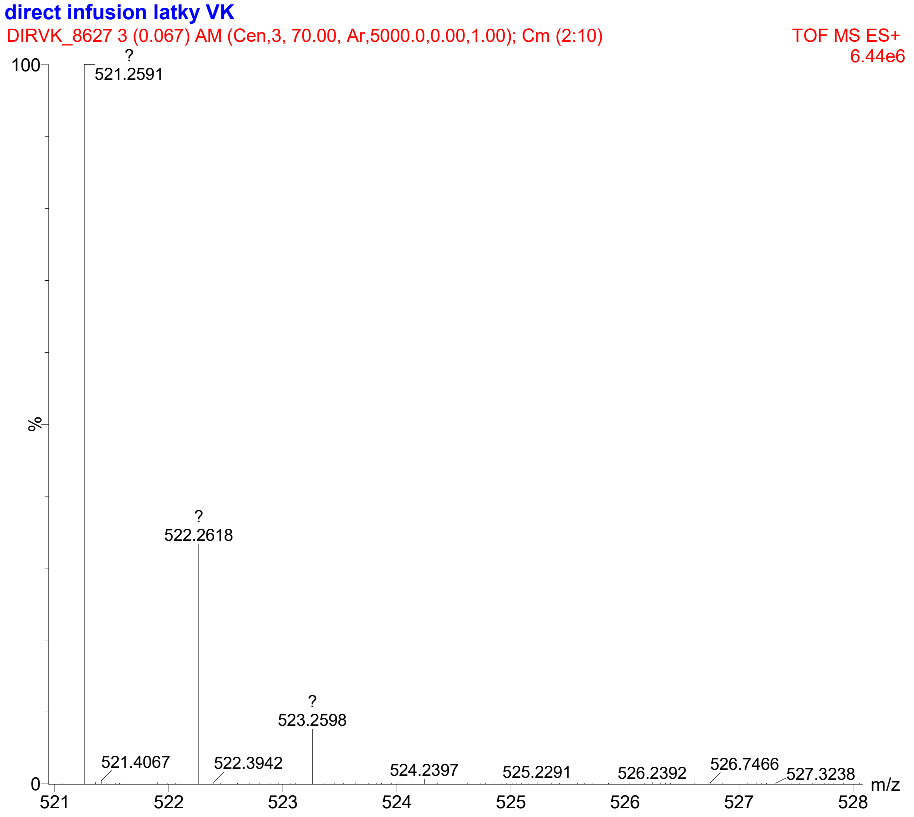

Mass spectra of compound **11d**

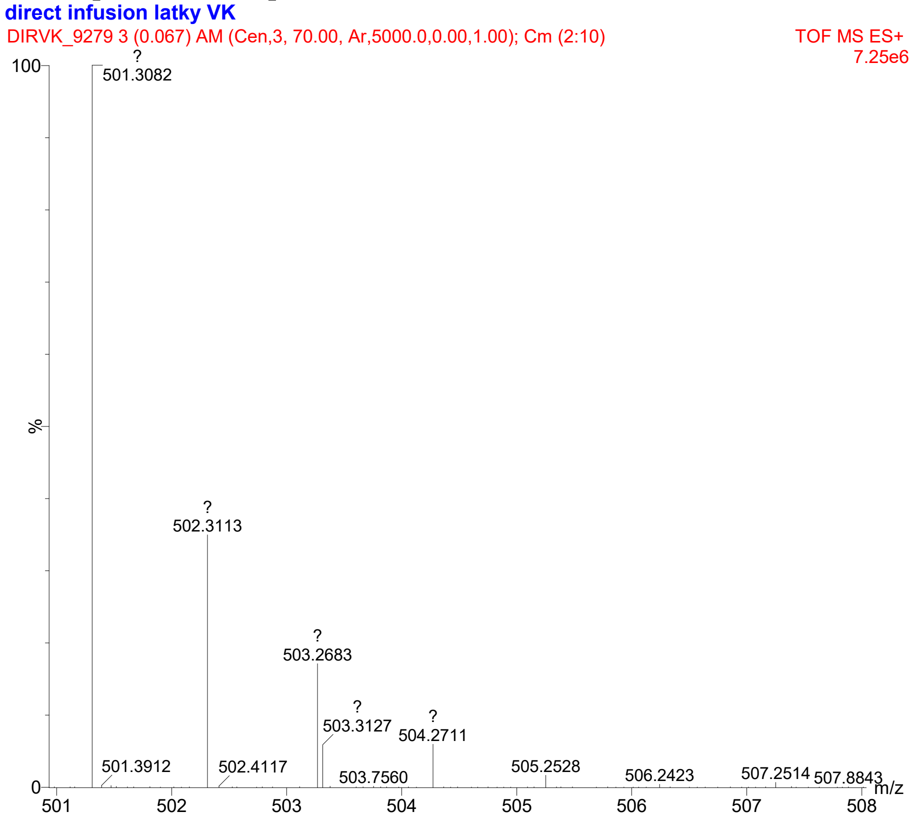

Mass spectra of compound **11e**

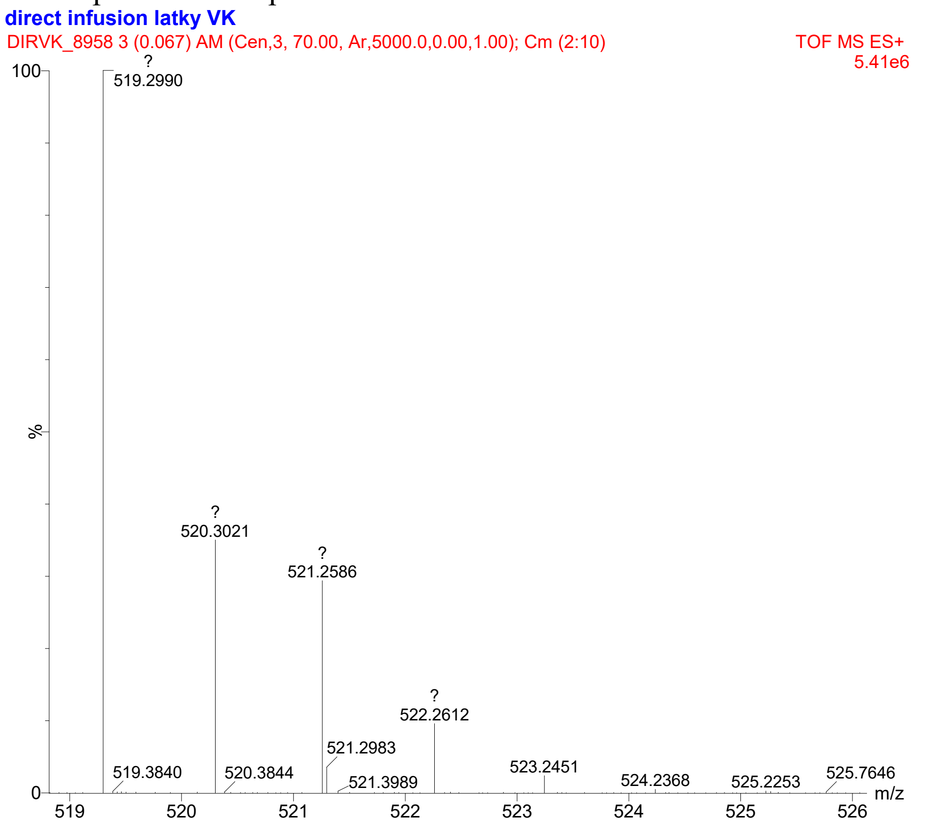

Mass spectra of compound **11f**

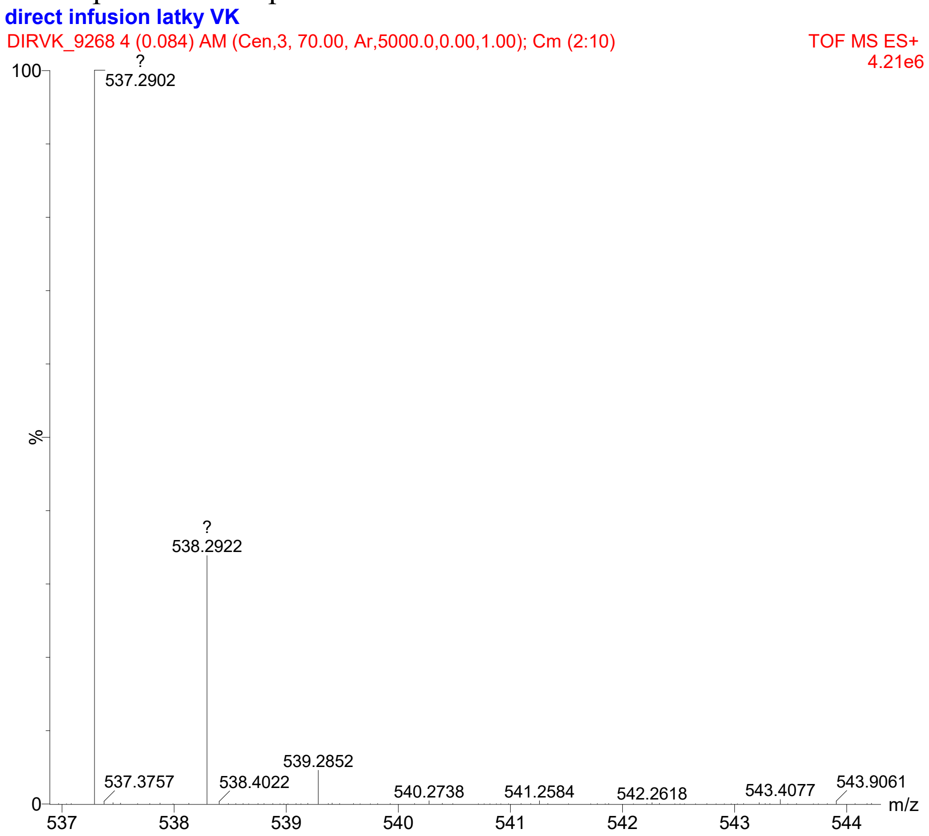

Mass spectra of compound **11g**

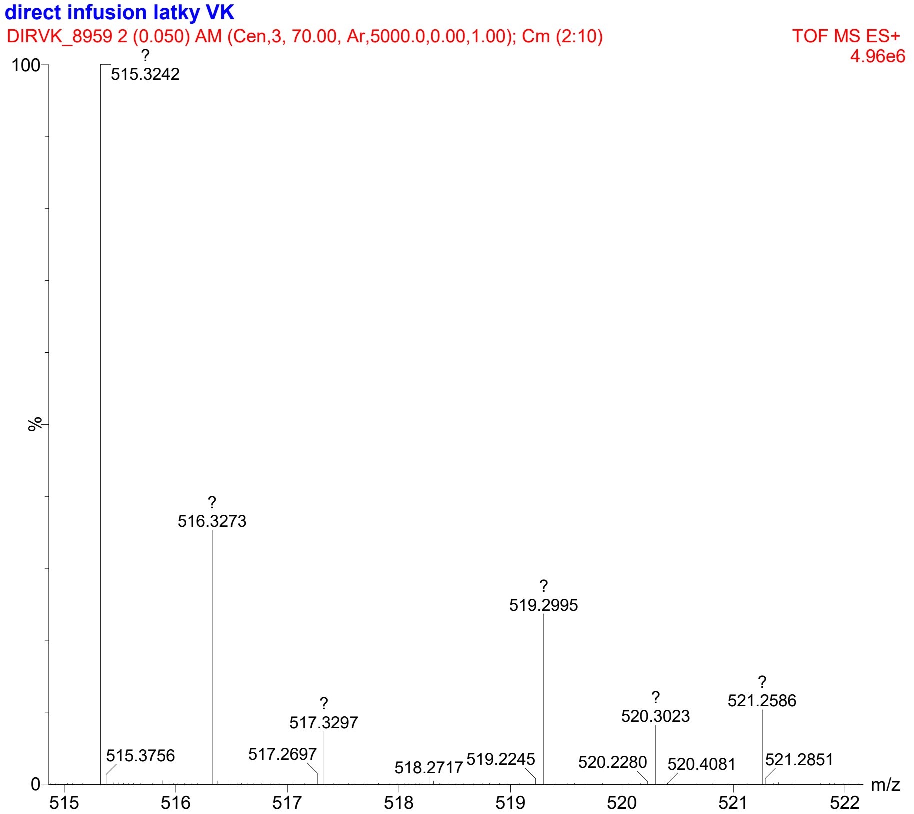

Mass spectra of compound **11h**

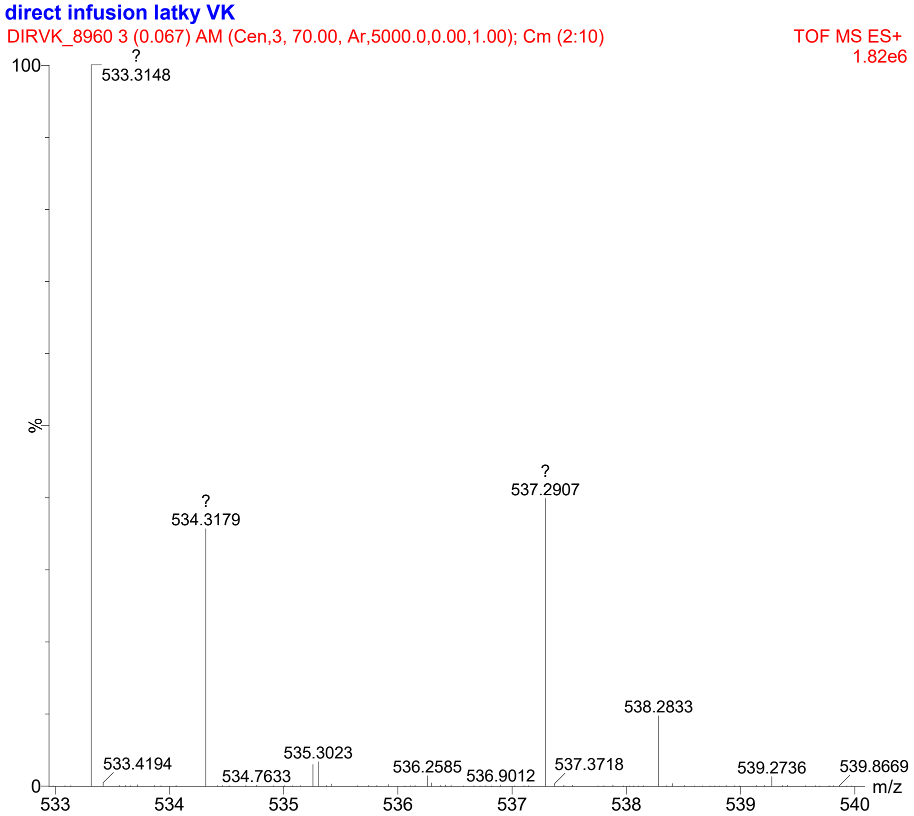

Mass spectra of compound **11i**

direct infusion latky VK

DIRVK_8628 2 (0.050) AM (Cen,3, 70.00, Ar,5000.0,0.00,1.00); Cm (2:10) TOF MS ES+ 2.50e6

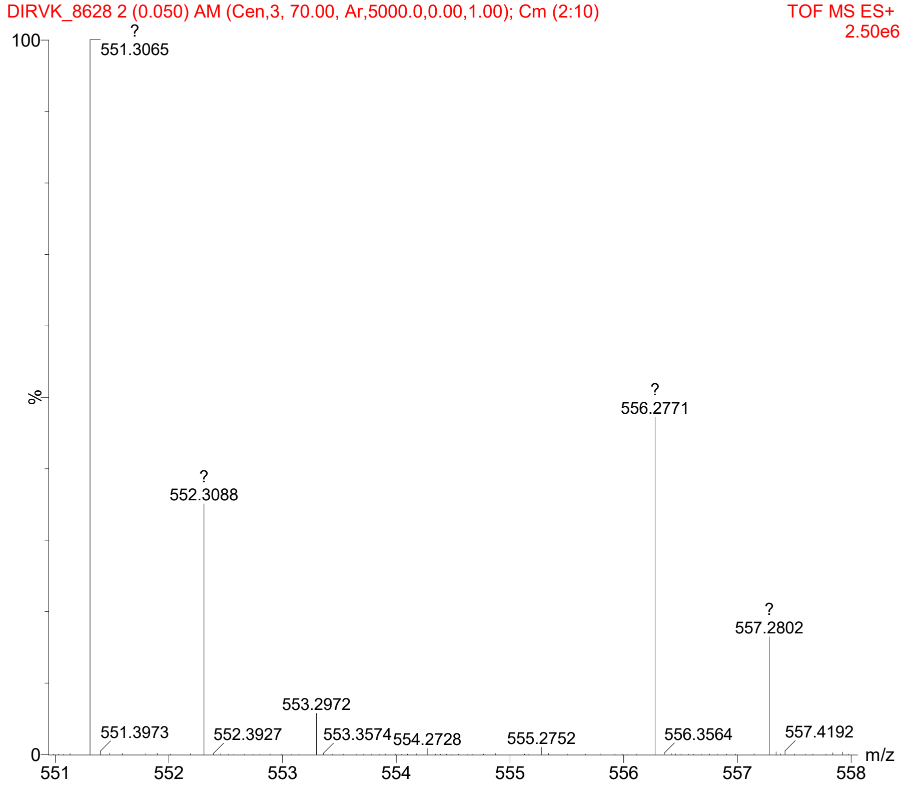

Mass spectra of compound **13a**

direct infusion latky VK

DIRVK_8618 4 (0.084) AM (Cen,3, 70.00, Ar,5000.0,0.00,1.00); Cm (1:9) TOF MS ES+ 2.08e7

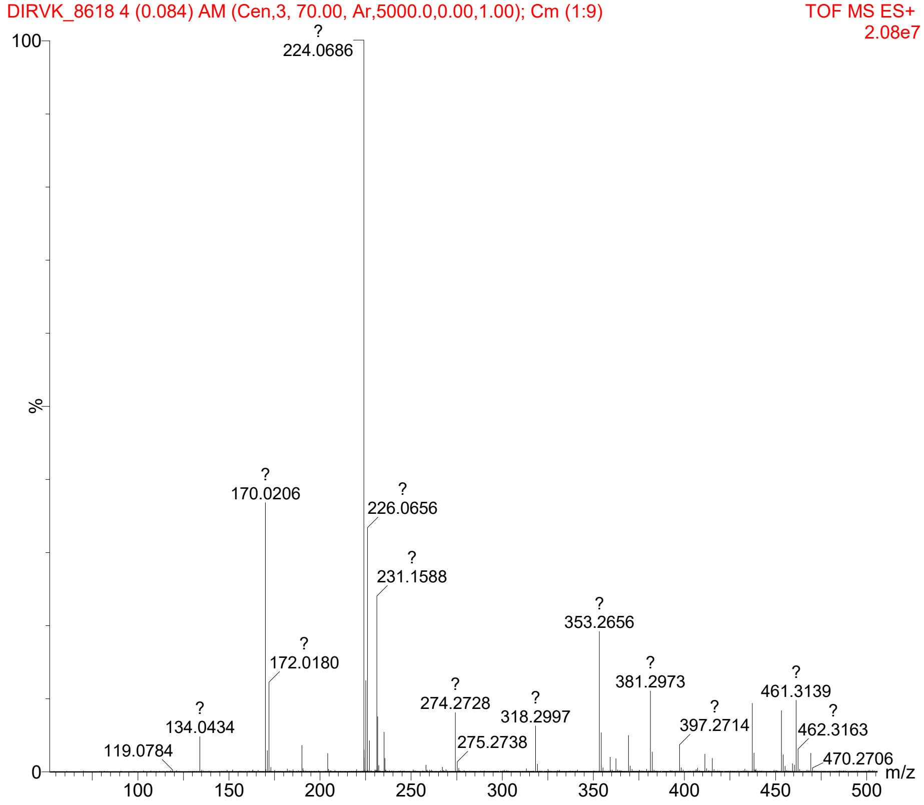

Mass spectra of compound **13b**

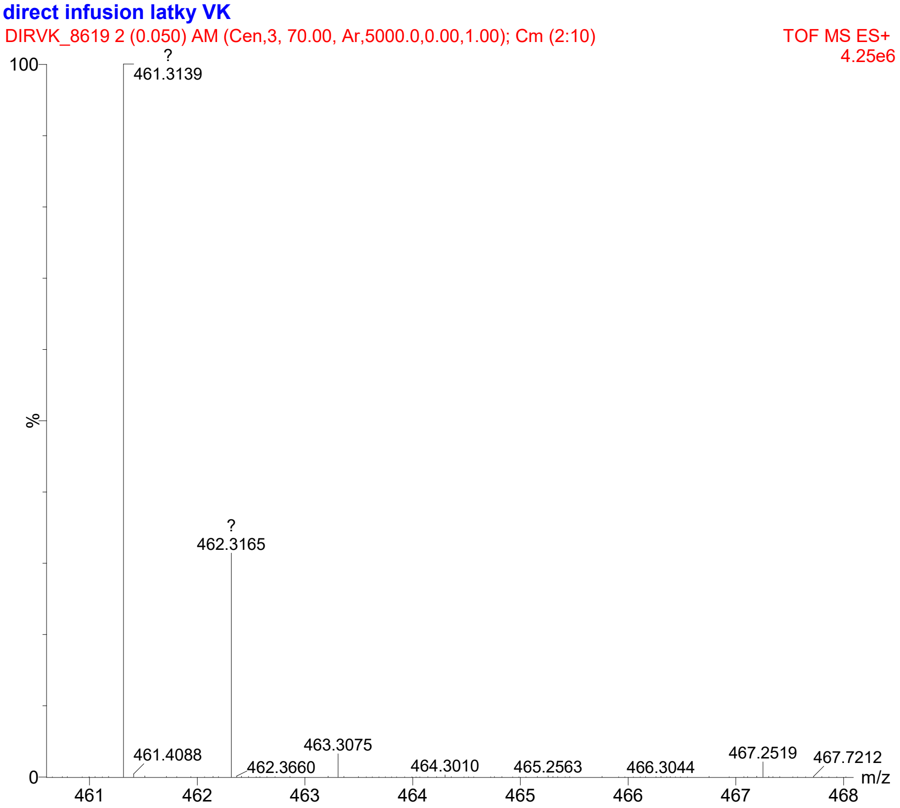

Mass spectra of compound **13c**

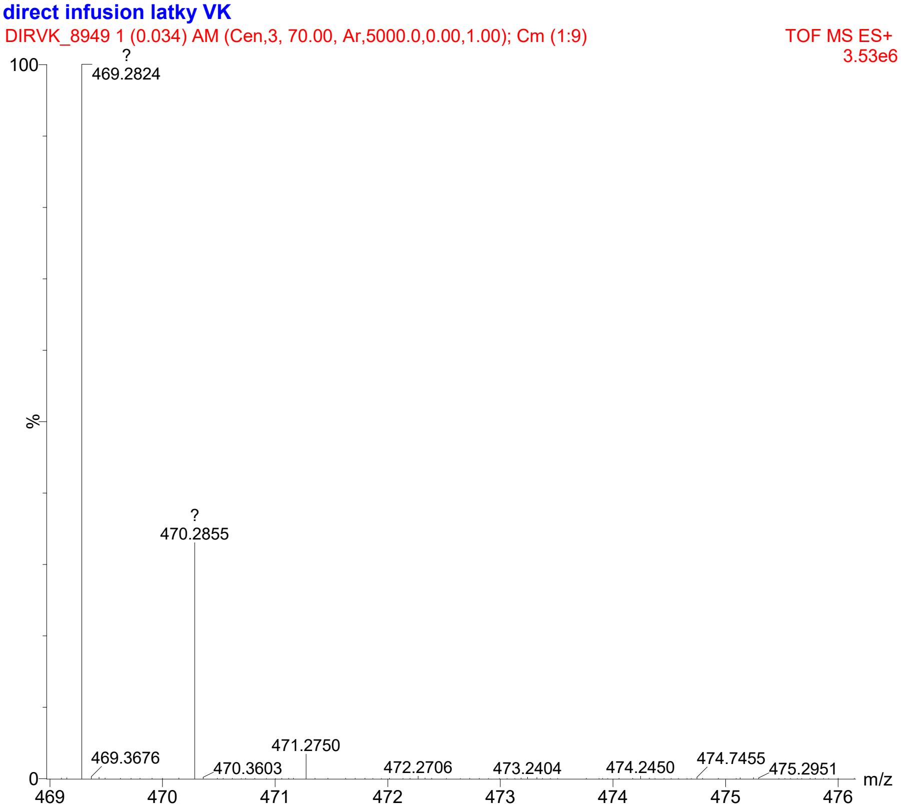

Table S1. Binding affinity scores (kcal/mol) of the purine derivatives with RDOCK, XP Score and MM-GBSA (ΔG_{bind} and their respective energy contributions) in the Bcr-Abl^{WT}.

Compd.	IC_{50}	pIC_{50}	RDOCK	XP Score	ΔG_{Bind}	$\Delta G_{Coul.}$	ΔG_{Hbond}	ΔG_{Lipo}	$\Delta G_{Packing}$	ΔG_{Solv_GB}	ΔG_{vdW}
**7b**	0.220	6.658	-15.66	-8.51	-64.21	-15.29	-1.16	-22.94	-0.79	26.87	-56.66
**7a**	0.180	6.745	-14.38	-9.16	-59.92	-40.76	-1.37	-21.14	-1.48	51.6	-59.46
**13c**	0.840	6.076	-15.31	-7.74	-50.43	-15.57	-1.69	-19.3	-1.05	26.14	-51.97
**13b**	11.050	4.957	-14.22	-7.57	-51.13	-41.41	-1.76	-17.41	-1.07	47.1	-45.27
**13a**	3.950	5.403	-13.94	-7.51	-48.57	-8.79	-1.3	-16.44	-0.59	15.37	-47.62
**11i**	1.680	5.775	-15.15	-6.91	-55.34	-3.88	-2.29	-29.12	-0.66	19.24	-51.52
**11h**	0.670	6.174	-15.39	-7.33	-53.75	-10.36	-1.54	-20.23	-0.75	20.36	-50.32
**11g**	0.830	6.081	-13.19	-7.25	-52.36	-9.56	-1.47	-19.50	-0.70	20.50	-49.50
**11f**	1.760	5.754	-17.66	-8.57	-66.75	-18.34	-2.44	-24.09	-1.59	31.97	-53.41
**11e**	0.130	6.886	-17.33	-8.40	-65.32	-17.65	-2.30	-23.31	-1.47	30.23	-52.14
**11d**	1.240	5.907	-16.84	-8.20	-60.11	-16.48	-2.01	-22.32	-1.40	28.06	-51.04
**11c**	0.019	7.721	-19.04	-9.11	-74.32	-23.49	-2.7	-20.95	-0.89	24.31	-54.69
**11b**	0.014	7.854	-20.69	-9.36	-73.34	-24.39	-2.69	-20.92	-0.89	26.22	-54.71
**11a**	0.037	7.432	-17.49	-9.23	-71.06	-26.33	-3.07	-21.6	-1.16	29.25	-57.85
**I**	0.090	7.046	-15.19	-8.54	-57.16	-43.55	-1.24	-20.24	-1.23	48.37	-47.55
**II**	0.040	7.398	-16.91	-8.83	-60.04	-37.06	-0.83	-21.32	-1.53	45.94	-55.10
**III**	0.040	7.398	-16.54	-9.57	-59.74	-39.82	-1.19	-20.50	-1.24	42.57	-49.56
**Purvalanol***	—	—	-31.45	-9.60	-69.69	-18.25	-1.90	-18.51	-1.46	15.06	-46.15
**Dasatinib****	0.00014	9.854	—	-13.91	-87.55	-36.72	-2.12	-25.85	0.92	43.96	-58.09

ΔG_{Bind} = Binding free energy
ΔG_{Coul} = Coulomb energy
ΔG_{Hbond} = Hydrogen bonding correction
ΔG_{Lipo} = Lipophilic energy
$\Delta G_{Packing}$ = Pi-pi packing correction
$\Delta G_{Solv\ GB}$ = Generalized Born electrostatic solvation energy
ΔG_{vdW} = van der Waals energy
ΔGBind = Binding free energy
*Docking score calculated with RDOCK, PDB: 6BL8
**Docking score calculated with PDB 2GQG

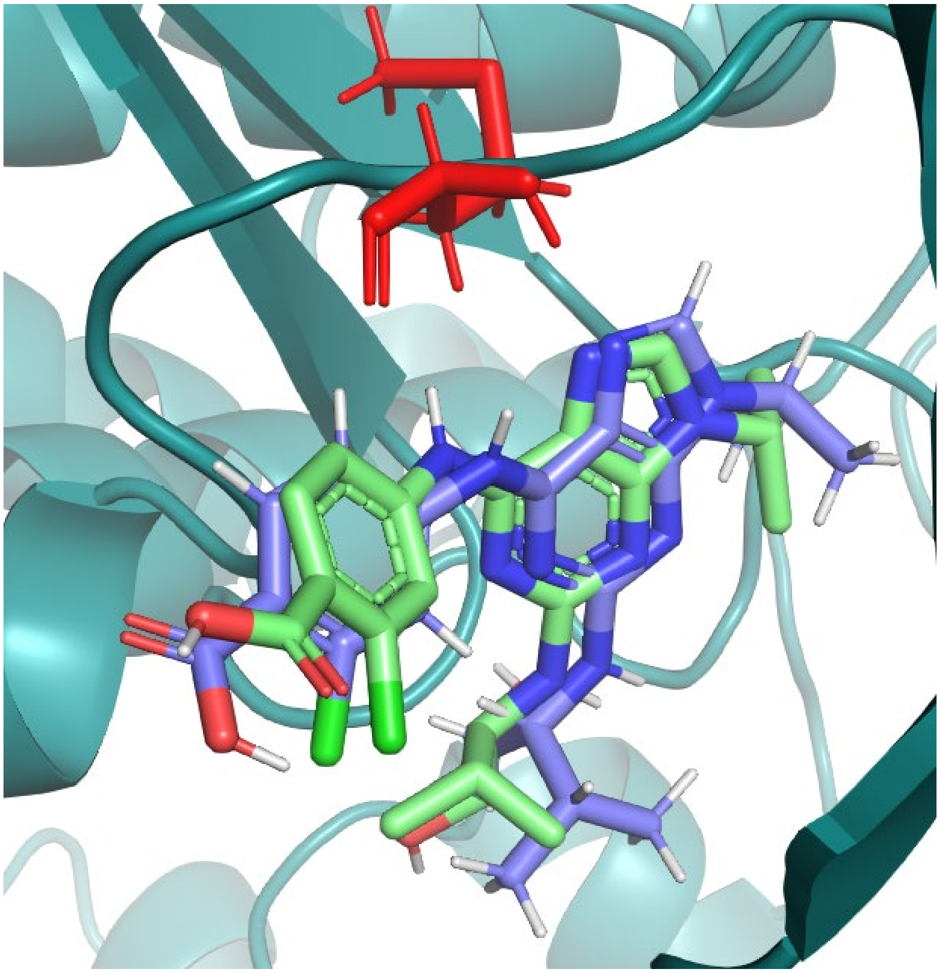

Figure S1. Co-crystallized ligand, purvalanol, in the Bcr-Abl binding site. Its experimentally determined binding mode is shown in blue, while the docking pose of purvalanol from our self-docking protocol is shown in green.

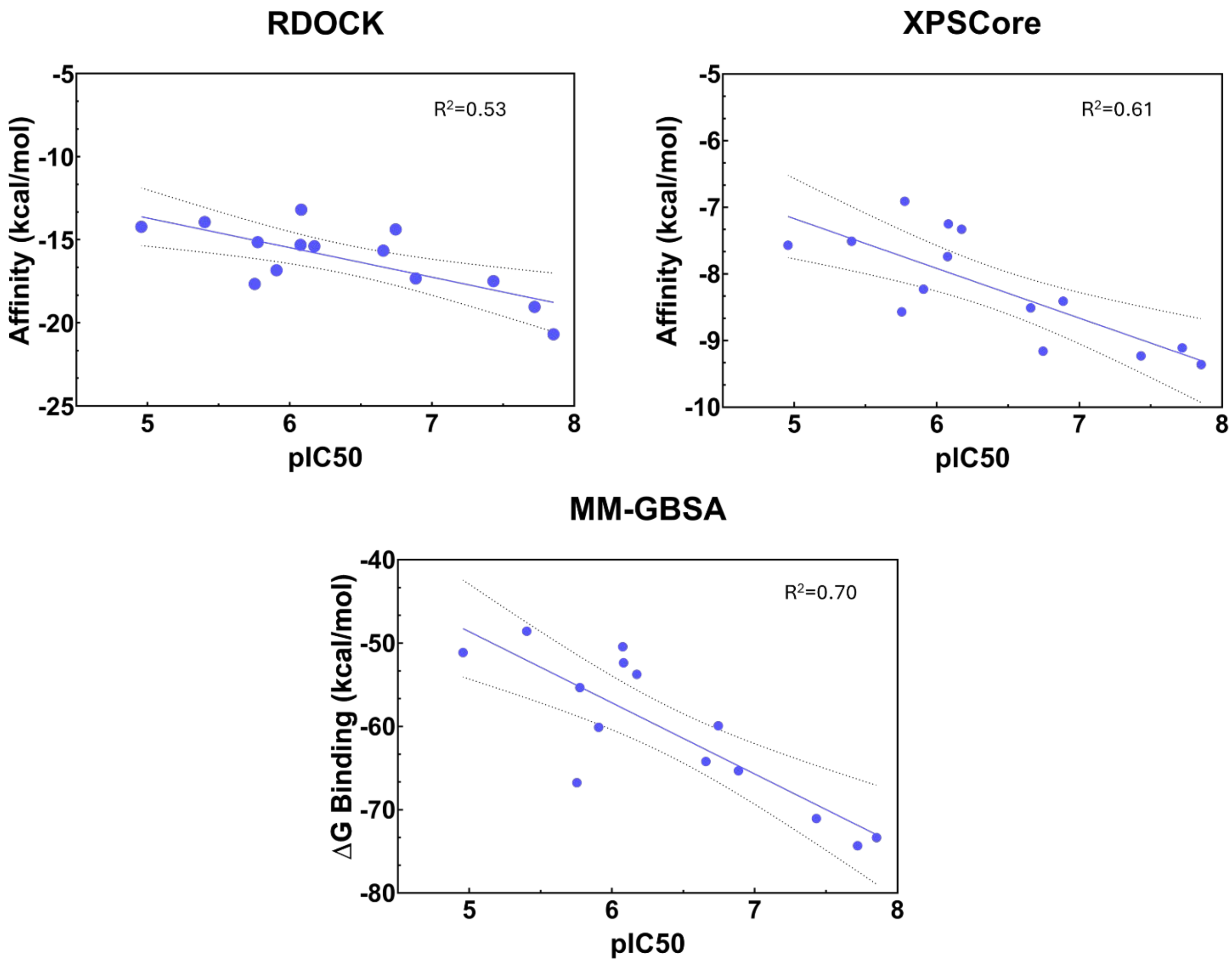

Figure S2. Correlation plot between biological activity in pIC_{50} and affinity energies achieved with RDOCK, Glide (XP Score) and MM-GBSA of the synthesised compounds.

Comp.	T315I (100%) **KCL22B8 GI50 (uM)**	SP Score	MM-GBSA
**11c**	9.06	-11.14	-65.17
**11d**	7.83	-11.48	-69.82
**11e**	6.91	-12.18	-71.67
**11f**	7.20	-12.12	-70.14

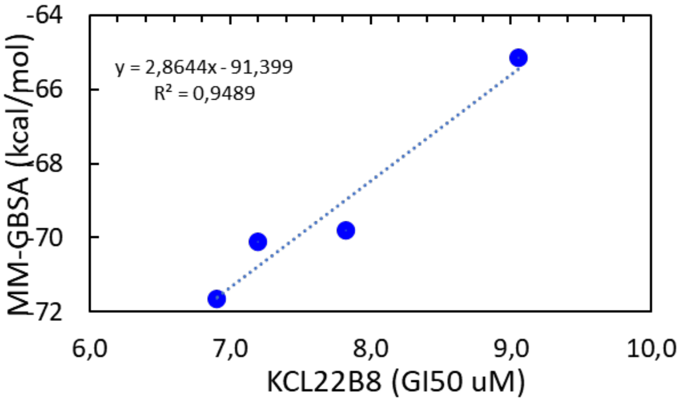

Figure S3. Correlation plot between biological activity in pGI_{50} values on B8 cells and affinity energies achieved with MM-GBSA of the selected compounds.

Figure S4. Histograms of flow cytometry of selected compounds on KCL cells and their subclones.

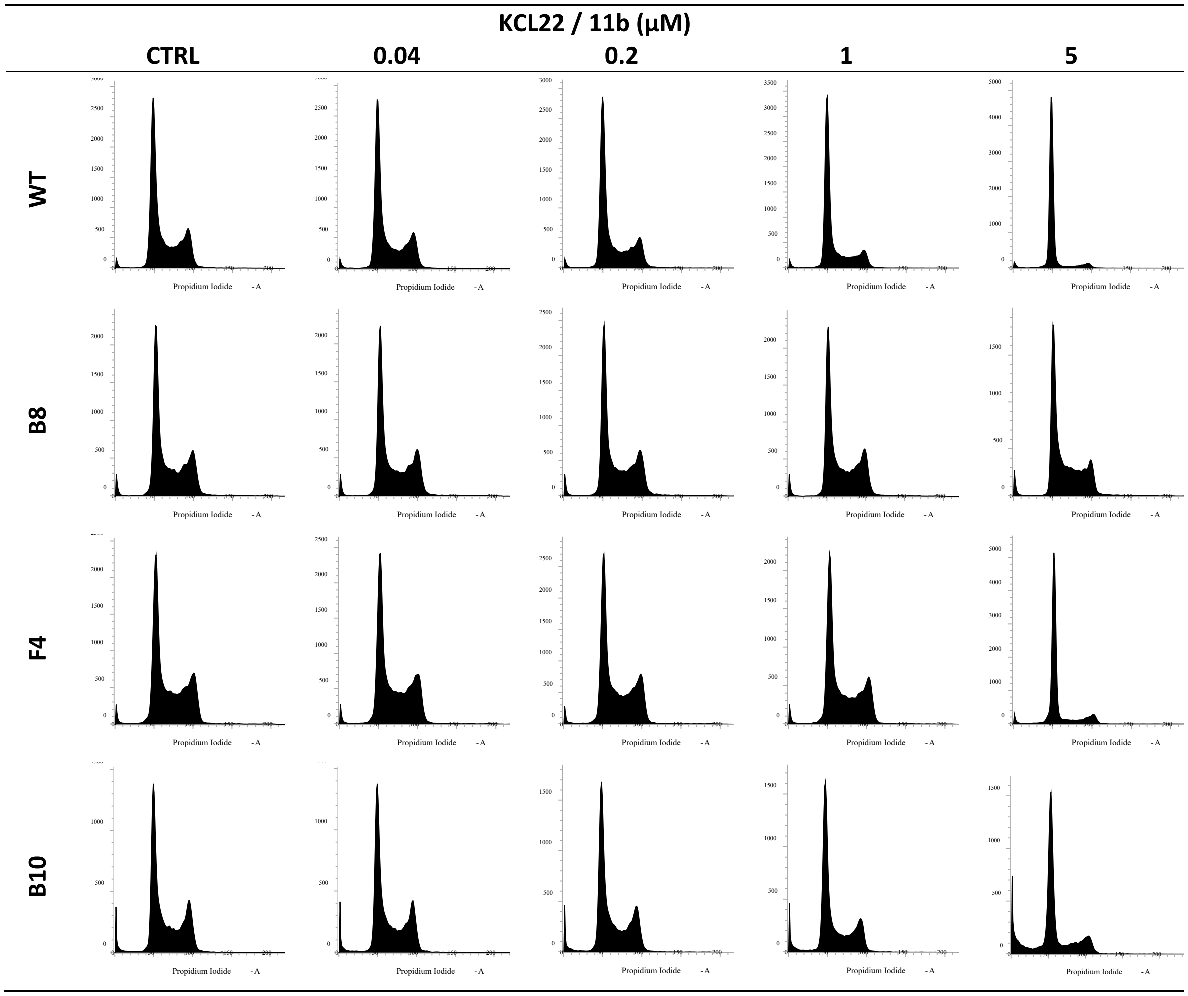

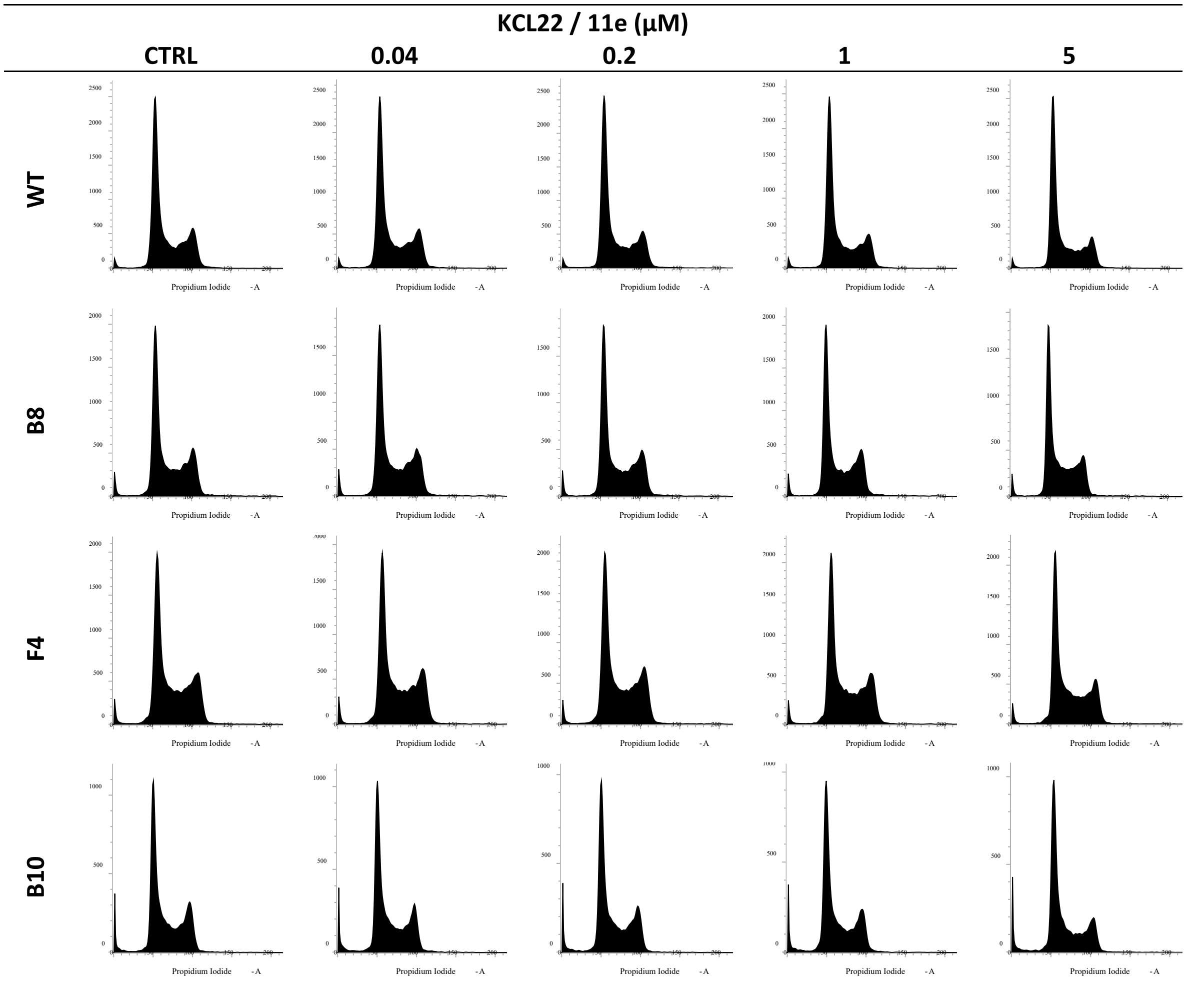
KCL22 / 11e (µM)
CTRL
0.04
0.2
1
5
WT
B8
F4
B10
Propidium Iodide -A

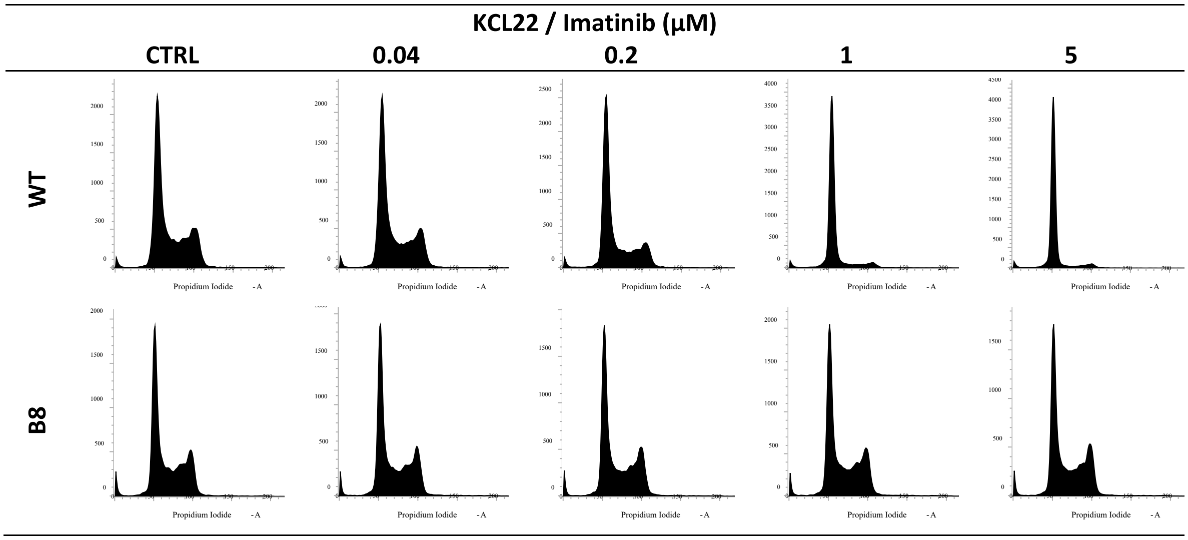
KCL22 / Imatinib (µM)
CTRL
0.04
0.2
1
5
WT
B8
Propidium Iodide -A

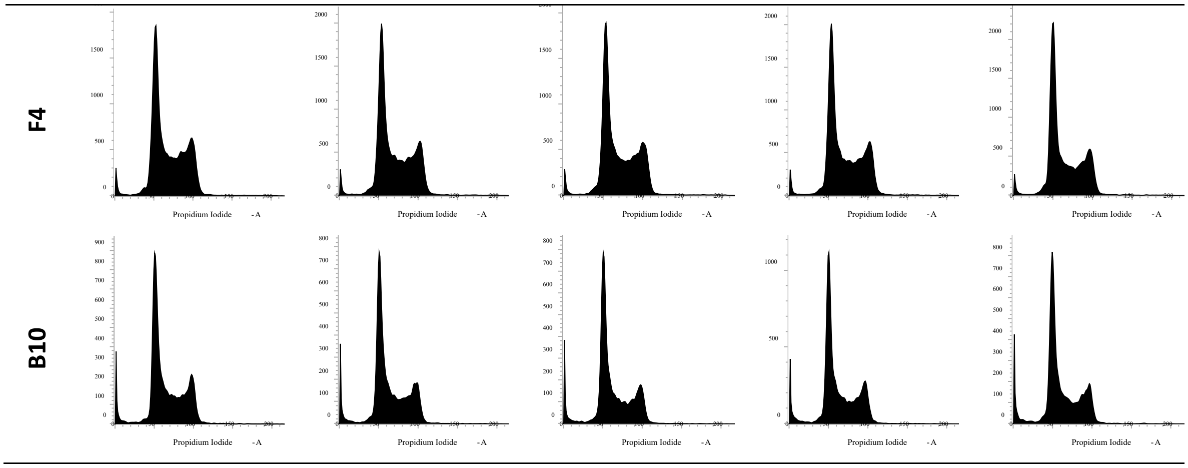

KCL22 / Nilotinib (µM)

CTRL	0.04	0.2	1	5

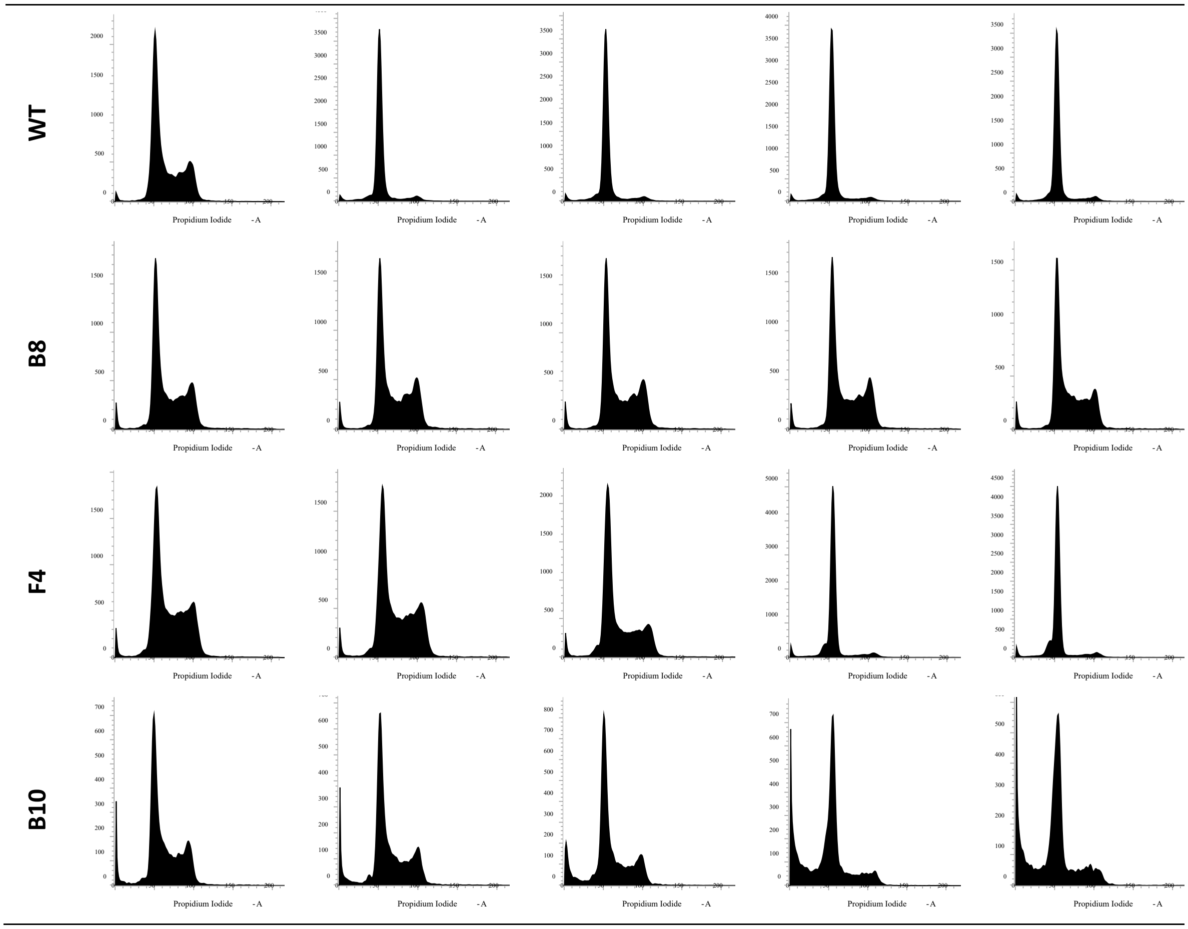

Supplement: Supplementary file 1 [file pharmaceutics-16-00649-s001.zip › pharmaceutics-2979761-supplementary.pdf]
